# Superconductivity from quasiparticle pairing of intervalley coherent state in rhombohedral trilayer graphene

**DOI:** 10.1038/s41467-026-72135-y

**Published:** 2026-05-28

**Authors:** Chun Wang Chau, Shuai A. Chen, K. T. Law

**Affiliations:** 1 Department of Physics, Hong Kong University of Science and Technology, Clear Water Bay, Hong Kong, China; 2 Theory of Condensed Matter Group, Cavendish Laboratory, University of Cambridge, Cambridge, UK; 3 Max Planck Institute for the Physics of Complex Systems, 01187 Dresden, Germany

**Keywords:** Superconducting properties and materials, Electronic properties and materials

## Abstract

Superconductivity is observed in rhombohedral trilayer graphene in a narrow regime between the flavour-symmetric state and the symmetry breaking phase, which cannot be described by the conventional Bardeen-Cooper-Schrieffer theory. The measured coherence length, for instance, is roughly two orders of magnitude shorter than the value predicted by the Bardeen-Cooper-Schrieffer relation based on the large fermi velocity and an extremely low charge carrier density of the flavour-symmetric phase. To resolve the discrepancies, we propose that the rhombohedral trilayer graphene superconducting phase arises from the pairing of quasiparticles of the adjacent inter-valley coherent state. We illustrate the superconducting phenomenology using gapped Dirac cones with the chemical potential *μ* close to the valence band’s edge. Our findings indicate that the transition temperature *T*_*c*_ obeys ${T}_{c}\propto {\epsilon }_{D}\exp (-2/{\rho }_{\rm{qp}}U)$ with the density of states *ρ*_qp_ of intervalley coherent state quasiparticles, which is much suppressed compared to predictions from the Bardeen-Cooper-Schrieffer theory. The coherence length *ξ* we predict behaves according to $\xi \sim v/\sqrt{\mu {T}_{c}}$ with *v* being the velocity of Dirac cone. Applying our assumption to a microscopic model, our predictions align well with experimental data and effectively capture key measurable quantities such as the transition temperature *T*_*c*_ and the coherence length *ξ* `without parameter fine-tuning.

## Abstract (web summary)

1 The authors theoretically study superconductivity in rhombohedral tri-layer graphene. They propose 2 that the superconducting phase arises from the pairing of quasiparticles of the adjacent inter-valley 3 coherent state, instead of pairing between bare electrons.

## Introduction

Graphene heterostructures, in particular twisted bilayer graphene, have been observed experimentally to host various strongly correlated phases^1–10^. However, many of these heterostructures are difficult to realise in experiments due to structural instability^11,12^. Recently, breakthrough^13–15^ have been made in a more stable graphene-based system, namely ABC-stacked rhombohedral trilayer graphene^16,17^ (RTG), and have opened up exciting opportunities for studying strongly correlated phases of matter^18–21^. By adjusting the electron density and applying electric fields perpendicular to the material^22^, researchers can customise and investigate these states in RTG.

One particularly intriguing development is the discovery of superconductivity in RTG, which manifests in two distinct phases referred to as SC1 and SC2 in ref. ^13^. SC1 occurs within the flavour-symmetric phase and exhibits a maximum critical temperature of *T*_*c*1_ ~ 100mK and superconducting coherence length *ξ* ~ 200nm. There are significant discrepancies when applying the BCS theory. First, to align with experimental observations, the transition temperature *T*_*c*_ should obey ${T}_{c}\propto {\epsilon }_{D}\exp (-2/\rho U)$. It deviates from the antiadiabatic limit of Bardeen-Cooper-Schrieffer (BCS) theory applicable to low density of states^13,23^${T}_{c}\propto {\epsilon }_{D}\exp (-1/\rho U)$ where *ρ* is the density of states at the Fermi energy and *U* is the strength of the attractive interaction. Second, the coherence length reported in experiments is two orders of magnitude shorter than the value predicted by the BCS relation *ξ* = *ℏv*/*T*_*c*_, provided the large Fermi velocity *v* of the Dirac cone within graphene heterostructures. Several theories have been proposed to elucidate the electrons’ pairing mechanism behind SC1 in RTG, such as electron-phonon coupling^24,25^, Kohn-Luttinger-like mechanism^26–29^, direct coupling mediated by Coulomb interaction^30–32^, and pairing facilitated by the proximity to a correlated metal^33–35^. However, the influence of the neighbouring symmetry-breaking phase, which is likely an intervalley coherent (IVC) state, on the properties of the superconducting states remains unclear, in particular when it comes to explaining experimental measurements.

In this paper, we propose that SC1 observed in RTG is due to direct pairing between quasiparticles of the IVC state instead of the pairing between the bare electrons. We first clarify discrepancies from the experimental results^13^ both in the transition temperature and upper critical field, when we assume direct pairing between electrons. Instead, when in close vicinity with the phase transition from IVC to a flavour symmetric state, where the chemical potential cuts the band edge of the lower energy IVC quasiparticle band, there is an increase in density of state for the IVC quasiparticles. This favours direct pairing between IVC quasiparticles, leading to the IVC-SC phase, where the IVC order parameter remains intact. We then analytically study the unique properties of superconductivity near the band gap using a toy model that captures the key feature of the IVC state, namely, nearly massless Dirac fermions located at *K* and ${K}^{{\prime} }$ valleys. By parameterising the toy model accordingly to the RTG, we approximate the coherence length that is similar in value to what is observed in experiments, and explain a narrower window of superconductivity as opposed to the BCS theory^36^ on a highly dispersive band.

In closing the paper, we perform numerical calculations explicitly on the IVC states of RTG, calculating the transition temperature and upper critical field, which match well and capture key features of the experimental result. We also commented on the possible implication of the quantum metric near the phase boundary of the SC and the IVC state.

## Results

### Discrepancies in superconductivity of RTG

**Fig. 1:**
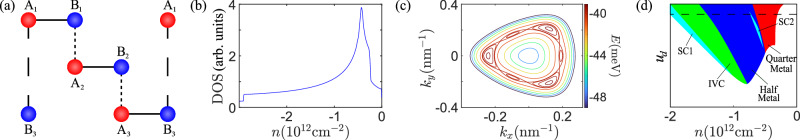
Lattice structure, dispersion spectrum, and phase diagram of RTG. **a** Schematic illustration of the lattice structure of RTG. Note that only *A*_1_ and *B*_3_ do not have direct interlayer hopping, and hence are of lower energy in comparison with other sites. As such, at the low charge carrier density, they act as active sites of the system. **b** The density of states (DOS) at the valence band of electrons in a flavour-symmetric phase. There is a jump at charge carrier density *n* ≈ − 2.9 × 10^12^cm^−2^, indicating a transition to an annulus Fermi surface from a complete one. Also, there is a Van Hove singularity when *n* ≈ − 0.5 × 10^12^cm^−2^, corresponding to Lifshitz transition to disconnected pockets. **c** Contour plot of the band structure at K valley. When the chemical potential varies, the Fermi surface topology is changed from disconnected pockets to annulus, and then to a completed surface, giving rise to the density of states in (**b**). **d** The phase diagram from the experiment in ref. ^13^. The flavour-symmetric state is left uncoloured. The SC1 occurs in between the flavour-symmetric state and the IVC state in a very narrow window of charge carrier densities. In obtaining (**b**) and (**c**), we set the potential difference between the outer layers of RTG as *u*_*d*_ = 34.5 meV, and the horizontal dashed line in (**d**) corresponds to *u*_*d*_ = 34.5meV.

**Fig. 2:**
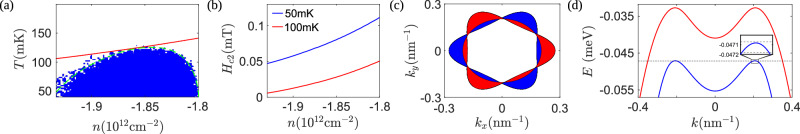
Predictions from the theory by assuming SC1 from flavour-symmetric phase. **a** The mean-field transition temperature (red curve) by assuming superconductivity from a flavour-symmetric phase. The blue region is extracted for SC1 from the experiment in ref. ^13^, and the green dashed line indicates the corresponding transition temperature. The predictions deviate from the experimental measurements in particular at the phase boundary with charge carrier density *n* = − 1.8 × 10^12^cm^−2^. **b** Out-of-plane upper critical field *H*_*c*2_ by assuming superconductivity from a flavour-symmetric phase. The two curves correspond to temperature *T* = 50 mK (Blue) and *T* = 100 mK (Red). The predictions are of two orders of magnitude smaller than the experimental results  ~ 10mT in ref. ^13^. Within the window of SC, the upper critical field is predicted to increase monotonically, as contrast to the experimental observations where *H*_*c*2_ reaches a maximum at *n* ≈ − 1.82 × 10^12^cm^−2^ and decreases to 0 at *n* ≈ − 1.8 × 10^12^cm^−2^. **c** Fermi surfaces corresponding to the Lifshitz transition from the annulus to isolated pockets, when *u*_*d*_ = 34.5meV at *n* = − 0.5 × 10^12^cm^−2^. The red and blue regions correspond to the valence band at K and ${K}^{{\prime} }$ valleys, respectively. The Fermi surfaces exhibit nearly perfect nesting, thus having strong instability under repulsive interaction. **d** Dispersion spectrum of the quasiparticles in Eq. (13) in the IVC state at different momenta $k=\sqrt{{k}_{x}^{2}+{k}_{y}^{2}}$. The dashed lines enclose the chemical potential of a very narrow regime where a superconducting state SC1 is observed in experiments, and the inset zooms in to show the regime.

For RTG, where the lattice structure is illustrated in Fig. 1(a), superconducting state SC1 is observed within the window of charge carrier density *n* ≈ − 1.93 × 10^12^cm^−2^ to  − 1.8 × 10^12^cm^−2^ when *u*_*d*_, which is the potential difference between the outer layers of RTG^16^, is approximately 34.5meV in experiment^13^. Despite the increase in density of states as illustrated in Fig. 1(b) due to the trigonal warping^17^ of the band structure as shown in Fig. 1(c), when we further decrease the carrier density, competing states, for example, the spin polarised state^14^, are more energy favourable, and thus a superconducting state is not observed, until SC2^13^, which is deep within the half-metal regime. A schematic for the phase diagram is illustrated in Fig. 1(d). Focusing on SC1, at *n* ≈ − 1.85 × 10^12^cm^−2^, corresponding to chemical potential of *μ* ≈ − 43.4meV, the transition temperature takes the maximal value of *T*_*c*_ ≈ 120mK. By fitting the maximum transition temperature at the specific charge carrier density, we estimate an attractive interaction strength of approximately *U* ≈ 4 eV using the self-consistency equation in a mean-field theory, as discussed in detail in sec. “Methods.” Specifically, the dispersion spectrum is extracted from the 6-band Hamiltonian for RTG^14,16^, detailed in Supplementary Material. Indeed, when considering electron pairing in the flavour-symmetric state and examining a wide range of charge carrier densities, a noticeable discrepancy emerges between the predicted trend of the transition temperature according to the mean-field theory and the experimental observations illustrated in Fig. 2(a). This discrepancy highlights the inadequacy of the consideration of pairing in the flavour-symmetric state in capturing the intricacies of the superconducting phase.

Significant contradiction from the experimental result can also be observed when we attempt to calculate the superconducting coherence length. At charge carrier density *n* ≈ − 1.85 × 10^12^cm^−2^, where the transition temperature is maximised, under BCS relation^36^, the coherence length can be approximated by *ξ*_0_ ~ 0.18*ℏv*/*k*_*B*_*T*_*c*_, where *v* is the typical velocity of the graphene heterostructure. We note that the chemical potential corresponding to the experimental regime is energetically far away from the $K({K}^{{\prime} })$ points, thus the interlayer tunnelling effect is negligible. Therefore, the typical velocity can be approximated by the Fermi velocity of massless graphene monolayer *ℏv* ≈ 6.6 × 10^−1^eVnm. Using *T*_*c*_ ≈ 120mK, we can approximate the coherence length as  ~ 10^4^nm, which is roughly two orders of magnitude larger than the coherence length (150 − 250nm) extracted from the experiment using the perpendicular upper critical field *H*_*c*2_ = *ϕ*_0_/2*πξ*^2^ where *ϕ*_0_ = *h*/2*e* is the superconducting quantum flux. If we additionally account for the low charge carrier density, from numerical calculations, at the base temperature ( ≈ 50mK) of the experiment, the coherence length lies between 1650 − 1800nm, which is still an order of magnitude larger than the experimental result. A plot of *H*_*c*2_ from numerical calculation is shown in Fig. 2(b).

These discrepancies from experiments to the BCS relation are clear indications that the superconductivity SC1 observed in RTG may not originate from the flavour-symmetric state, namely, the pairing of bare electrons. Instead, SC1 might emerge from the neighbouring competing state, which is referred to as the partially isospin polarised (PIP) state^13,14^ and most likely corresponds to the intervalley coherence state (IVC)^26,34^.

In the following, we propose that the Cooper pair in SC1, instead of originating from pairing between electrons, is from the direct pairing of quasiparticles of the IVC state, thus referred to as IVC-SC in the remainder of this paper. Therefore, SC1 is characterised by both the IVC order parameter and the superconducting order parameter simultaneously. In addition, we point out that the occurrence of IVC-SC in proximity to the phase boundary between the IVC state and the flavour-symmetric state is not merely coincidental but rather a key characteristic.

### Quasiparticle pairing in gapped Dirac cones

In this section, we will study the superconducting phase using a toy model. In the experimental regime, consideration of IVC quasiparticle (see sec. “Methods”) pairing allows realisation of superconductivity in the vicinity of the band gap, and the properties of the superconducting phase are distinct from the standard BCS theory. In our toy model, to mimic the IVC state in RTG, we use two Dirac cones, of Fermi velocity *v*, centred at the *K* and ${K}^{{\prime} }$ valley, respectively. By acting with an external potential, for example, a displacement field, we open up a mass gap *m* between the originally touching Dirac bands. The IVC phase can be effectively described in the continuum limit by a massive Dirac fermion Hamiltonian *H* = *H*_0_ + *V*_IVC_:

1 $${H}_{0}=\mathop{\sum }\limits_{s\tau }{\psi }_{\tau s}^{\dagger }({\bf{q}}){h}_{\tau }({\bf{q}}){\psi }_{\tau s}({\bf{q}}),$$

2 $${V}_{\rm{{IVC}}}=\mathop{\sum }\limits_{s}{\psi }_{+s}^{\dagger }({\bf{q}}){\Delta }_{\rm{{IVC}}}({\bf{q}}){\psi }_{-s}({\bf{q}})+h.c.$$

with the spinor ${\psi }_{\tau s}={[{a}_{\tau sA},{a}_{\tau sB}]}^{T}$ at two sublattices *λ* = *A*, *B* given the spin index *s* = *↑*, *↓* and the valley index *τ* = ± . In specific, *h*_*τ*_(**q**) describes a massive Dirac cone and is given by:

3 $${h}_{+}({\bf{q}})=v{\bf{q}}\cdot (\tau {\sigma }_{x}{\widehat{{\bf{e}}}}_{x}+{\sigma }_{y}{\widehat{{\bf{e}}}}_{y})+m{\sigma }_{z},$$

where *σ*_*x*,*y*,*z*_ correspond to the Pauli matrices defined in the sublattice basis. *V*_IVC_ introduces the IVC order parameter, which for simplicity, is assumed uniform and real (see “Methods” for details). Below, we will use the minimalistic toy model of a massive Dirac cone to obtain an analytical closed form, in order to qualitatively understand the phenomenology of band-edge superconductivity.

There are four quasiparticle bands in Eq. (1)–(2) given the spin index. In particular, we will focus on the quasiparticle band of the dispersion spectrum^34^$E({\bf{q}})=-\sqrt{{v}^{2}{q}^{2}+{m}^{2}}-{\Delta }_{{\rm{IVC}}}$, and consider only s-wave on-site effective attractive interaction:

4 $${H}_{\rm{{int}}}=-U\mathop{\sum }\limits_{s\lambda {\lambda }^{{\prime} }}\int \,{d}^{2}{\bf{r}}\,{a}_{+s\lambda }^{\dagger }({\bf{r}}){a}_{-\overline{s}{\lambda }^{{\prime} }}^{\dagger }({\bf{r}}){a}_{-\overline{s}{\lambda }^{{\prime} }}({\bf{r}}){a}_{+s\lambda }({\bf{r}}),$$

where *U* is the attractive interaction strength, and $\overline{s}$ indicates opposite spin. We do comment on the possibility of unconventional pairing^27,29–31^, given the interaction is non-local. We can analyse critical temperature and coherence length of the resulting SC through the both mean field theory and the Ginzburg-Landau theory (see sec. “Methods”).

We focus on the case where chemical potential ∣*μ*∣ − *m* < *ϵ*_*D*_, which is the relevant regime to experiments, where *ϵ*_*D*_ represents the Debye energy for the attractive interaction. For the regime where the chemical potential satisfies *β*_MF_(*m* − ∣*μ*∣) ≫ 1, thus away from the mass gap, the conventional contribution dominates, by a factor of  ~ *β*_MF_∣*μ*∣ ≫ 1. Thus, the system can be treated as conventional when away from the band edge, where we can retrieve results similar to the BCS limit, as detailed in sec. “Methods.”

We now consider the case where the chemical potential satisfies *β*_MF_∣*m* + *μ*∣ ≪ 1, thus in proximity to the band gap. The mean-field temperature is solved from the linearised gap equation in Eq. (20):

5 $${T}_{\rm{MF}}\propto {\epsilon }_{D}\exp \left(-\frac{2}{{\rho }_{\rm{qp}}{U}^{{\prime }}}\right),$$

where *ρ*_qp_ is the density of states of the quasiparticles at the Fermi energy. We have also defined $\frac{1}{{U}^{{\prime} }}=\frac{1}{U}-\frac{{\epsilon }_{D}{\mathcal{A}}}{4\pi {v}^{2}}$, with the correction term ${\epsilon }_{D}{\mathcal{A}}/4\pi {v}^{2}\ll 1$. Note that there is an extra factor of two in the exponential term in comparison with the BCS limit, as no states exist within the band gap, effectively halving the integral range over energy when calculating mean-field temperature self-consistently. This hints at superconductivity being more suppressed near the band edge, thus the range of doping where superconductivity can be observed, is expected to be narrower when compared with the prediction from BCS. The conventional coherence length in this regime is given by:

6 $${\xi }_{\rm{{con}}}\approx \frac{1}{4}\frac{v}{\sqrt{| \mu | {T}_{\rm{{MF}}}}}{\left(\frac{{T}_{\rm{{MF}}}-T}{{T}_{\rm{{MF}}}}\right)}^{-1/2},$$

which gives rise to the Ginzburg-Landau coherence length ${\xi }_{{\rm{GL}},{\rm{con}}}\approx \frac{1}{4}\frac{v}{\sqrt{| \mu | {T}_{MF}}}$. This relation deviates from a BCS relation *ξ*_BCS_ ≈ *v*_*F*_/*T*_MF_. As for the contribution from quantum metric, when the chemical potential is at the band edge (i.e. ∣*μ*∣ = *m*), we have:

7 $${\xi }_{\rm{{qm}}}\approx \frac{v}{4m}{\left(\frac{{T}_{\rm{{MF}}}-T}{{T}_{\rm{{MF}}}}\right)}^{-1/2}\sqrt{{\gamma }^{2}-1+2 \, {\rm{ln}}\left(\frac{{\beta }_{\rm{{MF}}}^{2}{m}^{2}}{\pi }\right)},$$

where *γ* is Euler’s constant, and we have the quantum metric Ginzburg-Landau coherence length ${\xi }_{\rm{{GL}}},{\rm{qm}} \approx \frac{v}{2m} \, {\rm{ln}} \, ({\beta }_{\rm{{MF}}}m)$. As such, we can conclude that the quantum metric contribution is smaller than, but can be comparable with the conventional coherence length, by a factor of $\sim 2\sqrt{{\rm{ln}} \, ({\beta }_{\rm{{MF}}}m)/{\beta }_{\rm{{MF}}}m} < 1$ at the valence band edge.

Another intriguing scenario arises when the chemical potential is in proximity to the edge of the band, but lies within the band gap. At zero temperature, one would not typically anticipate a superconducting phase since the ground state is an insulator. However, an SC phase can occur at a finite temperature due to a metallic phase induced by temperature fluctuations. Of course, the corresponding mean-field transition temperature gets suppressed. As the transitional temperature is small, such that *β*_MF_∣*μ* + *m*∣ ≫ 1, the conventional coherence length is proportional to:

8 $${\xi }_{GL,con}\propto \frac{v}{{T}_{{\rm{MF}}}}{e}^{-{\beta }_{\rm{{MF}}}| \mu+m| /2},$$

which goes to zero when we approach the phase boundary given a fixed chemical potential *μ*. On the contrary, the quantum metric coherence length diverges and is proportional to:

9 $${\xi }_{{\rm{GL}}},qm\propto \frac{v}{{T}_{{\rm{MF}}}}\sqrt{\frac{{\rm{ln}} \, ({\beta }_{\rm{{MF}}}m)}{{\beta }_{{\rm{MF}}}m}}.$$

Obviously, *ξ*_GL_, qm diverges when *T*_MF_ approaches zero. As such, without considering the effect of the quantum metric, we expect the Ginzburg-Landau coherence length to remain small as we approach the phase boundary. On the contrary, if the effect of the quantum metric is included, we would instead predict a divergent Ginzburg-Landau coherence length. As we will see, this case has potential implications for the interesting results in experimental measurements in RTG^13^.

### Prediction from the toy model on SC in RTG

With the toy model, we have obtained an analytical closed form that can invoke qualitative insight on the phenomenology of superconductivity observed in RTG. The IVC-SC phase is observed in the vicinity of the phase boundary in between the IVC and the flavour-symmetric state, at which the chemical potential cuts the band edge of the lower energy IVC band (coloured blue in Fig. 2d). This results in a discrete jump in DOS for the IVC quasiparticle, and leads to strong instability towards a flavour symmetric state. Since only quasiparticles from the edge of the lower energy IVC band majorly contribute to the formation of Cooper pairs, the lack of states within the direct band gap at zero temperature is well captured by the proposed toy model. As such, physical properties of IVC-SC, for example, coherence length, can be approximated using the result from the band edge superconductivity of massive Dirac fermions. Given the effective mass gap of *m* ≈ 34.5meV, with mean-field temperature *T*_MF_ = 120mK and graphene typical velocity *ℏv* = 6.6 × 10^−1^eVnm, the coherence length at base temperature  ≈ 50mK can be approximated using Eq. (6) to be 126nm, which is very close to the value (150-250nm) reported in the experiment^13^. Additionally, in the experiment^13^, it has been commented that the window of charge carrier density at which superconductivity can be observed is narrower than the prediction. We can understand the suppression directly from the transition temperature using Eq. (5) of the toy model, where the total number of charge carriers is halved within the energy cutoff *ϵ*_D_, which is consistent with the experiment^13^. These pieces of evidence strongly support the notion that SC1 emerges at the band edge, which can be achieved through the opening of a gap by other order parameters, such as the IVC order parameters. This justifies that the IVC-SC state we have proposed is indeed a strong candidate for SC1 observed in RTG.

We also comment on possible implications when the chemical potential is within the gap. Near the phase boundary, when the mean-field temperature approaches zero, our toy model predicts that the conventional contribution remains finite while the quantum metric contribution diverges. To verify our conclusions obtained using the toy model, we will conduct numerical calculations on the microscopic model, using Eqs. (20)-(28).

### Numerical calculations on the microscopic model

As a benchmark, we can calculate the property of the IVC-SC state in the microscopic model. We use a six-band microscopic model whose details are introduced in the supplementary material (see Supplemental Information: I. Details of 6-band Hamiltonian for RTG; II. Derivation of free energy; III. Quantum metric of massive Dirac cones; IV. Ginzburg-Landau Theory of Massive Dirac Cone; V. Discussion on pairing and time-reversal symmetry in IVC; VI. Details of fitting procedure for *U* and *Δ*_IVC_) and assume that ∣**Δ**_IVC_(**q**)∣ is independent of **q**. In general, one can solve for ∣**Δ**_IVC_∣ using a self-consistency equation, similar to the case of superconductivity. However, in our calculation, we will assume that in the superconducting phase, ∣**Δ**_IVC_∣ remains unchanged, and superconductivity occurs when in close vicinity with the band edge of *ϵ*_−_(**q**), thus a Cooper pair is formed between *ψ*_−,**q**_ states (15). Using the fact that at charge carrier density *n* ≈ − 1.8 × 10^12^cm^−2^ there is a sharp disappearance of superconductivity, we can deduce that ∣**Δ**_IVC_∣ ≈ 7meV, with details of fitting procedure discussed in the supplementary material (see Supplemental Information: I. Details of 6-band Hamiltonian for RTG; II. Derivation of free energy; III. Quantum metric of massive Dirac cones; IV. Ginzburg-Landau Theory of Massive Dirac Cone; V. Discussion on pairing and time-reversal symmetry in IVC; VI. Details of fitting procedure for *U* and Δ_IVC_).

**Fig. 3:**
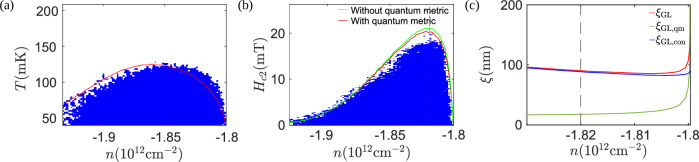
Predictions from the theory by assuming SC1 originates from the pairing of quasiparticles in the IVC phase. **a** the mean-field transition temperature, **b** upper critical field *H*_*c*2_ at *T* = 50 mK, and **c** the Ginzburg-Landau coherence lengths *ξ* at *n* ≈ − 1.82 × 10^12^cm^−2^. In (**a**), the SC1 occurs in the blue region, which is extracted from the experiment in ref. ^13^ with the transition temperature approximated by the boundary of the blue region. The mean-field temperature (red line) captures the key features of the experimental data, especially the disappearance of SC1 at charge carrier density *n* ≈ − 1.8 × 10^12^cm^−2^. In (**b**), the green curve corresponds to *H*_*c*2,_ only accounting for the conventional contribution, while the red curve additionally accounts for the quantum metric. The black dashed line indicates the chemical potential exactly at the Dirac point of the quasiparticle band in IVC. The blue region is extracted from ref. ^13^. Our theoretical predictions capture both the maximum in *H*_*c*2_ in close proximity with the band edge, and the sharp disappearance of superconductivity at *n* ≈ − 1.8 × 10^12^cm^−2^. In (**c**), the green and blue curves correspond to the quantum metric and conventional contributions, respectively, and the red curve is the overall Ginzburg-Landau coherence length *ξ*_GL_. The conventional contribution *ξ*_GL,con_ plays a dominating role, and the quantum metric contribution *ξ*_GL_, qm has a correctional effect in most regimes. However, near the phase transition from IVC-SC to IVC state for which the chemical potential lies between the band gap, the quantum metric contribution *ξ*_GL_, qm increases significantly, surpassing the conventional contribution *ξ*_GL,con_ due to the vanishingly low density of states of the IVC quasiparticles at the Fermi energy. It leads to a divergence of coherence length *ξ*_GL_ at the phase boundary, in contrast to a finite value for the conventional contribution at *n* ≈ − 1.8 × 10^12^cm^−2^. Here, the calculations are based on the microscopic model for RTG in SM^40^.

With the ∣**Δ**_IVC_∣ determined, we can calculate the effective attractive interaction strength, using Eq. (20), with the dispersion set to *ϵ*_−_(**q**). Numerically, the mean-field temperature is the highest when the chemical potential is around 22*μ*eV below the edge of the band. Setting the maximal mean-field temperature as 125mK, the effective attractive interaction strength *U* ≈ 1.2eV, which could have originated from the fluctuation of the IVC order parameter^34^. Similar to the flavour-symmetric state calculation, with the interaction strength determined, using Eq. (20), we can determine the mean-field temperature, as illustrated in Fig. 3(a). The mean-field temperature calculated from the IVC state, in general, agrees with the experimental result, both reaching a maximum at *n* ≈ − 1.85 × 10^12^cm^−2^, and then decreasing down to  ≈ 70mK at *n* ≈ − 1.93 × 10^12^cm^−2^, and is no longer superconducting for *n* > − 1.8 × 10^12^cm^−2^. We note that for *n* > − 1.82 × 10^12^cm^−2^, where the chemical potential no longer cuts the IVC band that is responsible for the formation of the Cooper pair, superconductivity can still emerge from the finite temperature effect. We also note that the mismatch in mean-field temperature could be due to contributions from other bands and a change in IVC order parameter as we tune the charge carrier density, which have been neglected in the numerical calculation.

We can calculate the coherence length using Eqs. (24)-(28), thus checking if the corresponding upper critical field *H*_*c*2_ matches with the experimental result. A plot of *H*_*c*2_ at base temperature 50mK, calculated from the IVC state dispersion, is shown in Fig. 3(b). We note that both the numerical calculations and the experimental measurement of *H*_*c*2_ have a maximum at density *n* ≈ − 1.82 × 10^12^cm^−2^. Our calculation can also well capture the drop in *H*_*c*2_ beyond the band edge, with the little discrepancy possibly originating from the mismatch in the mean-field temperature calculated numerically from the transition temperature near *n* ≈ − 1.82 × 10^12^cm^−2^. We also comment that the quantum metric could have a correctional effect on *H*_*c*2_, of approximately 5% at *n* ≈ − 1.82 × 10^12^cm^−2^. In fact, within the band gap, the variation in chemical potential is insignificant as illustrated in Fig. 2(d), and the drop in *H*_*c*2_ can mostly be accounted for by the decrease in mean-field temperature. In particular, the experimental temperature *T* = 50 mK approaches the mean-field transition temperature at *n* ≈ − 1.8 × 10^12^cm^−2^, resulting in *H*_*c*2_ = 0. However, the quantum metric effect can set in if one considers the zero-temperature coherence length, that is, the Ginzburg-Landau coherence length *ξ*_GL_, which is calculated in Fig. 3(c). In most regions of charge carrier densities, the conventional contribution *ξ*_GL,con_ (blue line in Fig. 3), dominates and will approach a finite value. By comparison, the quantum metric contribution *ξ*_GL_, qm will increase and finally diverge when the charge carrier density gets close to the phase boundary between the IVC-SC state and IVC state. This originates from the vanishingly low charge carrier density contributed by the relevant band at the Fermi energy when the chemical potential lies between the band gap and is very close to the band edge. Therefore, we can predict a divergent coherence length *ξ,* thus a vanishing upper critical field *H*_*c*2_ when the lower temperature can be realised for experimental measurements around *n* = − 1.8 × 10^12^cm^12^. To conclude, the numerical results on the microscopic model agree with qualitative insights previously obtained from the toy model.

We should also remark that in our model, the IVC-SC state has effectively extended the IVC order parameter into the flavour-symmetric phase, where the IVC state was originally not observed in higher temperature^13^. As such, contrary to the bare electron picture where IVC and SC orders are competing orders, in the SC-IVC state, where there exists an effective attractive interaction, thus Cooper pair formation directly between the IVC states, the SC order combining with the IVC order becomes more energy favourable than the flavour-symmetric phase.

## Discussion

We propose a scenario of quasiparticle pairing of IVC for the SC1 of RTG. Our proposal can well resolve the discrepancies for the transition temperature and coherence length reported in experiments. We construct a toy model of a graphene-like system, with Dirac points of Fermi velocity *v* at the *K* and ${K}^{{\prime} }$ valleys, and open up a mass gap *m* at the Dirac points while assuming an s-wave pairing. We determined the transition temperature of s-wave superconductivity near the band gap with the behaviour ${T}_{{\rm{MF}}}={\epsilon }_{D}\exp (-2/{\rho }_{{\rm{qp}}}U)$ where *ρ*_qp_ is the density of states of IVC quasiparticles in comparison to the antiadiabatic limit of the BCS theory on the low-density charge carriers. Therefore, when the chemical potential is near the mass gap, the transition temperature is more suppressed, and the conventional coherence length is no longer proportional to *v*/*T*_MF_, but instead $v/\sqrt{m{T}_{\rm{{MF}}}}$. We also note that the quantum metric could have a comparable contribution depending on the mean-field temperature, in comparison with the mass gap.

We want to comment on the role of the quantum metric, where the quantum metric only has a correction effect in the current experimental setup, on the superconducting coherence length observed in RTG. However, near the phase transition from SC-IVC to IVC state, the quantum metric contribution increases rapidly and diverges at the phase boundary, while the conventional contribution remains finite. This serves as smoking-gun evidence for future experiments to be performed at a lower temperature, allowing distinction between conventional and quantum metric effect for *H*_*c*2_.

In a recent experiment^37^ of Bernal bilayer graphene with spin-orbit coupling, there is substantial evidence that superconductivity is prohibited when no neighbouring/coinciding PIP is observed, further supporting our claims that quasiparticles are essential for superconductivity near/within flavour-symmetry-breaking phases. We believe our quasiparticle pairing picture can, in general, provide a phenomenological description for interaction-driven correlated phases, when there exists a neighbouring/overlapping flavour-symmetry-breaking phase, and, for example, possibly explain the discrepancy in the in-plane upper critical field between electron-doped and hole-doped superconductivity, observed in ref. ^37^, which we leave to future work.

## Methods

### Mean-field theory of the Intervalley coherence state

As discussed in Fig. 1(b), the density of states of electrons diverges at the charge carrier density *n* ≈ − 0.5 × 10^12^cm^−2^. At this doping level, the K and ${K}^{{\prime} }$ valley Fermi surfaces are almost perfectly nested together as illustrated in Fig. 2(c). Assuming a repulsive interaction between K and ${K}^{{\prime} }$ valley, the intervalley nesting would energetically favour the IVC state, for which a gap is opened at the nested portion of the Fermi surfaces. To further study the IVC state, we can define the mean-field Hamiltonian:

10 $${H}_{MF}=\mathop{\sum }\limits_{\tau,{\tau }^{{\prime} },{\bf{q}}}{\psi }_{\tau,{\bf{q}}}^{\dagger }{h}_{\tau,{\tau }^{{\prime} }}({\bf{q}}){\psi }_{{\tau }^{{\prime} },{\bf{q}}},$$

with the Hamiltonian matrix as

11 $${h}_{\tau,{\tau }^{{\prime} }}({\bf{q}})={\epsilon }_{s}({\bf{q}}){I}_{2}+{\boldsymbol{\Delta }}({\bf{q}})\cdot {\boldsymbol{\tau }}.$$

The **Δ**(**q**) is related to the IVC order parameter,

12 $${\boldsymbol{\Delta }}({\bf{q}})\,=(| {{\boldsymbol{\Delta }}}_{{\rm{IVC}}}({\bf{q}})| \cos ({\phi }_{{\bf{q}}}),| {{\boldsymbol{\Delta }}}_{\rm{{IVC}}}({\bf{q}})| \sin ({\phi }_{{\bf{q}}}),{\epsilon }_{a}({\bf{q}})).$$

where *I*_2_ is the 2 × 2 identity matrix and ***τ*** = (*τ*_1_, *τ*_2_, *τ*_3_) are Pauli matrices. In Eq. (12), ${\epsilon }_{s}({\bf{q}})=-\mu+\frac{1}{2}[{\epsilon }_{K}({\bf{q}})+{\epsilon }_{{K}^{{\prime} }}({\bf{q}})]$ and ${\epsilon }_{a}({\bf{q}})=\frac{1}{2}[{\epsilon }_{K}({\bf{q}})-{\epsilon }_{{K}^{{\prime} }}({\bf{q}})]$ are the symmetric and antisymmetric parts of the flavour-symmetric state dispersion, respectively, and ${\Delta }_{\rm{{IVC}}}({\bf{q}})=| {{\boldsymbol{\Delta }}}_{{\rm{IVC}}}({\bf{q}})| \exp (i{\phi }_{{\bf{q}}})$ is the order parameter for the IVC state. Note that the IVC order parameter has a phase that can be well-matched with the phase of the intervalley form factor^34^. The dispersion spectrum of the IVC state is given by:

13 $${\epsilon }_{\pm }({\bf{q}})\approx {\epsilon }_{s}({\bf{q}})\pm | {\boldsymbol{\Delta }}({\bf{q}})| .$$

The dispersion spectrum for IVC states is shown in Fig. 2(d). The IVC states defined in the valley basis, correspond to two quasiparticle bands, with Bloch states:

14 $${\psi }_{+,{\bf{q}}}=\frac{1}{\sqrt{2| {\boldsymbol{\Delta }}({\bf{q}})| }}\left(\begin{array}{l}{e}^{-i{\phi }_{{\bf{q}}}}\sqrt{| {\boldsymbol{\Delta }}({\bf{q}})| + {\epsilon }_{a}({\bf{q}})}\\ \sqrt{| {\boldsymbol{\Delta }}({\bf{q}})| -{\epsilon }_{a}({\bf{q}})}\end{array}\right),$$

15 $${\psi }_{-,{\bf{q}}}=\frac{1}{\sqrt{2| {\boldsymbol{\Delta }}({\bf{q}})| }}\left(\begin{array}{l}-{e}^{-i{\phi }_{{\bf{q}}}}\sqrt{| {\boldsymbol{\Delta }}({\bf{q}})| -{\epsilon }_{a}({\bf{q}})}\\ \sqrt{| {\boldsymbol{\Delta }}({\bf{q}})|+{\epsilon }_{a}({\bf{q}})}\end{array}\right).$$

Note that time reversal symmetry remains intact after the formation of the IVC state (see Supplemental Information: I. Details of 6-band Hamiltonian for RTG; II. Derivation of free energy; III. Quantum metric of massive Dirac cones; IV. Ginzburg-Landau Theory of Massive Dirac Cone; V. Discussion on pairing and time-reversal symmetry in IVC; VI. Details of fitting procedure for *U* and Δ_IVC_), which is crucial for the existence of superconductivity in our proposal by assuming an s-wave pairing. We should also retrieve an effective attractive interaction to enable the formation of Cooper pairs. By adopting the argument in ref. ^26^, involving the introduction of a weaker anti-ferromagnetic Hund’s coupling compared to the repulsive intervalley interaction, it becomes possible to achieve an effective attractive intervalley interaction. This ultimately results in the emergence of a spin-singlet superconducting phase. We comment that the interaction between quasiparticle could also have originated from the fluctuations of the quasiparticle order parameter, as illustrated in ref. ^34^. As shown in Fig. 2(d), the density of states of the quasiparticles peaks at the edge of the valence band (the blue line), which allows us to introduce a toy model of massive Dirac cones with the chemical potential close to the band edge.

### Ginzburg-Landau theory on toy models

We mimic the IVC-SC state with a simplified toy model. In our toy model, similar to RTG, we have two Dirac cones, of Fermi velocity *v*, centred at the *K* and ${K}^{{\prime} }$ valley respectively. By acting with an external potential, for example, a displacement field, we open up a mass gap *m* between the originally. The system can be described effectively in the continuum limit by introducing an IVC order to massive Dirac fermion Hamiltonian at *K* and ${K}^{{\prime} }$ valleys. At mean-field level, the Hamiltonian ${H}_{{\rm{IVC}}}=\int \frac{{d}^{2}q}{{(2\pi )}^{2}}{\Psi }_{s}^{\dagger }({\bf{q}}){{\mathcal{H}}}_{s}({\bf{q}}){\Psi }_{s}({\bf{q}})$ for spin *s* = *↑*, *↓* is given by assuming degeneracy for the two spin sectors:

16 $${{\mathcal{H}}}_{s}=\left(\begin{array}{ll}{h}_{+}({\bf{q}}) & \Delta ({\bf{q}})\\ {\Delta }^{\dagger }({\bf{q}}) & {h}_{-}({\bf{q}})\end{array}\right)$$

where $\Delta ({\bf{q}})={\Delta }_{\rm{{IVC}}}\,{\rm{diag}}([{e}^{-i{\phi }_{q}},-{e}^{-i{\phi }_{q}}])$ with IVC order parameter Δ_IVC_ and *qe*^*iϕ*^ = *q*_*x*_ + *iq*_*y*_. We use the valley-sublattice basis ${\Psi }_{s}={[{a}_{+sA},{a}_{+sB},{a}_{-sA},{a}_{-sB}]}^{T}$ with *h*_*τ*_(**q**) being the massive Dirac fermion Hamiltonian, of valley indices *τ* = ± , and **q** the momentum difference to the centre of the valley. In specific, *h*_*τ*_(**q**) is given by:

17 $${h}_{\tau }({\bf{q}})=v{\bf{q}}\cdot (\tau {\sigma }_{x}{\widehat{{\bf{e}}}}_{x}+{\sigma }_{y}{\widehat{{\bf{e}}}}_{y})+m{\sigma }_{z},$$

where *σ*_*x*,*y*,*z*_ correspond to the Pauli matrix defined in the orbital basis, and $\overline{\tau }$ refers to the opposite valley. In particular, we will focus on the quasiparticle band of dispersion spectrum $E({\bf{q}})=\sqrt{{v}^{2}{q}^{2}+{m}^{2}}\pm {\Delta }_{{\rm{IVC}}}$, assuming Δ_IVC_ ≪ *m* and consider only on-site effective attractive interaction between the time-reversal copy of quasiparticles. This can be realised as intervalley interaction for a graphene heterostructure:

18 $${H}_{\rm{{int}}}=-U\mathop{\sum }\limits_{s\lambda {\lambda }^{{\prime} }}\int {d}^{2}{\bf{r}}\,{a}_{+s\lambda }^{\dagger }({\bf{r}}){a}_{-\overline{s}{\lambda }^{{\prime} }}^{\dagger }({\bf{r}}){a}_{-\overline{s}{\lambda }^{{\prime} }}({\bf{r}}){a}_{+s\lambda }({\bf{r}}),$$

where *U* is the attractive interaction strength and *a*_*τsλ*_(**r**) is the quasiparticle annihilation operator of the valley *τ*, spin *s* and sublattice index *λ*. Due to the large band gap separating the conduction and valence band, we will focus on the contributions of the valence bands of the toy model, which can be realised via a projection by involving the Bloch waves $| {u}_{\tau }({\bf{q}})\rangle$ of the valence bands^38,39^,

19 $${a}_{\tau s\lambda }({\bf{r}})\to \int \frac{{d}^{2}{\bf{q}}}{{(2\pi )}^{2}}{e}^{i{\bf{q}}\cdot {\bf{r}}}{u}_{\tau \lambda }^{*}({\bf{q}}){c}_{s}({\bf{q}}),$$

where *c*_*s*_(**q**) is the quasiparticle annihilation operator of momentum **q** for the valence band. The time reversal symmetry is intact for an IVC quasiparticle band, ${u}_{\tau \lambda }({\bf{q}})={u}_{\overline{\tau }\lambda }^{*}(-{\bf{q}})$, and thus the effective attractive interaction is the coupling between IVC quasiparticles of opposite momentum. Via the Hubbard-Stratonovich transformation^40–43^, we can then introduce a bosonic field Δ(**r**) = ∑_*s*_*c*_*↑*_(**r**)*c*_*↓*_(**r**) that corresponds to Cooper pairs for the projected Hamiltonian. Using the path integral formalism, we can evaluate the free energy of the system, which can be decomposed into two components: the mean-field solution and the fluctuations. This decomposition is achieved by expanding the free energy around its extremum. Further details of the calculation can be found in the Supplementary Information (see Supplemental Information: I. Details of 6-band Hamiltonian for RTG; II. Derivation of free energy; III. Quantum metric of massive Dirac cones; IV. Ginzburg-Landau Theory of Massive Dirac Cone; V. Discussion on pairing and time-reversal symmetry in IVC; VI. Details of fitting procedure for *U* and Δ_IVC_). Focusing on the case around the transition region where the mean-field value of the bosonic field Δ_0_ vanishes, minimising the mean-field solution of the free energy provides us with a self-consistency condition for the mean-field temperature, at which transition to the superconducting state initiates:

20 $$1=U\int \frac{{d}^{2}{\bf{q}}}{{(2\pi )}^{2}}\frac{1}{2\varepsilon ({\bf{q}})} \, \tanh \frac{{\beta }_{\rm{{MF}}}\varepsilon ({\bf{q}})}{2},$$

where *T*_MF_ = 1/*β*_MF_ is the mean-field temperature, and for convenience we have defined *ε*(**q**) = *ϵ*_0_(**q**) − *μ*. The fluctuation of the free energy, in particular the quadratic term *F*_2_, can be related to the fluctuation of the order parameter *δ*Δ(**k**). After integrating out the fermionic fields *F*_2_ takes the form:

21 $${F}_{2}=\int \frac{{d}^{2}{\bf{k}}}{{(2\pi )}^{2}}| \delta \Delta ({\bf{k}}){| }^{2}[U-{U}^{2}\chi ({\bf{k}})],$$

where *χ*(**k**) is the four-point coherence function,

22 $$\chi ({\bf{k}})=\int \frac{{d}^{2}{\bf{q}}}{{(2\pi )}^{2}}| \Gamma ({\bf{q}},{\bf{k}}){| }^{2}\\ \times \frac{\tanh [\frac{\beta }{2} \, \varepsilon ({\bf{q}}+\frac{{\bf{k}}}{2})]+\tanh [\frac{\beta }{2}\varepsilon ({\bf{q}}-\frac{{\bf{k}}}{2})]}{2 \, [\varepsilon ({\bf{q}}+\frac{{\bf{k}}}{2})+\varepsilon \, ({\bf{q}}-\frac{{\bf{k}}}{2})]}.$$

And we have defined the form factor $| \Gamma ({\bf{q}},{\bf{k}}){| }^{2}={| {u}_{c ,+}^{T}({\bf{q}}+\frac{{\bf{k}}}{2}) \, {u}_{c,-}(-{\bf{q}}+\frac{{\bf{k}}}{2})| }^{2}$, which was introduced upon performing projection (19) from a multiband system onto a single relevant band. Given time reversal invariant of the Bloch states ${u}_{c,-}^{*}(-{\bf{q}})={u}_{c ,+}({\bf{q}})\equiv u({\bf{q}})$, we can expand the form factor around **k** = 0 as^38^:

23 $$| \Gamma ({\bf{q}},{\bf{k}}){| }^{2}=1-\mathop{\sum }\limits_{i,j}{k}_{i}{k}_{j}{g}_{ij}({\bf{q}}),$$

where ${g}_{ij}({\bf{q}})=\frac{1}{2}{\rm{Tr}}[{\partial }_{i}P({\bf{q}}){\partial }_{j}P({\bf{q}})]$ is the quantum metric, and $P({\bf{q}})=| u({\bf{q}})\rangle \langle u({\bf{q}})|$ is the projection matrix for the Bloch state. Similarly, we can expand the four-point coherence function *χ*(**k**) around **k** = 0, with the assumption that the valence band is rotational invariant as:

24 $$\chi ({\bf{k}})={\chi }_{0}-({\chi }_{2,{\rm{con}}}+{\chi }_{2,{\rm{qm}}}) \, {k}^{2}+{\mathcal{O}}({k}^{4}),$$

where *χ*_2,con_ is the contribution due to dispersion of the band, and *χ*_2,qm_ is due to the form factor from the quantum metric. Recalling the definition of *F*_2_(21), we can construct an effective Lagrangian that governs the fluctuation of the order parameter *δ*Δ(**k**) formed as ${F}_{2}=\int \frac{{d}^{2}{\bf{k}}}{{(2\pi )}^{2}}{\mathcal{L}}[\delta \Delta ]$:

25 $${\mathcal{L}}[\delta \Delta ]=\frac{1}{2{m}^{*}}| \nabla \delta \Delta {| }^{2}+\alpha | \delta \Delta {| }^{2}+{\mathcal{O}}(| \delta \Delta {| }^{4}),$$

with the coefficients

26 $$\frac{1}{2{m}^{*}}={U}^{2}({\chi }_{2,{\rm{con}}}+{\chi }_{2,{\rm{qm}}}),$$

27 $$\alpha=U-{U}^{2}{\chi }_{0}.$$

From the effective Lagrangian, we can determine the general form of the superconducting coherence length, as motivated by the Ginzburg-Landau theory:

28 $$\xi=\sqrt{\frac{{U}^{2}({\chi }_{2,{\rm{con}}}+{\chi }_{2,{\rm{qm}}})}{| U-{U}^{2}{\chi }_{0}| }}=\sqrt{{\xi }_{\rm{{con}}}^{2}+{\xi }_{\rm{{qm}}}^{2}},$$

where the overall coherence length is governed by both the conventional contribution and the quantum metric^38,39^. The conventional contribution *ξ*_con_ depends on the band dispersion with a similar form as a conventional s-wave superconductor, and it vanishes in the flat-band limit. The quantum metric part *ξ*_qm_ exclusively appears as a multi-band effect, and it is determined by the quantum metric *g*_*ij*_. The coherence length *ξ* will be scaled as $\xi={(\frac{{T}_{{\rm{MF}}}-T}{{T}_{{\rm{MF}}}})}^{-1/2}{\xi }_{\rm{{GL}}}$ since the Ginzburg-Landau theory is developed around *T* → *T*_MF_, while we extract the Ginzburg-Landau coherence length *ξ*_GL_ as in the standard way. We comment that Eqs. (19)-(28) are applicable to any microscopic model, such as an IVC state in RTG, which hosts two valleys with dispersion *ϵ*_+_(**q**) = *ϵ*_–_(**-q**), and has local attractive interaction between time-reversal copies of the same quasiparticle, which can be realised with onsite intervalley interaction given by Eq. (18). In the sec. “Result,” we have studied the case that is relevant to the IVC-SC state, for which the mass gap *m* ≫ *T*_MF_, in particular, when the chemical potential is near the band gap. Details of the analytical calculation and a brief discussion of the *m* ≪ *T*_MF_ regime are included in the supplementary material (see Supplemental Information: I. Details of 6-band Hamiltonian for RTG; II. Derivation of free energy; III. Quantum metric of massive Dirac cones; IV. Ginzburg-Landau Theory of Massive Dirac Cone; V. Discussion on pairing and time-reversal symmetry in IVC; VI. Details of fitting procedure for *U* and Δ_IVC_).

Below we will illustrate with the case of *m* ≫ *T*_MF_, when the chemical potential satisfies *β*_MF_(*μ* − *m*) ≫ 1, thus away from the mass gap. The mean-field temperature can be solved self-consistently by Eq. (20):

29 $${T}_{\rm{MF}} \propto \sqrt{{\rho }_{qp}{\epsilon }_{D}} \exp \left(-\frac{1}{{\rho }_{qp}{U}^{{\prime} }}\right),$$

note that as *μ* → *ϵ*_*D*_, the BCS relationship for mean-field temperature ${T}_{\rm{MF}}\propto {\epsilon }_{D}\exp (-1/{\rho }_{\rm{qp}}U)$ can be recovered. We can also derive the conventional coherence length *ξ*_con_, which is proportional to *v*/*T*_MF_, similar to the BCS limit:

30 $${\xi }_{\rm{{con}}}\approx \frac{v}{{T}_{\rm{{MF}}}}{\left(\frac{{T}_{\rm{{MF}}}-T}{{T}_{\rm{{MF}}}}\right)}^{-1/2}\sqrt{\frac{7\zeta (3)}{32{\pi }^{2}}},$$

where *ζ* is the Riemann zeta function and the ${\xi }_{{\rm{GL}},{\rm{con}}}\propto \frac{v}{{T}_{\rm{MF}}}$. As for the quantum metric contribution, it can be approximated by:

31 $${\xi }_{\rm{{qm}}}\approx \frac{v}{4\mu }{\left(\frac{{T}_{\rm{{MF}}}-T}{{T}_{\rm{{MF}}}}\right)}^{-1/2}\sqrt{{\rm{ln}} \, \left(\frac{{\mu }^{3}}{m{T}_{\rm{{MF}}}^{2}}\right)},$$

where the factor $\sqrt{{\rm{ln}}({\mu }^{3}/m{T}_{\rm{{MF}}}^{2})}$ is in order of unity. As such, the conventional contribution dominates by a factor of  ~ *β*_MF_*μ* ≫ 1. Thus, the quantum metric effect is negligible, and the system can be treated as conventional when away from the band edge.

## Supplementary information

Supplementary Information

Transparent Peer Review file

## Data Availability

This is a theoretical study, and no experimental datasets were generated or analysed. All results can be reproduced from the equations and parameters provided in the main text and Supplementary Information.
