# Supplementary material for: Superconductivity from quasiparticle pairing of intervalley coherent state in rhombohedral trilayer graphene
Source: Nat Commun. 2026 May 28;17:6939. doi: 10.1038/s41467-026-72135-y (PMC13389250; doi:10.1038/s41467-026-72135-y)
Supplement: Supplementary file 1 — Supplementary Information [file 41467_2026_72135_MOESM1_ESM.pdf]

Supplementary Information of "Superconductivity from Quasiparticle Pairing of Intervalley Coherent State in Rhombohedral Trilayer Graphene"

Chun Wang Chau, Shuai A. Chen, and K. T. Law
Department of Physics, Hong Kong University of Science and Technology, Clear Water Bay, Hong Kong, China

CONTENTS

I. Details of 6-band Hamiltonian for RTG 1

II. Derivation of free energy 2

III. Quantum metric of massive Dirac cones 6

IV. Ginzburg-Landau Theory of Massive Dirac Cone 8
A. Case 1: $m \gg T_{\mathrm{MF}}$ 8
1. Beyond band edge 13
B. Case 2: $T_{\mathrm{MF}} \gg m$ 14

V. Discussion on pairing and time-reversal symmetry in IVC 15

VI. Details of fitting procedure for U and Δ_{IVC} 17

Supplementary References 17

References 17

I. DETAILS OF 6-BAND HAMILTONIAN FOR RTG

Following the definition of the sub-lattice A and B in the single layer of graphene, we can denote the structure of RTG by stating which pairs of sites coincide from the top view:

$$B_1 \leftrightarrow A_2,\ B_2 \leftrightarrow A_3,\ B_3 \leftrightarrow A_1 \tag{1}$$

As such we can classify our hopping [1] ($S_i \in \{A_i, B_i\}$):

$$\begin{aligned}
\gamma_0 :& A_i \leftrightarrow B_i, \quad i = 1,2,3 \\
\gamma_1 :& B_i \leftrightarrow A_{i+1}, \quad i = 1,2 \\
\gamma_2 :& A_1 \leftrightarrow B_3 \\
\gamma_3 :& A_i \leftrightarrow B_{i+1}, \quad i = 1,2 \\
\gamma_4 :& S_i \leftrightarrow S_{i+1}, \quad i = 1,2 \\
\gamma_5 :& B_1 \leftrightarrow A_3 \\
\gamma_6 :& S_1 \leftrightarrow S_3
\end{aligned}$$

Due to direct interlayer hopping γ_1 between B_i and A_{i+1}, sites not involved, namely A_1 and B_3, would have lower energy. We denote the energy difference of $A_1(B_3)$ from $B_1(A_3)$ as $\delta < 0$. Note that these hopping are listed in decreasing magnitude, with γ_3 and γ_4 having similar order. γ_5 and γ_6 having similar order as well, but should be much weaker in comparison.

We should also note the massless Dirac fermion contribution near the valleys, for a single layer graphene takes the form:

$$H = v_0 \begin{pmatrix} 0 & \pi^\dagger \\ \pi & 0 \end{pmatrix}, \tag{2}$$

TABLE I. Tight-binding Parameters for Rhombohedral Trilayer Graphene. Retrieved from [2].

Hopping parameters	Values (eV)
δ	-0.00105
γ_0	3.1
γ_1	0.38
γ_2	-0.015
γ_3	-0.29
γ_4	-0.141
u_a	-0.0023

where we have defined $\pi = \tau p_x + ip_y$, with $\tau = +(-)$ correspond to the $K(K')$ valley. With the single layer picture and list of possible hopping in mind, we can construct our basis as $(A_1, B_3, B_1, A_2, B_2, A_3)$ for convenience:

$$\mathcal{H} = \begin{pmatrix} u_1+\delta & \gamma_2/2 & v_0\pi^\dagger & v_4\pi^\dagger & v_3\pi & v_6\pi \\ \gamma_2/2 & u_3+\delta & v_6\pi^\dagger & v_3\pi^\dagger & v_4\pi & v_0\pi \\ v_0\pi & v_6\pi & u_1 & \gamma_1 & v_4\pi^\dagger & v_5\pi^\dagger \\ v_4\pi & v_3\pi & \gamma_1 & u_2 & v_0\pi^\dagger & v_4\pi^\dagger \\ v_3\pi^\dagger & v_4\pi^\dagger & v_4\pi & v_0\pi & u_2 & \gamma_1 \\ v_6\pi^\dagger & v_0\pi^\dagger & v_5\pi & v_4\pi & \gamma_1 & u_3 \end{pmatrix}, \tag{3}$$

where $v_i = \sqrt{3}a\gamma_i/2\hbar$, as motivated by single layer graphene. ($a = 0.246$nm)

Note that for convenience, we will take $v_i = \sqrt{3}a\gamma_i/2$. And define $\mathbf{q}$ as the wavevector instead of using momentum $\mathbf{p}$ to compensate for the factor of $\hbar$. The low-energy effective Hamiltonian is:

$$H_{\text{eff}} = H_{\text{ch}} + H_{\text{s}} + H_{\text{tr}} + H_{\text{gap}} + H'_{\text{s}}, \tag{4}$$

$$H_{\text{ch}} = \frac{(v_0 q)^3}{\gamma_1^2}[\cos(3\varphi_{\mathbf{q}})\sigma_x + \sin(3\varphi_{\mathbf{q}})\sigma_y], \tag{5}$$

$$H_{\text{s}} = \left(\delta - \frac{2v_0 v_4 q^2}{\gamma_1}\right)\sigma_0, \tag{6}$$

$$H_{\text{tr}} = \left(\frac{\gamma_2}{2} - \frac{2v_0 v_3 q^2}{\gamma_1}\right)\sigma_x, \tag{7}$$

$$H_{\text{gap}} = u_d\left[1 - \left(\frac{v_0 q}{\gamma_1}\right)^2\right]\sigma_z, \tag{8}$$

$$H'_{\text{s}} = \frac{u_a}{3}\left[1 - 3\left(\frac{v_0 q}{\gamma_1}\right)^2\right]\sigma_0, \tag{9}$$

where we have defined $\tan\varphi_{\mathbf{q}} = q_y/q_x$, $u_d = (u_1 - u_3)/2$ and $u_a = (u_1 + u_3)/2 - u_2$. In our scope of discussion, the contribution from γ_5 and γ_6 are assumed to be small thus neglected.

We will use parameters fitted in [2], with the parameters listed in Table I. The parameter u_d, which is the energy difference between each layer, can be induced via an external displacement field [3]: $u_d = \frac{ez}{\epsilon_r}D$, where $z = 0.33$nm is the interlayer distance, and $\epsilon_r = 4.4$ is the dielectric constant hexagonal boron nitride. In our study, we will focus on superconductivity of the valence band when $D = 0.46$V/nm, which corresponds to $u_d = 34.5$meV.

II. DERIVATION OF FREE ENERGY

We mimic the IVC-SC state by a simplified toy model. In our toy model, similar to RTG, we have two Dirac cones, of Fermi velocity v, centered at the K and K' valley respectively. By acting with an external potential, for example a displacement field, we open up a mass gap m between the originally touching Dirac bands. The system can be described effectively in the continuum limit by introducing intervalley density-density interaction to massive Dirac

fermion Hamiltonian at K and K' valleys, which at mean-field level for spin $s=\uparrow,\downarrow$ is given by:

$$\mathcal{H}_s = \begin{pmatrix} h_+(\mathbf{q}) & \Delta(\mathbf{q}) \\ \Delta^*(\mathbf{q}) & h_-(\mathbf{q}) \end{pmatrix}$$
$$\Delta(\mathbf{q}) = \sum_n \Delta_{\rm IVC}\tilde{u}_{n,-}(\mathbf{q})\tilde{u}^\dagger_{n,+}(\mathbf{q}) \tag{10}$$

where we have assumed degeneracy for the two spin sectors, with IVC order parameter Δ. Here we use the valley-sublattice basis $\Psi_s = [a_{+sA}, a_{+sB}, a_{-sA}, a_{-sB}]^T$. With $h_\tau(\mathbf{q})$ being the massive Dirac fermion Hamiltonian, of valley indices $\tau=\pm$, $\mathbf{q}$ the momentum difference to the center of the valley, and $\tilde{u}_{n,\tau}(\mathbf{q})$ being the Bloch vector of Hamiltonian $h_\tau(\mathbf{q})$ of band index n. In specific, $h_\tau(\mathbf{q})$ is given by:

$$h_\tau(\mathbf{q}) = v\mathbf{q}\cdot(\tau\sigma_x\hat{\mathbf{e}}_x + \sigma_y\hat{\mathbf{e}}_y) + m\sigma_z, \tag{11}$$

where v is the fermi velocity before gap-opening, $\tau=+/-$ correspond to K and K' valley respectively, m is the gap size, and $\mathbf{q}$ is the momentum difference with respect to the center of the valley, and σ_i Pauli matrices that act on the sublattice basis. Corresponding Bloch states in sublattice basis of $\mathcal{H}_s(\mathbf{q})$ are given by:

$$u_{c,\eta,\tau}(\mathbf{q}) = \frac{1}{\sqrt{2\epsilon_0(\mathbf{q})}}\begin{pmatrix} e^{-i\tau\phi/2}\sqrt{\epsilon_0(\mathbf{q})+m} \\ \tau e^{i\tau\phi/2}\sqrt{\epsilon_0(\mathbf{q})-m}\end{pmatrix}\otimes\frac{1}{\sqrt{2}}\begin{pmatrix}\eta \\ 1\end{pmatrix} \qquad E = \epsilon_0(\mathbf{q}) + \eta\Delta_{\rm IVC}, \tag{12}$$
$$u_{v,\eta,\tau}(\mathbf{q}) = \frac{1}{\sqrt{2\epsilon_0(\mathbf{q})}}\begin{pmatrix} -e^{-i\tau\phi/2}\sqrt{\epsilon_0(\mathbf{q})-m} \\ \tau e^{i\tau\phi/2}\sqrt{\epsilon_0(\mathbf{q})+m}\end{pmatrix}\otimes\frac{1}{\sqrt{2}}\begin{pmatrix}\eta \\ 1\end{pmatrix} \qquad E = -\epsilon_0(\mathbf{q}) + \eta\Delta_{\rm IVC}, \tag{13}$$

where $\epsilon_0(\mathbf{q})$ is given by $\sqrt{v^2q^2+m^2}$, index c (v) denotes the conduction (valence) band, $\eta=\pm$ denote the pair of quasiparticle bands, and have rewritten our momentum in polar coordinate as $qe^{i\phi_q} = q_x + iq_y$. Note the explicit assumption that the Hamiltonian is the same for spin up and down fermions. We can also write down the exact form of the IVC order parameter:

$$\Delta(\mathbf{q}) = \Delta_{\rm IVC}\begin{pmatrix} e^{i\phi_q} & 0 \\ 0 & -e^{-i\phi_q}\end{pmatrix} \tag{14}$$

To induce superconductivity in the system, we introduce an effective attractive interaction between quasiparticles:

$$H_{\rm int} = -U\sum_{s\lambda\lambda'}\int d^2\mathbf{r}\; a^\dagger_{+s\lambda}(\mathbf{r})a^\dagger_{-\bar{s}\lambda'}(\mathbf{r})a_{-\bar{s}\lambda'}(\mathbf{r})a_{+s\lambda}(\mathbf{r}), \tag{15}$$

where $a_{\tau s\lambda}(\mathbf{r})$ is the fermionic annihilation operator of states in valley of momentum $\tau\mathbf{K}$ of spin $s=\uparrow,\downarrow$ and sublattice $\lambda = A,\ B$ at position $\mathbf{r}$. Below to simplify the calculation we choose $s=\uparrow$, which has no effect in the overall result, and we will not write down the spin index explicitly. As such we can write down a partition function involving only fermionic fields a:

$$Z = \int D[a,\bar{a}]e^{-\int_0^\beta d\tau\int d\mathbf{r}\mathcal{L}_a[a,\bar{a}]}, \tag{16}$$
$$\mathcal{L}_a[a,\bar{a}] = \mathcal{L}_{\rm Dirac}[a,\bar{a}] + (\partial_\tau-\mu)[\bar{a}_-(\mathbf{r})a_-(\mathbf{r}) + \bar{a}_+(\mathbf{r})a_+(\mathbf{r})] - U\bar{a}_-(\mathbf{r})\bar{a}_+(\mathbf{r})a_+(\mathbf{r})a_-(\mathbf{r}), \tag{17}$$

where $\bar{a}$ and a are Grassmann field. Using the Hubbard-Stratonovich (HS) transformation, we can introduce a Bosonic field:

$$1 = \int D[\Delta,\bar{\Delta}]e^{-U\int_0^\beta d\tau\int d\mathbf{r}[\bar{\Delta}(\mathbf{r})-\bar{a}_-(\mathbf{r})\bar{a}_+(\mathbf{r})][\Delta(\mathbf{r})-a_+(\mathbf{r})a_-(\mathbf{r})]}. \tag{18}$$

As such we can rewrite our partition function as:

$$Z = \int D[\Delta,\bar{\Delta}]e^{-U\int_0^\beta d\tau\int d\mathbf{r}|\mathbf{\Delta}(\mathbf{r})|^2}\mathcal{Z}[\Delta,\bar{\Delta}], \tag{19}$$
$$\mathcal{Z}[\Delta,\bar{\Delta}] = \int D[a,\bar{a}]e^{-\int_0^\beta d\tau\int d\mathbf{r}\mathcal{L}[\Delta,\bar{\Delta},a,\bar{a}]}, \tag{20}$$
$$\mathcal{L}[\Delta,\bar{\Delta},a,\bar{a}] = \mathcal{L}_{\rm Dirac}[a,\bar{a}] + (\partial_\tau-\mu)[\bar{a}_-(\mathbf{r})a_-(\mathbf{r}) + \bar{a}_+(\mathbf{r})a_+(\mathbf{r})] - U[\bar{\Delta}(\mathbf{r})a_+(\mathbf{r})a_-(\mathbf{r}) + \text{h.c.}]. \tag{21}$$

We can introduce the projection of an annihilation operator onto the relevant band as:

$$a_{\tau s\lambda}(\mathbf{r}) \to \mathcal{A}\int \frac{d^2\mathbf{q}}{(2\pi)^2} e^{i\mathbf{q}\cdot\mathbf{r}} u^*_{v,-,\tau}(\mathbf{q}) c(\mathbf{q}), \tag{22}$$

where $c_\tau(\mathbf{q})$ is the the band electron annihilation operator of momentum $\mathbf{q}$, and $\mathcal{A}$ is the unit cell area of the lattice. As such we can project our Lagrangian density to $\mathcal{L}[\Delta,\bar{\Delta},c,\bar{c}]$:

$$\begin{aligned}\mathcal{L}[\Delta,\bar{\Delta},a,\bar{a}] \to &\mathcal{L}[\Delta,\bar{\Delta},c,\bar{c}]\\ &= [\epsilon_0(\mathbf{q}) + \partial_\tau - \mu][\bar{c}(\mathbf{q})c(\mathbf{q}) + \bar{c}(\mathbf{q})c(\mathbf{q})]\\ &\quad - U\mathcal{A}\int \frac{d^2\mathbf{k}}{(2\pi)^2}\left[\bar{\Delta}(\mathbf{k})\Gamma(\mathbf{q},\mathbf{k})c\left(\mathbf{q}+\frac{\mathbf{k}}{2}\right)c\left(-\mathbf{q}+\frac{\mathbf{k}}{2}\right) + \text{h.c.}\right],\end{aligned} \tag{23}$$

where we have redefined $c_\tau(\mathbf{q})$ as the corresponding Grassmann field. Note that the form factor is defined by $\Gamma(\mathbf{q},\mathbf{k}) = u^T_{v,-,+}\left(\mathbf{q}+\frac{\mathbf{k}}{2}\right)u_{v,-,-}\left(-\mathbf{q}+\frac{\mathbf{k}}{2}\right)$. We have also assume the bosonic field $\Delta(\mathbf{q})$ transforms trivially as a spin 0 boson $\Delta(\mathbf{r}) \to \mathcal{A}\int \frac{d^2\mathbf{k}}{(2\pi)^2} e^{i\mathbf{k}\cdot\mathbf{r}}\Delta(\mathbf{k})$. We can rewrite our Lagrangian density into a matrix form using the Nambu spinors:

$$\mathcal{L}[\Delta,\bar{\Delta},c,\bar{c}] = \mathcal{A}\int \frac{d^2\mathbf{q}'}{(2\pi)^2}\begin{pmatrix}\bar{c}(\mathbf{q}') & c(-\mathbf{q}')\end{pmatrix} G_{\mathbf{q}',\mathbf{q}}\begin{pmatrix}c(\mathbf{q})\\ \bar{c}(-\mathbf{q})\end{pmatrix}. \tag{24}$$

Defining the free energy of the system as $Z = \int D[\Delta,\bar{\Delta}]e^{-\beta F[\Delta,\bar{\Delta}]}$, or equivalently as:

$$\begin{aligned}F[\Delta,\bar{\Delta}] &= \int \frac{d^2\mathbf{k}}{(2\pi)^2}U|\mathbf{\Delta}(\mathbf{k})|^2 - T\ln\int D[c,\bar{c}]\ e^{-\int_0^\beta d\tau\int \frac{d^2\mathbf{q}}{(2\pi)^2}\mathcal{L}[\Delta,\bar{\Delta},c,\bar{c}]}\\ &= \int \frac{d^2\mathbf{k}}{(2\pi)^2}U|\Delta(\mathbf{k})|^2 - T\int \frac{d^2\mathbf{q}}{(2\pi)^2}\int \frac{d^2\mathbf{q}'}{(2\pi)^2}\ln\det G_{\mathbf{q},\mathbf{q}'}.\end{aligned} \tag{25}$$

To calculate the free energy, we expand the Bosonic field $\Delta(\mathbf{k})$ around the extremum of the free energy as:

$$\Delta(\mathbf{k}) = \Delta_0\delta(\mathbf{k}) + \delta\Delta(\mathbf{k}), \tag{26}$$

where Δ_0 is the mean-field solution, real when properly gauged, and $\delta\Delta(\mathbf{k})$ is the fluctuation. We can thus decompose our Lagrangian density $\mathcal{L}[\Delta,\bar{\Delta},c,\bar{c}]$ as:

$$\mathcal{L}[\Delta,\bar{\Delta},c,\bar{c}] = \mathcal{L}_0 + \delta\mathcal{L} \tag{27}$$

$$\mathcal{L}_0 = [\epsilon_0(\mathbf{q}) + \partial_\tau - \mu][\bar{c}(\mathbf{q})c(\mathbf{q}) + \bar{c}(\mathbf{q})c(\mathbf{q})] - U[\Gamma(\mathbf{q},0)\Delta_0 c(\mathbf{q})c(-\mathbf{q}) + \text{h.c.}] \tag{28}$$

$$\delta\mathcal{L} = -U\int \frac{d^2\mathbf{k}}{(2\pi)^2}\left[\Gamma(\mathbf{q},\mathbf{k})\delta\bar{\Delta}(\mathbf{k})c\left(\mathbf{q}+\frac{\mathbf{k}}{2}\right)c\left(-\mathbf{q}+\frac{\mathbf{k}}{2}\right) + \text{h.c.}\right]. \tag{29}$$

Correspondingly, we can decompose the free energy $F[\Delta,\bar{\Delta}]$ as:

$$F[\Delta,\bar{\Delta}] = F_0 + \delta F, \tag{30}$$

where F_0 would be given by:

$$\begin{aligned}F_0 &= \int \frac{d^2\mathbf{q}}{(2\pi)^2}\left\{U|\mathbf{\Delta}_0|^2 - T\sum_n \ln\left[\beta^2\det\begin{pmatrix}\epsilon_0(\mathbf{q}) - i\omega_n - \mu & -U\Gamma(\mathbf{q},0)\Delta_0\\ -(U\Gamma(\mathbf{q},0)\Delta_0)^* & -\epsilon_0(\mathbf{q}) - i\omega_n + \mu\end{pmatrix}\right]\right\}\\ &= \int \frac{d^2\mathbf{q}}{(2\pi)^2}\left\{U|\Delta_0|^2 - T\sum_n \ln\beta^2[-\omega_n^2 - (\epsilon_0(\mathbf{q}) - \mu)^2 - |U\Gamma(\mathbf{q},0)\Delta_0|^2]\right\}\\ &= \int \frac{d^2\mathbf{q}}{(2\pi)^2}\left\{U|\Delta_0|^2 - T\sum_n \{\ln\beta[-i\omega_n + \epsilon(\mathbf{q})] + \ln\beta[-i\omega_n - \epsilon(\mathbf{q})]\}\right\}\\ &= \int \frac{d^2\mathbf{q}}{(2\pi)^2}\left\{U|\Delta_0|^2 - T\left[\ln\left(1 + e^{-\beta\epsilon(\mathbf{q})}\right) + \ln\left(1 + e^{\beta\epsilon(\mathbf{q})}\right)\right]\right\}\\ &= \int \frac{d^2\mathbf{q}}{(2\pi)^2}\left[U|\Delta_0|^2 - \epsilon(\mathbf{q}) - 2T\ln\left(1 + e^{-\beta\epsilon(\mathbf{q})}\right)\right].\end{aligned} \tag{31}$$

Here $\epsilon(\mathbf{q}) = \sqrt{(\epsilon_0(\mathbf{q})-\mu)^2 + |U\Gamma(\mathbf{q},0)\Delta_0|^2}$ and $\omega_n = 2n\pi T$ is the Matsubara frequencies. Note that F_0 recovers the form of the grand potential. We should also mention the self-consistency condition for Δ_0, namely that $\frac{\partial F_0}{\partial \Delta_0} = 0$ gives us:

$$\begin{aligned}
0 &= \int \frac{d^2\mathbf{q}}{(2\pi)^2}\left[2U\Delta_0 - \frac{\partial \epsilon(\mathbf{q})}{\partial \Delta_0}\left(1 - \frac{2e^{-\beta\epsilon(\mathbf{q})}}{1+e^{-\beta\epsilon(\mathbf{q})}}\right)\right] \\
&= \int \frac{d^2\mathbf{q}}{(2\pi)^2}\left[2U\Delta_0 - \frac{U^2|\Gamma(\mathbf{q},0)|^2\Delta_0}{\epsilon(\mathbf{q})}\tanh\frac{\beta\epsilon(\mathbf{q})}{2}\right] \\
&= \int \frac{d^2\mathbf{q}}{(2\pi)^2}\left[1 - \frac{U|\Gamma(\mathbf{q},0)|^2}{2\epsilon(\mathbf{q})}\tanh\frac{\beta\epsilon(\mathbf{q})}{2}\right] \\
1 &= \mathcal{A}\int \frac{d^2\mathbf{q}}{(2\pi)^2}\frac{U|\Gamma(\mathbf{q},0)|^2}{2\epsilon(\mathbf{q})}\tanh\frac{\beta\epsilon(\mathbf{q})}{2}.
\end{aligned} \tag{32}$$

The higher order contribution δF is given by:

$$\begin{aligned}
\delta F &= \int \frac{d^2\mathbf{k}}{(2\pi)^2}\left[U|\delta\Delta|^2 - T\ln\frac{\int D[c,\bar{c}]\ e^{-\int_0^\beta d\tau\int\frac{d^2\mathbf{q}}{(2\pi)^2}(\mathcal{L}_0+\delta\mathcal{L})}}{\int D[c,\bar{c}]\ e^{-\int_0^\beta d\tau\int\frac{d^2\mathbf{q}}{(2\pi)^2}\mathcal{L}_0}}\right] \\
&= \int \frac{d^2\mathbf{k}}{(2\pi)^2}\left[U|\delta\Delta|^2 - T\left\langle e^{-\int_0^\beta d\tau\int\frac{d^2\mathbf{q}}{(2\pi)^2}\delta\mathcal{L}} - 1\right\rangle\right],
\end{aligned} \tag{33}$$

where we have defined the thermal average $\langle f\rangle = \left(\int D[c,\bar{c}]\ e^{-\int_0^\beta d\tau\int\frac{d^2\mathbf{q}}{(2\pi)^2}\mathcal{L}_0}\right)^{-1}\int D[c,\bar{c}]\ e^{-\int_0^\beta d\tau\int\frac{d^2\mathbf{q}}{(2\pi)^2}\mathcal{L}_0} f$. To facilitate the calculation of Gor'kov's Green function thus δF, we should introduce generating functional:

$$\mathcal{Z}[\eta,\bar{\eta}] = \int D[\psi,\bar{\psi}]\ e^{-\int_0^\beta d\tau\int\frac{d^2\mathbf{q}}{(2\pi)^2}\left[\bar{\psi}(\mathbf{q})G_0(\mathbf{q})\psi(\mathbf{q}) - \bar{\eta}(\mathbf{q})\psi(\mathbf{q}) - \bar{\psi}(\mathbf{q})\eta(\mathbf{q})\right]}, \tag{34}$$

where $\bar{\psi}(\mathbf{q}) = \begin{pmatrix}\bar{c}_-(\mathbf{q}) & c_+(-\mathbf{q})\end{pmatrix}$ is the Nambu spinor, and $\mathcal{L}_0 = \bar{\psi}(\mathbf{q})G_0(\mathbf{q})\psi(\mathbf{q})$. By performing shifting on the field $\psi(\mathbf{q}) \to \psi(\mathbf{q}) + G_0^{-1}(\mathbf{q})\eta(\mathbf{q})$ and $\bar{\psi}(\mathbf{q}) \to \bar{\psi}(\mathbf{q}) + \bar{\eta}(\mathbf{q})G_0^{-1}(\mathbf{q})$, we arrive at:

$$\mathcal{Z}[\eta,\bar{\eta}] = \mathcal{Z}[0,0]\ e^{\int_0^\beta d\tau\int\frac{d^2\mathbf{q}}{(2\pi)^2}\bar{\eta}(\mathbf{q})G_0^{-1}(\mathbf{q})\eta(\mathbf{q})}. \tag{35}$$

As such thermal average $\langle\psi_{\mathbf{q}}\bar{\psi}_{\mathbf{q}'}\rangle$ takes the form:

$$\begin{aligned}
\langle\psi_{\mathbf{q}}\bar{\psi}_{\mathbf{q}'}\rangle &= \frac{\delta}{\delta\eta(\mathbf{q}')}\frac{\delta}{\delta\bar{\eta}(\mathbf{q})}e^{\int_0^\beta d\tau\int\frac{d^2\mathbf{q}}{(2\pi)^2}\bar{\eta}(\mathbf{k})G_0^{-1}(\mathbf{k})\eta(\mathbf{k})}\bigg|_{\eta,\bar{\eta}=0} \\
&= \frac{\delta}{\delta\eta(\mathbf{q}')}\left[G_0^{-1}(\mathbf{q})\eta(\mathbf{q})e^{\int_0^\beta d\tau\int\frac{d^2\mathbf{q}}{(2\pi)^2}\bar{\eta}(\mathbf{k})G_0^{-1}(\mathbf{k})\eta(\mathbf{k})}\right]\bigg|_{\eta,\bar{\eta}=0} \\
&= G_0^{-1}(\mathbf{q})\delta_{\mathbf{q},\mathbf{q}'}.
\end{aligned} \tag{36}$$

where $G_0^{-1}(\mathbf{q})$ has the expression:

$$G_0^{-1}(\mathbf{q}) = \frac{1}{\omega_n^2+\epsilon^2(\mathbf{q})}\begin{pmatrix}\epsilon_0(\mathbf{q})+i\omega_n-\mu & -U\Gamma(\mathbf{q},0)\Delta_0 \\ -U\Gamma^*(\mathbf{q},0)\Delta_0 & -\epsilon_0(\mathbf{q})+i\omega_n+\mu\end{pmatrix}. \tag{37}$$

As such, we have Gor'kov's Green functions:

$$\mathcal{G}(\mathbf{q}) = \langle c(\mathbf{q})\bar{c}(\mathbf{q})\rangle = \frac{i\omega_n+\epsilon_0(\mathbf{q})-\mu}{\omega_n^2+\epsilon^2(\mathbf{q})}, \tag{38}$$

$$\mathcal{F}(\mathbf{q}) = \langle c(-\mathbf{q})c(\mathbf{q})\rangle = -\frac{U\Gamma^*(\mathbf{q},0)\Delta_0}{\omega_n^2+\epsilon^2(\mathbf{q})}. \tag{39}$$

Note that due to time reversal invariant, $\Gamma(\mathbf{q},0) = 1$, thus $\epsilon(\mathbf{q}) = \sqrt{(\epsilon_0(\mathbf{q})-\mu)^2 + U^2\Delta_0^2}$. With all information, we can solve for δF. Due to stability of the mean-field solution, a linear correction has to vanish, and the lowest order

term is thus second order in $\delta\Delta(\mathbf{k})$, namely F_2 the Gaussian fluctuation of the free energy, given by:

$$
\begin{aligned}
F_2 &= \int \frac{d^2\mathbf{k}}{(2\pi)^2} U|\delta\Delta(\mathbf{k})|^2 - \frac{T}{2}\left\langle \left(\int_0^\beta d\tau \int \frac{d^2\mathbf{k}}{(2\pi)^2}\delta\mathcal{L}\right)^2 \right\rangle \\
&= \int \frac{d^2\mathbf{k}}{(2\pi)^2} U|\delta\Delta(\mathbf{k})|^2 \\
&- \frac{T}{2}\sum_n \mathcal{A}\int \frac{d^2\mathbf{q}}{(2\pi)^2}\int \frac{d^2\mathbf{k}}{(2\pi)^2}\Big[2|\Gamma(\mathbf{q},\mathbf{k})|^2|\delta\Delta(\mathbf{k})|^2 \left\langle c\left(\mathbf{q}+\frac{\mathbf{k}}{2}\right)\bar{c}\left(\mathbf{q}+\frac{\mathbf{k}}{2}\right)\right\rangle\left\langle c\left(-\mathbf{q}+\frac{\mathbf{k}}{2}\right)\bar{c}\left(-\mathbf{q}+\frac{\mathbf{k}}{2}\right)\right\rangle \\
&+ \Gamma(\mathbf{q},\mathbf{k})\Gamma(\mathbf{q},-\mathbf{k})\delta\Delta(\mathbf{k})\delta\Delta(-\mathbf{k})\left\langle \bar{c}\left(\mathbf{q}+\frac{\mathbf{k}}{2}\right)\bar{c}\left(-\mathbf{q}-\frac{\mathbf{k}}{2}\right)\right\rangle\left\langle \bar{c}\left(-\mathbf{q}+\frac{\mathbf{k}}{2}\right)\bar{c}\left(\mathbf{q}-\frac{\mathbf{k}}{2}\right)\right\rangle \\
&+ \Gamma^*(\mathbf{q},\mathbf{k})\Gamma^*(\mathbf{q},-\mathbf{k})\delta\Delta^*(\mathbf{k})\delta\Delta^*(-\mathbf{k})\left\langle c\left(-\mathbf{q}+\frac{\mathbf{k}}{2}\right)c\left(\mathbf{q}+\frac{\mathbf{k}}{2}\right)\right\rangle\left\langle c\left(\mathbf{q}+\frac{\mathbf{k}}{2}\right)c\left(-\mathbf{q}-\frac{\mathbf{k}}{2}\right)\right\rangle\Big] \\
&= \int \frac{d^2\mathbf{k}}{(2\pi)^2}|\delta\Delta(\mathbf{k})|^2\left[U - U^2\chi(\mathbf{k})\right]
\end{aligned} \tag{40}
$$

$$
\chi(\mathbf{q}) = T\sum_n \mathcal{A}\int \frac{d^2\mathbf{q}}{(2\pi)^2}|\Gamma(\mathbf{q},\mathbf{k})|^2\left[\mathcal{G}\left(\mathbf{q}+\frac{\mathbf{k}}{2}\right)\mathcal{G}\left(-\mathbf{q}+\frac{\mathbf{k}}{2}\right) + \mathcal{F}\left(\mathbf{q}+\frac{\mathbf{k}}{2}\right)\mathcal{F}\left(-\mathbf{q}+\frac{\mathbf{k}}{2}\right)\right]. \tag{41}
$$

III. QUANTUM METRIC OF MASSIVE DIRAC CONES

Assuming time reversal invariant, form factor $|\Gamma(\mathbf{q},\mathbf{k})|^2$ can be expanded given that $\mathbf{k}$ is small as:

$$
\begin{aligned}
|\Gamma(\mathbf{q},\mathbf{k})|^2 &= \left|\langle u_{\mathbf{q}+\mathbf{k}/2}|\, u_{\mathbf{q}-\mathbf{k}/2}\rangle\right|^2 \\
&= \mathrm{Tr}\left[P\left(\mathbf{q}+\frac{\mathbf{k}}{2}\right)P\left(\mathbf{q}-\frac{\mathbf{k}}{2}\right)\right] \\
&= \mathrm{Tr}\left\{\left[P(\mathbf{q}) + \sum_i \frac{k_i}{2}\partial_i P(\mathbf{q}) + \sum_{i,j}\frac{k_ik_j}{8}\partial_i\partial_j P(\mathbf{q})\right]\left[P(\mathbf{q}) - \sum_a \frac{k_a}{2}\partial_a P(\mathbf{q}) + \sum_{a,b}\frac{k_ak_b}{8}\partial_a\partial_b P(\mathbf{q})\right]\right\} \\
&= 1 - \sum_{i,j}\frac{k_ik_j}{8}\mathrm{Tr}\left[2\partial_i P(\mathbf{q})\partial_j P(\mathbf{q}) - P(\mathbf{q})\partial_i\partial_j P(\mathbf{q}) - \partial_i\partial_j P(\mathbf{q})P(\mathbf{q})\right] \\
&= 1 - \sum_{i,j}\frac{k_ik_j}{8}\mathrm{Tr}\left[4\partial_i P(\mathbf{q})\partial_j P(\mathbf{q}) - \partial_i\partial_j P(\mathbf{q})\right] \\
&= 1 - \sum_{i,j}\frac{1}{2}k_ik_j\mathrm{Tr}\left[\partial_i P(\mathbf{q})\partial_j P(\mathbf{q})\right] + \frac{k_ik_j}{8}\partial_i\partial_j \mathrm{Tr}P(\mathbf{q}) \\
&= 1 - \sum_{i,j}\frac{1}{2}k_ik_j\mathrm{Tr}\left[\partial_i P(\mathbf{q})\partial_j P(\mathbf{q})\right] \\
&= 1 - \sum_{i,j}k_ik_jg_{ij}(\mathbf{q}),
\end{aligned} \tag{42}
$$

where $g_{ij}(\mathbf{q}) = \frac{1}{2}\mathrm{Tr}\left[\partial_i P(\mathbf{q})\partial_j P(\mathbf{q})\right]$ is the quantum metric. For our toy model, in terms of elementary matrices, the projector matrix is given by:

$$\begin{aligned}
P &= \frac{1}{2\epsilon_0(\mathbf{q})}\begin{pmatrix} e^{-i\phi/2}\sqrt{\epsilon_0(\mathbf{q})+m} \\ e^{i\phi/2}\sqrt{\epsilon_0(\mathbf{q})-m}\end{pmatrix}\begin{pmatrix} e^{i\phi/2}\sqrt{\epsilon_0(\mathbf{q})+m} & e^{-i\phi/2}\sqrt{\epsilon_0(\mathbf{q})-m}\end{pmatrix} \\
&= \frac{1}{2\epsilon_0(\mathbf{q})}\begin{pmatrix} \epsilon_0(\mathbf{q})+m & e^{-i\phi}\sqrt{\epsilon_0(\mathbf{q})^2-m^2} \\ e^{i\phi}\sqrt{\epsilon_0(\mathbf{q})^2-m^2} & \epsilon_0(\mathbf{q})-m\end{pmatrix} \\
&= \frac{1}{2\epsilon_0(\mathbf{q})}\begin{pmatrix} \epsilon_0(\mathbf{q})+m & vqe^{-i\phi} \\ vqe^{i\phi} & \epsilon_0(\mathbf{q})-m\end{pmatrix} \\
&= \frac{1}{2}\left(\mathbb{1}_2 + \frac{m}{\epsilon_0(\mathbf{q})}\sigma_z + \frac{v}{\epsilon_0(\mathbf{q})}\mathbf{q}\cdot\boldsymbol{\sigma}\right).
\end{aligned} \tag{43}$$

As such the derivative is given by:

$$\begin{aligned}
\partial_i P(\mathbf{q}) &= \frac{1}{2\epsilon_0(\mathbf{q})}\left[v\sigma_i - \frac{\partial_i\epsilon_0(\mathbf{q})}{\epsilon_0(\mathbf{q})}(m\sigma_z + v\mathbf{q}\cdot\boldsymbol{\sigma})\right] \\
&= \frac{1}{2\epsilon_0(\mathbf{q})}\left[v\sigma_i - \frac{v^2 q_i}{\epsilon_0(\mathbf{q})^2}(m\sigma_z + v\mathbf{q}\cdot\boldsymbol{\sigma})\right] \\
&= \frac{v}{2\epsilon_0(\mathbf{q})^3}\left[\epsilon_0(\mathbf{q})^2\sigma_i - vq_i(m\sigma_z + v\mathbf{q}\cdot\boldsymbol{\sigma})\right] \\
&= \frac{v}{2\epsilon_0(\mathbf{q})^3}\left[\left(m^2 + v^2 q_j^2 + iv^2 q_i q_j \sigma_z \epsilon_{ij}\right)\sigma_i - mvq_i\sigma_z\right] && (44) \\
&= \frac{v}{2\epsilon_0(\mathbf{q})^3}\left[\sigma_i\left(m^2 + v^2 q_j^2 - iv^2 q_i q_j \sigma_z \epsilon_{ij}\right) - mvq_i\sigma_z\right], && (45)
\end{aligned}$$

where $j \neq i$, and $\epsilon_{xy} = -\epsilon_{yx} = 1$. Correspondingly quantum geometry is:

$$\begin{aligned}
g_{ii}(\mathbf{q}) &= \frac{1}{2}\mathrm{Tr}[\partial_i P(\mathbf{q})\partial_i P(\mathbf{q})] \\
&= \frac{v^2}{8\epsilon_0(\mathbf{q})^6}\left[\left(m^2 + v^2 q_j^2\right)^2 + v^4 q_i^2 q_j^2 + m^2 v^2 q_i^2\right]\mathrm{Tr}\mathbb{1}_2 \\
&= \frac{v^2}{4\epsilon_0(\mathbf{q})^6}\left(m^4 + 2m^2v^2q_j^2 + v^4 q_j^4 + v^4 q_i^2 q_j^2 + m^2 v^2 q_i^2\right) \\
&= \frac{v^2}{4\epsilon_0(\mathbf{q})^6}\left(m^4 + m^2v^2q_j^2 + v^4 q^2 q_j^2 + m^2 v^2 q^2\right) \\
&= \frac{v^2}{4\epsilon_0(\mathbf{q})^4}\left(m^2 + v^2 q_j^2\right) && (46) \\
g_{ij}(\mathbf{q}, i \neq j) &= \frac{1}{2}\mathrm{Tr}[\partial_i P(\mathbf{q})\partial_i P(\mathbf{q})] \\
&= \frac{v^2}{8\epsilon_0(\mathbf{q})^6}\left\{\mathrm{Tr}\left[i\epsilon_{ij}\sigma_z\left(m^2 + v^2 q_i^2 + iv^2 q_i q_j \sigma_z \epsilon_{ij}\right)\left(m^2 + v^2 q_j^2 + iv^2 q_i q_j \sigma_z \epsilon_{ij}\right)\right] + 2m^2v^2 q_i q_j\right\} \\
&= \frac{v^2}{8\epsilon_0(\mathbf{q})^6}\left\{\mathrm{Tr}\left[i\epsilon_{ij}\sigma_z\left(2m^2 + v^2 q^2\right) iv^2 q_i q_j \sigma_z \epsilon_{ij}\right] + 2m^2v^2 q_i q_j\right\} \\
&= \frac{v^2}{4\epsilon_0(\mathbf{q})^6}\left[-\left(2m^2 + v^2 q^2\right)v^2 q_i q_j + m^2 v^2 q_i q_j\right] \\
&= \frac{v^2}{4\epsilon_0(\mathbf{q})^6}\left[-\left(2m^2 + v^2 q^2\right)v^2 q_i q_j + m^2 v^2 q_i q_j\right] \\
&= -\frac{v^2}{4\epsilon_0(\mathbf{q})^4}v^2 q_i q_j && (47) \\
g(\mathbf{q}) &= \frac{v^2}{4(m^2 + v^2q^2)^2}\begin{pmatrix} m^2 + v^2 q_y^2 & -v^2 q_x q_y \\ -v^2 q_x q_y & m^2 + v^2 q_x^2\end{pmatrix}. && (48)
\end{aligned}$$

We will use this result to compare between the conventional and the geometrical contribution in the later session.

IV. GINZBURG-LANDAU THEORY OF MASSIVE DIRAC CONE

At the upper critical field H_{c2}, the order parameter is suppressed, namely $\Delta_0 = 0$. In this situation, $\epsilon(\mathbf{q}) = |\epsilon_0(\mathbf{q}) - \mu|$, and Gor'kov's anomalous Green Function (39) vanishes, with the normal Green Function (38) simplifies to:

$$\mathcal{G}(\mathbf{q}) = \frac{1}{-i\omega_n + \epsilon_0(\mathbf{q}) - \mu}. \tag{49}$$

Recall Eq. (40) where the free energy density takes the form $F_2 = \int \frac{d^2\mathbf{k}}{(2\pi)^2} |\delta\Delta(\mathbf{k})|^2 \left[U - U^2\chi(\mathbf{k})\right]$, where:

$$\begin{aligned}
\chi(\mathbf{k}) &= T\sum_n \mathcal{A}\int \frac{d^2\mathbf{q}}{(2\pi)^2}|\Gamma(\mathbf{q},\mathbf{k})|^2 \mathcal{G}\left(\mathbf{q}+\frac{\mathbf{k}}{2}\right)\mathcal{G}\left(-\mathbf{q}+\frac{\mathbf{k}}{2}\right) \\
&= T\mathcal{A}\int \frac{d^2\mathbf{q}}{(2\pi)^2}|\Gamma(\mathbf{q},\mathbf{k})|^2 \sum_n \frac{1}{-i\omega_n + \epsilon_0\left(\mathbf{q}+\frac{\mathbf{k}}{2}\right) - \mu}\frac{1}{i\omega_n + \epsilon_0\left(-\mathbf{q}+\frac{\mathbf{k}}{2}\right) - \mu} \\
&= \frac{1}{2}\mathcal{A}\int \frac{d^2\mathbf{q}}{(2\pi)^2}|\Gamma(\mathbf{q},\mathbf{k})|^2 \frac{\tanh\frac{\beta}{2}\left[\epsilon_0\left(\mathbf{q}+\frac{\mathbf{k}}{2}\right)-\mu\right] + \tanh\frac{\beta}{2}\left[\epsilon_0\left(\mathbf{q}-\frac{\mathbf{k}}{2}\right)-\mu\right]}{\epsilon_0\left(\mathbf{q}+\frac{\mathbf{k}}{2}\right) + \epsilon_0\left(\mathbf{q}-\frac{\mathbf{k}}{2}\right) - 2\mu} \\
&= \mathcal{A}\int \frac{d^2\mathbf{q}}{(2\pi)^2}|\Gamma(\mathbf{q},\mathbf{k})|^2 \frac{\tanh\left[\frac{\beta}{2}\varepsilon\left(\mathbf{q}+\frac{\mathbf{k}}{2}\right)\right] + \tanh\left[\frac{\beta}{2}\varepsilon\left(\mathbf{q}-\frac{\mathbf{k}}{2}\right)\right]}{2\left[\varepsilon\left(\mathbf{q}+\frac{\mathbf{k}}{2}\right) + \varepsilon\left(\mathbf{q}-\frac{\mathbf{k}}{2}\right)\right]}.
\end{aligned} \tag{50}$$

In the last line, we have defined $\varepsilon(\mathbf{q}) = \epsilon_0(\mathbf{q}) - \mu$ for convenience. Note that the conduction band dispersion is invariant under rotational symmetry, thus isotropic. As such, we can perform expansion around $\mathbf{k} = 0$ to obtain:

$$\chi(\mathbf{k}) = \chi_0 - \left(\chi_{2,\mathrm{con}} + \chi_{2,\mathrm{qm}}\right)k^2 + \mathcal{O}(k^4), \tag{51}$$

$$\chi_0 = \mathcal{A}\int \frac{d^2\mathbf{q}}{(2\pi)^2}\frac{1}{2\varepsilon(\mathbf{q})}\tanh\frac{\beta\varepsilon(\mathbf{q})}{2}, \tag{52}$$

$$\chi_{2,\mathrm{qm}}k^2 = \frac{k^2}{2}\mathcal{A}\int \frac{d^2\mathbf{q}}{(2\pi)^2}\mathrm{tr}g(\mathbf{q})\frac{\tanh\left[\frac{\beta}{2}\varepsilon(\mathbf{q})\right]}{2\varepsilon(\mathbf{q})}, \tag{53}$$

$$\chi_{2,\mathrm{con}}k^2 = \mathcal{A}\int \frac{d^2\mathbf{q}}{(2\pi)^2}\left\{\frac{\tanh\left[\frac{\beta}{2}\varepsilon(\mathbf{q})\right]}{2\varepsilon(\mathbf{q})} - \frac{\tanh\left[\frac{\beta}{2}\varepsilon\left(\mathbf{q}+\frac{\mathbf{k}}{2}\right)\right] + \tanh\left[\frac{\beta}{2}\varepsilon\left(\mathbf{q}-\frac{\mathbf{k}}{2}\right)\right]}{2\left[\varepsilon\left(\mathbf{q}+\frac{\mathbf{k}}{2}\right) + \varepsilon\left(\mathbf{q}-\frac{\mathbf{k}}{2}\right)\right]}\right\}, \tag{54}$$

where $\chi_{2,\mathrm{con}}$ is contribution due to dispersion of the band, and $\chi_{2,\mathrm{qm}}$ is due to the form factor, thus the quantum metric. Note the zeroth order term can be rewritten as:

$$\begin{aligned}
\chi_0 &= \mathcal{A}\int \frac{d^2\mathbf{q}}{(2\pi)^2}\frac{\tanh\left[\frac{\beta}{2}\varepsilon(\mathbf{q})\right]}{2\varepsilon(\mathbf{q})} \\
&= \mathcal{A}\int \frac{d^2\mathbf{q}}{(2\pi)^2}\frac{\tanh\left[\frac{\beta}{2}\varepsilon(\mathbf{q})\right] - \tanh\left[\frac{\beta_{\mathrm{MF}}}{2}\varepsilon(\mathbf{q})\right]}{2\varepsilon(\mathbf{q})} + \frac{1}{U},
\end{aligned} \tag{55}$$

where $T_{\mathrm{MF}} = \frac{1}{\beta_{\mathrm{MF}}}$is the mean-field critical temperature, defined by the continuous version of the self-consistency condition (32) when $\Delta_0 = 0$:

$$\frac{1}{U} = \mathcal{A}\int \frac{d^2\mathbf{q}}{(2\pi)^2}\frac{1}{2\varepsilon(\mathbf{q})}\tanh\left[\frac{\beta_{\mathrm{MF}}}{2}\varepsilon(\mathbf{q})\right]. \tag{56}$$

In the following, we will perform analytical calculations for the nearly massless regime, namely the mass gap of the Dirac bands, is much smaller than the Debye energy, which defines the energy range where we have effective attractive interaction. In particular, we will study the case where the mass gap $m \gg T_{\mathrm{MF}}$ and $m \ll T_{\mathrm{MF}}$.

A. Case 1: $m \gg T_{\mathrm{MF}}$

In continuous model, we can treat the chemical potential as a free parameter. In particular, we will set it to $|\mu| = m + \kappa\epsilon_D$, and with $0 \le \kappa \le 1$. To begin with we should write down our integral over momentum space in terms

of energy:

$$\int \frac{d^2\mathbf{q}}{(2\pi)^2} = \int_m^{m+(1+\nu)\epsilon_D} \frac{\epsilon_0 d\epsilon_0}{2\pi v^2} = \int_{-\kappa\epsilon_D}^{\epsilon_D} \frac{(\varepsilon+\mu)d\varepsilon}{2\pi v^2}. \tag{57}$$

Considering the contribution from quantum metric, we have:

$$\begin{aligned}
\chi_{2,\mathrm{qm}} k^2 &= \frac{k^2}{2}\mathcal{A}\int \frac{d^2\mathbf{q}}{(2\pi)^2}(g_{xx}+g_{yy})\frac{\tanh\left[\frac{\beta}{2}\varepsilon(\mathbf{q})\right]}{2\varepsilon(\mathbf{q})} \\
&= \frac{1}{2}k^2\mathcal{A}\int \frac{d^2\mathbf{q}}{(2\pi)^2}\frac{v^2(2m^2+v^2q^2)}{4(m^2+v^2q^2)^2}\frac{\tanh\left[\frac{\beta}{2}\varepsilon(\mathbf{q})\right]}{2\varepsilon(\mathbf{q})} \\
&= \frac{1}{4}k^2\mathcal{A}\int_{-\kappa\epsilon_D}^{\epsilon_D} \frac{d\varepsilon}{2\pi}\frac{1}{2}\frac{m^2+(\varepsilon+\mu)^2}{(\varepsilon+\mu)^3}\frac{\tanh\left(\frac{\beta}{2}\varepsilon\right)}{2\varepsilon} \\
&= \frac{1}{4}k^2\mathcal{A}\left[\int_{-\kappa\epsilon_D}^{\epsilon_D} \frac{d\varepsilon}{2\pi}\frac{1}{2}\frac{m^2}{(\varepsilon+\mu)^3}\frac{\tanh\left(\frac{\beta}{2}\varepsilon\right)}{2\varepsilon} + \int_{-\kappa\epsilon_D}^{\epsilon_D} \frac{d\varepsilon}{2\pi}\frac{1}{2}\frac{\tanh\left(\frac{\beta}{2}\varepsilon\right)}{2\varepsilon(\varepsilon+\mu)}\right].
\end{aligned} \tag{58}$$

For $\kappa\beta\epsilon_D \gg 1$, the latter term dominates:

$$\begin{aligned}
\chi_{2,\mathrm{qm}} k^2 &= \frac{1}{4}k^2\mathcal{A}\int_{-\kappa\epsilon_D}^{\epsilon_D} \frac{d\varepsilon}{2\pi}\frac{1}{2}\frac{\tanh\left(\frac{\beta}{2}\varepsilon\right)}{2\varepsilon(\varepsilon+\mu)} \\
&= \frac{1}{32\pi}k^2\mathcal{A}\int_{-\kappa\epsilon_D}^{\epsilon_D} d\varepsilon\,\frac{1}{\mu}\left(\frac{1}{\varepsilon}-\frac{1}{\varepsilon+\mu}\right)\tanh\left(\frac{\beta}{2}\varepsilon\right) \\
&\approx \frac{1}{32\pi}k^2\mathcal{A}\int_{-\kappa\epsilon_D}^{\epsilon_D} d\varepsilon\,\frac{1}{\mu}\frac{1}{\varepsilon}\tanh\left(\frac{\beta}{2}\varepsilon\right) - \frac{1}{16\pi}k^2\mathcal{A}\int_{-\kappa\epsilon_D}^{\epsilon_D} d\varepsilon\,\frac{1}{\mu}\frac{1}{\varepsilon+\mu} \\
&\approx \frac{1}{32\pi}k^2\mathcal{A}\frac{1}{\mu}\left[2\ln\left(\frac{4e^\gamma}{\pi}\frac{\beta\epsilon_D}{2}\right)+\ln\kappa-\ln\frac{(\epsilon_D+\mu)m}{\mu^2}\right] \\
&\approx \frac{k^2\mathcal{A}}{32\pi\mu}\left[\ln\left(\frac{\beta^2\mu^3\epsilon_D}{m(\mu+\epsilon_D)}\right)+2\ln\left(\frac{2e^\gamma}{\pi}\right)\right] \\
&\approx \frac{k^2\mathcal{A}}{32\pi\mu}\ln\left(\frac{\beta^2\mu^3}{m}\right).
\end{aligned} \tag{59}$$

where we have used the approximation $\int_0^a dx\tanh(x)/x \approx \ln(4e^\gamma a/\pi)$ for $a \gg 1$.

For $\kappa\beta\epsilon_D \ll 1$, both term contributes. We can appoximate the values of each term by setting $\kappa = 0$. The first term is:

$$\begin{aligned}
\frac{1}{4}k^2\mathcal{A}\int_0^{\epsilon_D} \frac{d\varepsilon}{2\pi}\frac{1}{2}\frac{m^2}{(\varepsilon+\mu)^3}\frac{\tanh\left(\frac{\beta}{2}\varepsilon\right)}{2\varepsilon} &= \frac{1}{32\pi}k^2\mathcal{A}m^2\int_0^{\epsilon_D} d\varepsilon\frac{\tanh\left(\frac{\beta}{2}\varepsilon\right)}{\varepsilon(\varepsilon+m)^3} \\
&\approx \frac{1}{32\pi}k^2\mathcal{A}m^2\left[\int_0^{2/\beta} d\varepsilon\frac{\beta}{2(\varepsilon+m)^3}+\int_{2/\beta}^{\epsilon_D} d\varepsilon\frac{1}{\varepsilon(\varepsilon+m)^3}\right] \\
&\approx \frac{1}{32\pi}k^2\mathcal{A}m^2\left\{\frac{\beta}{4}\left[\frac{1}{m^2}-\frac{1}{(m+2T)^2}\right]\right. \\
&\left.+\frac{1}{2m^3}\left[\frac{2m\epsilon_D}{\epsilon_D^2}+2\ln\left(\frac{\epsilon_D}{\epsilon_D+m}\right)-\frac{m(3m+4T)}{(m+2T)^2}-2\ln\left(\frac{2T}{m+2T}\right)\right]\right\} \\
&\approx \frac{k^2\mathcal{A}}{64\pi m}\left[-1-2\ln 2+2\ln(\beta m)\right].
\end{aligned}$$

The latter term is:

$$\begin{aligned}
\frac{1}{4}k^2\mathcal{A}\int_0^{\epsilon_D}\frac{d\varepsilon}{2\pi}\frac{1}{2}\frac{\tanh\left(\frac{\beta}{2}\varepsilon\right)}{2\varepsilon\left(\varepsilon+\mu\right)} &= \frac{1}{32\pi}k^2\mathcal{A}\int_0^{\epsilon_D}d\varepsilon\ \frac{1}{\mu}\left(\frac{1}{\varepsilon}-\frac{1}{\varepsilon+\mu}\right)\tanh\left(\frac{\beta}{2}\varepsilon\right) \\
&\approx \frac{1}{32\pi}k^2\mathcal{A}\int_0^{\epsilon_D}d\varepsilon\ \frac{1}{\mu}\frac{1}{\varepsilon}\tanh\left(\frac{\beta}{2}\varepsilon\right)-\frac{1}{16\pi}k^2\mathcal{A}\int_0^{\epsilon_D}d\varepsilon\ \frac{1}{\mu}\frac{1}{\varepsilon+\mu} \\
&\approx \frac{1}{32\pi}k^2\mathcal{A}\frac{1}{m}\left[\ln\left(\frac{2e^\gamma}{\pi}\beta\epsilon_D\right)-\ln\left(1+\frac{\epsilon_D}{m}\right)\right] \\
&\approx \frac{k^2\mathcal{A}}{32\pi m}\left[\ln\left(\frac{2e^\gamma}{\pi}\right)+\ln\left(\beta m\right)\right].
\end{aligned}$$

Thus the total contribution at $\kappa=0$ (namely $\mu=m$) is:

$$\chi_{\mathrm{qm}}(\kappa=0)k^2=\frac{k^2\mathcal{A}}{64\pi m}\left[-1+2\ln\left(\frac{e^\gamma}{\pi}\right)+4\ln\left(\beta m\right)\right]. \tag{60}$$

For conventional contribution, we have:

$$\begin{aligned}
\chi_{2,\mathrm{con}}k^2 &= \mathcal{A}\int\frac{d^2\mathbf{q}}{(2\pi)^2}\left\{\frac{\tanh\left[\frac{\beta}{2}\varepsilon\left(\mathbf{q}\right)\right]}{2\varepsilon(\mathbf{q})}-\frac{\tanh\left[\frac{\beta}{2}\varepsilon\left(\mathbf{q}+\frac{\mathbf{k}}{2}\right)\right]+\tanh\left[\frac{\beta}{2}\varepsilon\left(\mathbf{q}-\frac{\mathbf{k}}{2}\right)\right]}{2\left[\varepsilon\left(\mathbf{q}+\frac{\mathbf{k}}{2}\right)+\varepsilon\left(\mathbf{q}-\frac{\mathbf{k}}{2}\right)\right]}\right\} \\
&= \mathcal{A}\int\frac{d^2\mathbf{q}}{(2\pi)^2}\frac{1}{2}\left\{\frac{1-2n_F\left[\varepsilon\left(\mathbf{q}\right)\right]}{2\varepsilon(\mathbf{q})}+\frac{n_F\left[\varepsilon\left(\mathbf{q}+\frac{\mathbf{k}}{2}\right)\right]+n_F\left[\varepsilon\left(\mathbf{q}-\frac{\mathbf{k}}{2}\right)\right]-1}{2\varepsilon(\mathbf{q})}\right\} \\
&\approx \mathcal{A}\int\frac{d^2\mathbf{q}}{(2\pi)^2}\frac{1}{2\varepsilon(\mathbf{q})}\partial_\varepsilon^2 n_F[\varepsilon(\mathbf{q})]\left\{\frac{v^2(\mathbf{q}\cdot\mathbf{k})}{2[\varepsilon(\mathbf{q})+\mu]}\right\}^2 \\
&\approx \mathcal{A}\int\frac{qdq}{2\pi}\frac{1}{2\varepsilon(\mathbf{q})}\partial_\varepsilon^2 n_F[\varepsilon(\mathbf{q})]\frac{v^4q^2k^2}{8[\varepsilon(\mathbf{q})+\mu]^2} \\
&= \mathcal{A}\int_{-\kappa\epsilon_D}^{\epsilon_D}\frac{d\varepsilon}{32\pi}\frac{1}{\varepsilon}\partial_\varepsilon^2 n_F(\varepsilon)\frac{k^2}{\varepsilon+\mu}\left[(\varepsilon+\mu)^2-m^2\right] \\
&= k^2\mathcal{A}\int_{-\kappa\epsilon_D}^{\epsilon_D}\frac{d\varepsilon}{32\pi}\partial_\varepsilon^2 n_F(\varepsilon)\left[1+\frac{\mu}{\varepsilon}-\frac{m^2}{\varepsilon(\varepsilon+\mu)}\right] \\
&= k^2\mathcal{A}\int_{-\kappa\epsilon_D}^{\epsilon_D}\frac{d\varepsilon}{32\pi}\partial_\varepsilon^2 n_F(\varepsilon)\left[1+\frac{\mu}{\varepsilon}\left(1-\frac{m^2}{\mu^2}\right)+\frac{m^2}{\mu(\varepsilon+\mu)}\right].
\end{aligned} \tag{61}$$

For case where $\kappa\beta\epsilon_D\gg 1$, we have:

$$\begin{aligned}
\chi_{2,\mathrm{con}}k^2 &\approx k^2\mathcal{A}\int_{-\kappa\epsilon_D}^{\epsilon_D}\frac{d\varepsilon}{32\pi}\partial_\varepsilon^2 n_F(\varepsilon)\left[1+\frac{\mu}{\varepsilon}\left(1-\frac{m^2}{\mu^2}\right)+\frac{m^2}{\mu(\varepsilon+\mu)}\right] \\
&\approx k^2\mathcal{A}\int_{-\kappa\epsilon_D}^{\epsilon_D}\frac{d\varepsilon}{32\pi}\partial_\varepsilon^2 n_F(\varepsilon)+k^2\mu\mathcal{A}\left(1-\frac{m^2}{\mu^2}\right)\int_{-\kappa\epsilon_D}^{\epsilon_D}\frac{d\varepsilon}{32\pi}\frac{\partial_\varepsilon^2 n_F(\varepsilon)}{\varepsilon} \\
&\approx k^2\mathcal{A}\frac{1}{32\pi}\beta\left(e^{-\kappa\beta\epsilon_D}-e^{-\beta\epsilon_D}\right)+k^2\mu\mathcal{A}\frac{1}{32\pi}\left(1-\frac{m^2}{\mu^2}\right)\int_{-\infty}^{\infty}d\varepsilon\frac{\partial_\varepsilon^2 n_F(\varepsilon)}{\varepsilon} \\
&\approx k^2\mu\mathcal{A}\frac{1}{32\pi}\left(1-\frac{m^2}{\mu^2}\right)\beta^2\frac{7\zeta(3)}{2\pi^2} \\
&\approx k^2\mathcal{A}\frac{7\zeta(3)}{64\pi^3}\frac{\kappa\epsilon_D}{T^2}.
\end{aligned} \tag{62}$$

For case where $|\kappa|\beta\epsilon_D \ll 1$, we have:

$$\begin{aligned}
\chi_{2,\mathrm{con}}k^2 &\approx k^2\mathcal{A}\int_{-\kappa\epsilon_D}^{\epsilon_D}\frac{d\varepsilon}{32\pi}\partial_\varepsilon^2 n_F(\varepsilon)\left[1+\frac{\mu}{\varepsilon}\left(1-\frac{m^2}{\mu^2}\right)+\frac{m^2}{\mu(\varepsilon+\mu)}\right]\\
&\approx k^2\mathcal{A}\int_{-\kappa\epsilon_D}^{\epsilon_D}\frac{d\varepsilon}{32\pi}\partial_\varepsilon^2 n_F(\varepsilon)\left(1+\frac{m}{\varepsilon}\frac{2\kappa\epsilon_D}{m}+\frac{m}{\varepsilon+m+\kappa\epsilon_D}\right)\\
&\approx \frac{k^2\mathcal{A}}{32\pi}\left[\int_{-\kappa\epsilon_D}^{\epsilon_D}d\varepsilon\ \partial_\varepsilon^2 n_F(\varepsilon)+2\kappa\epsilon_D\int_0^{\epsilon_D}d\varepsilon\ \frac{\partial_\varepsilon^2 n_F(\varepsilon)}{\varepsilon}+m\int_{-\kappa\epsilon_D}^{\epsilon_D}d\varepsilon\ \frac{\partial_\varepsilon^2 n_F(\varepsilon)}{\varepsilon+m+\kappa\epsilon_D}\right]\\
&\approx \frac{k^2\mathcal{A}}{32\pi}\left[\beta\frac{e^{-\kappa\beta\epsilon_D}}{(1+e^{-\kappa\beta\epsilon_D})^2}+2\kappa\epsilon_D\beta^2\frac{7\zeta(3)}{4\pi^2}+\beta\frac{e^{-\kappa\beta\epsilon_D}}{(1+e^{-\kappa\beta\epsilon_D})^2}\right]\\
&\approx \frac{k^2\mathcal{A}}{32\pi}\left[\frac{\beta}{2}+2\kappa\epsilon_D\beta^2\frac{7\zeta(3)}{4\pi^2}\right]\\
&\approx \frac{k^2\mathcal{A}}{64\pi T}\left[1+\kappa\beta\epsilon_D\frac{7\zeta(3)}{\pi^2}\right].
\end{aligned}\tag{63}$$

As for the mean-field temperature, we have:

$$\begin{aligned}
\frac{1}{U} &= \mathcal{A}\int_{-\kappa\epsilon_D}^{\epsilon_D}\frac{(\varepsilon+\mu)d\varepsilon}{2\pi v^2}\frac{1}{2\varepsilon}\tanh\frac{\beta_{\mathrm{MF}}\varepsilon}{2}\\
&= \frac{\mathcal{A}}{4\pi v^2}\int_{-\kappa\epsilon_D}^{\epsilon_D}d\varepsilon\left(\tanh\frac{\beta_{\mathrm{MF}}\varepsilon}{2}+\frac{\mu}{\varepsilon}\tanh\frac{\beta_{\mathrm{MF}}\varepsilon}{2}\right)\\
&= \frac{\mathcal{A}}{4\pi v^2}\left[\frac{2}{\beta_{MF}}\ln\frac{\cosh(\beta_{\mathrm{MF}}\epsilon_D/2)}{\cosh(\kappa\beta_{\mathrm{MF}}\epsilon_D/2)}+\mu\int_{-\kappa\epsilon_D}^{\epsilon_D}d\varepsilon\ \frac{1}{\varepsilon}\tanh\frac{\beta_{\mathrm{MF}}\varepsilon}{2}\right].
\end{aligned}\tag{64}$$

Finally we should calculate $U-U^2\chi_0$:

$$\begin{aligned}
U-U^2\chi_0 &= -U^2\mathcal{A}\int\frac{d^2\mathbf{q}}{(2\pi)^2}\frac{\tanh\left[\frac{\beta}{2}\varepsilon(\mathbf{q})\right]-\tanh\left[\frac{\beta_{\mathrm{MF}}}{2}\varepsilon(\mathbf{q})\right]}{2\varepsilon(\mathbf{q})}\\
&\approx -U^2\mathcal{A}\int\frac{d^2\mathbf{q}}{(2\pi)^2}\frac{\mathrm{sech}^2\left[\frac{\beta_{\mathrm{MF}}}{2}\varepsilon(\mathbf{q})\right]}{2\varepsilon(\mathbf{q})}\frac{\beta-\beta_{\mathrm{MF}}}{2}\varepsilon(\mathbf{q})\\
&= -U^2\mathcal{A}\frac{1}{v^2}\int_{-\kappa\epsilon_D}^{\epsilon_D}\frac{d\varepsilon}{2\pi}(\varepsilon+\mu)\frac{\mathrm{sech}^2\left(\frac{\beta_{\mathrm{MF}}\varepsilon}{2}\right)}{4}(\beta-\beta_{\mathrm{MF}})\\
&= -U^2\mathcal{A}\frac{1}{8\pi v^2}(\beta-\beta_{\mathrm{MF}})\frac{2}{\beta_{\mathrm{MF}}}\left\{\left[(\varepsilon+m+\kappa\epsilon_D)\tanh\frac{\beta_{\mathrm{MF}}\varepsilon}{2}\right]\Bigg|_{-\kappa\epsilon_D}^{\epsilon_D}-\int_{-\kappa\epsilon_D}^{\epsilon_D}d\varepsilon\tanh\frac{\beta_{\mathrm{MF}}\varepsilon}{2}\right\}\\
&= \frac{U^2\mathcal{A}}{4\pi v^2}\frac{\beta_{\mathrm{MF}}-\beta}{\beta_{\mathrm{MF}}}\left\{[m+(1+\kappa)\epsilon_D]\tanh\frac{\beta_{\mathrm{MF}}\epsilon_D}{2}+m\tanh\frac{\kappa\beta_{\mathrm{MF}}\epsilon_D}{2}\right.\\
&\left.-\frac{2}{\beta_{MF}}\left(\ln\cosh\frac{\beta_{\mathrm{MF}}\epsilon_D}{2}-\ln\cosh\frac{\kappa\beta_{\mathrm{MF}}\epsilon_D}{2}\right)\right\},
\end{aligned}\tag{65}$$

where for convenience we have defined $\alpha = 4\pi v^2/U\mathcal{A}$. For $\kappa\beta\epsilon_D \gg 1$, we have:

$$\begin{aligned}
\alpha &\approx \frac{2}{\beta_{MF}}\ln\frac{\cosh(\beta_{\mathrm{MF}}\epsilon_D/2)}{\cosh(\kappa\beta_{\mathrm{MF}}\epsilon_D/2)}+\mu\int_{-\kappa\epsilon_D}^{\epsilon_D}d\varepsilon\ \frac{1}{\varepsilon}\tanh\frac{\beta_{\mathrm{MF}}\varepsilon}{2}\\
&\approx (1-\kappa)\epsilon_D+2(m+\kappa\epsilon_D)\ln\left(\frac{2e^\gamma}{\pi}\beta_{\mathrm{MF}}\epsilon_D\right)+(m+\kappa\epsilon_D)\ln\kappa\\
T_{\mathrm{MF}} &\approx \frac{2\sqrt{\kappa}}{\pi}\epsilon_D\exp\left(\gamma+\frac{1-\kappa}{2\kappa}-\frac{\alpha}{2\kappa\epsilon_D}\right).
\end{aligned}\tag{66}$$

For $\kappa\beta\epsilon_D \ll 1$, we have:

$$\begin{aligned}\alpha &\approx \frac{2}{\beta_{MF}}\ln\frac{\cosh(\beta_{\rm MF}\epsilon_D/2)}{\cosh(\kappa\beta_{\rm MF}\epsilon_D/2)} + \mu\int_{-\kappa\epsilon_D}^{\epsilon_D} d\varepsilon\ \frac{1}{\varepsilon}\tanh\frac{\beta_{\rm MF}\varepsilon}{2} \\ &\approx \epsilon_D + (m+\kappa\epsilon_D)\ln\left(\frac{2e^\gamma}{\pi}\beta_{\rm MF}\epsilon_D\right) + (m+\kappa\epsilon_D)\frac{\kappa\beta_{\rm MF}\epsilon_D}{2} \\ &\approx \epsilon_D + (m+\kappa\epsilon_D)\ln\left(\frac{2e^\gamma}{\pi}\beta_{\rm MF}\epsilon_D\right) \\ T_{\rm MF} &\approx \frac{2}{\pi}\epsilon_D\exp\left(\gamma + \frac{\epsilon_D-\alpha}{\mu}\right). \end{aligned} \tag{67}$$

We should also calculate $U - U^2\chi_0$ to determine the coherence length:

$$\begin{aligned} U - U^2\chi_0 &= -U^2\mathcal{A}\int\frac{d^2\mathbf{q}}{(2\pi)^2}\frac{\tanh\left[\frac{\beta}{2}\varepsilon(\mathbf{q})\right] - \tanh\left[\frac{\beta_{\rm MF}}{2}\varepsilon(\mathbf{q})\right]}{2\varepsilon(\mathbf{q})} \\ &\approx -U^2\mathcal{A}\int\frac{d^2\mathbf{q}}{(2\pi)^2}\frac{\operatorname{sech}^2\left[\frac{\beta_{\rm MF}}{2}\varepsilon(\mathbf{q})\right]}{2\varepsilon(\mathbf{q})}\frac{\beta-\beta_{\rm MF}}{2}\varepsilon(\mathbf{q}) \\ &= -U^2\mathcal{A}\frac{1}{v^2}\int_{-\kappa\epsilon_D}^{\epsilon_D}\frac{d\varepsilon}{2\pi}(\varepsilon+\mu)\frac{\operatorname{sech}^2\left(\frac{\beta_{\rm MF}\varepsilon}{2}\right)}{4}(\beta-\beta_{\rm MF}) \\ &= -U^2\mathcal{A}\frac{1}{8\pi v^2}(\beta-\beta_{\rm MF})\frac{2}{\beta_{\rm MF}}\left\{\left[(\varepsilon+m+\kappa\epsilon_D)\tanh\frac{\beta_{\rm MF}\varepsilon}{2}\right]\Bigg|_{-\kappa\epsilon_D}^{\epsilon_D} - \int_{-\kappa\epsilon_D}^{\epsilon_D} d\varepsilon\tanh\frac{\beta_{\rm MF}\varepsilon}{2}\right\} \\ &= \frac{U^2\mathcal{A}}{4\pi v^2}\frac{\beta_{\rm MF}-\beta}{\beta_{\rm MF}}\left\{[m+(1+\kappa)\epsilon_D]\tanh\frac{\beta_{\rm MF}\epsilon_D}{2} + m\tanh\frac{\kappa\beta_{\rm MF}\epsilon_D}{2}\right. \\ &\left. -\frac{2}{\beta_{MF}}\left(\ln\cosh\frac{\beta_{\rm MF}\epsilon_D}{2} - \ln\cosh\frac{\kappa\beta_{\rm MF}\epsilon_D}{2}\right)\right\}. \end{aligned} \tag{68}$$

Specifying to the case where $\epsilon_D \gg m,\ \kappa\beta_{\rm MF}\epsilon_D \gg 1$, we have:

$$\begin{aligned} U - U^2\chi_0 &\approx \frac{U}{\alpha}\frac{\beta_{\rm MF}-\beta}{\beta_{\rm MF}}\left\{(1+\kappa)\epsilon_D + m - \frac{2}{\beta_{MF}}\left(\frac{\beta_{\rm MF}\epsilon_D}{2} - \frac{\kappa\beta_{\rm MF}\epsilon_D}{2}\right)\right\} \\ &\approx 2\kappa\epsilon_D\frac{U}{\alpha}\frac{\beta_{\rm MF}-\beta}{\beta_{\rm MF}}. \end{aligned} \tag{69}$$

For $\kappa\beta\epsilon_D \ll 1$, we have:

$$\begin{aligned} U - U^2\chi_0 &\approx \frac{U}{\alpha}\frac{\beta_{\rm MF}-\beta}{\beta_{\rm MF}}\left\{[m+(1+\kappa)\epsilon_D] - \frac{2}{\beta_{MF}}\left(\frac{\beta_{\rm MF}\epsilon_D}{2} - \ln 2\right)\right\} \\ &\approx \mu\frac{U}{\alpha}\frac{\beta_{\rm MF}-\beta}{\beta_{\rm MF}}. \end{aligned} \tag{70}$$

Note that we can write down an effective Lagrangian for our bosonic field:

$$\begin{aligned} \mathcal{L}[\delta\Delta,\delta\bar{\Delta}] &= \int\frac{d^2\mathbf{k}}{(2\pi)^2}\ F_2(\mathbf{k}) \\ &= (U-U^2\chi_0)|\delta\Delta|^2 + (\chi_{2,\rm qm}+\chi_{2,\rm con})|\partial_{\mathbf{k}}\delta\Delta|^2. \end{aligned} \tag{71}$$

As such, the coherence length should be given by:

$$\begin{aligned} \xi &= \sqrt{-U^2\frac{\chi_{2,\rm qm}+\chi_{2,\rm con}}{U-U^2\chi_0}} \\ &= \sqrt{-\frac{U^2\chi_{2,\rm qm}}{U-U^2\chi_0} - \frac{U\chi_{2,\rm con}}{U-U^2\chi_0}} \\ &= \sqrt{\xi_{\rm qm}^2 + \xi_{\rm con}^2}. \end{aligned} \tag{72}$$

At the $\kappa\beta_{\rm MF}\epsilon_D \gg 1$ regime, the coherence lengths are given by:

$$
\begin{aligned}
\xi_{\rm qm} &= \sqrt{-\frac{U^2\chi_{2,\rm qm}}{U-U^2\chi_0}} \\
&\approx \sqrt{-\frac{\frac{U^2\mathcal{A}}{32\pi\kappa\epsilon_D}\left[\ln\left(\frac{\epsilon_D^3}{mT_{\rm MF}^2}\right)+2\ln\left(\frac{2e^\gamma}{\pi}\right)+\ln\left(\frac{\kappa^3}{1+\kappa}\right)\right]}{2\kappa\epsilon_D\frac{U^2\mathcal{A}}{4\pi v^2}\frac{\beta_{\rm MF}-\beta}{\beta_{\rm MF}}}} \\
&= \frac{v}{4\mu}\left(\frac{T_{\rm MF}-T}{T_{\rm MF}}\right)^{-1/2}\sqrt{\ln\left(\frac{\mu^3}{mT_{\rm MF}^2}\right)} \qquad (73) \\
\xi_{\rm con} &= \sqrt{-\frac{U^2\chi_{2,\rm con}}{U-U^2\chi_0}} \\
&\approx \sqrt{-\frac{U^2\mathcal{A}\frac{7\zeta(3)}{64\pi^3}\frac{\kappa\epsilon_D}{T_{\rm MF}^2}}{2\kappa\epsilon_D\frac{U^2\mathcal{A}}{4\pi v^2}\frac{\beta_{\rm MF}-\beta}{\beta_{\rm MF}}}} \\
&= \frac{v}{T_{\rm MF}}\left(\frac{T_{\rm MF}-T}{T_{\rm MF}}\right)^{-1/2}\sqrt{\frac{7\zeta(3)}{32\pi^2}}. \qquad (74)
\end{aligned}
$$

As such, the conventional contribution dominates, by a factor of $\epsilon_D/T_{\rm MF} \gg 1$. For $\kappa=0$, the coherence lengths are given by:

$$
\begin{aligned}
\xi_{\rm qm} &= \sqrt{-\frac{U^2\chi_{2,\rm qm}}{U-U^2\chi_0}} \\
&\approx \sqrt{-\frac{\frac{U^2\mathcal{A}}{64\pi m}\left[-1+2\ln\left(\frac{e^\gamma}{\pi}\right)+4\ln\left(\beta_{\rm MF}m\right)\right]}{m\frac{U^2\mathcal{A}}{4\pi v^2}\frac{\beta_{\rm MF}-\beta}{\beta_{\rm MF}}}} \\
&= \frac{v}{4m}\left(\frac{T_{\rm MF}-T}{T_{\rm MF}}\right)^{-1/2}\sqrt{-1+2\ln\left(\frac{e^\gamma}{\pi}\right)+4\ln\left(\beta_{\rm MF}m\right)} \qquad (75) \\
\xi_{\rm con} &= \sqrt{-\frac{U^2\chi_{2,\rm con}}{U-U^2\chi_0}} \\
&\approx \sqrt{-\frac{\frac{U^2\mathcal{A}}{64\pi T_{\rm MF}}}{m\frac{U^2\mathcal{A}}{4\pi v^2}\frac{\beta_{\rm MF}-\beta}{\beta_{\rm MF}}}} \\
&= \frac{1}{4}\frac{v}{\sqrt{mT_{\rm MF}}}\left(\frac{T_{\rm MF}-T}{T_{\rm MF}}\right)^{-1/2}. \qquad (76)
\end{aligned}
$$

Note that in this case the quantum metric contribution still smaller, but is comparable with the conventional contribution by a factor of $2\sqrt{\ln(\beta_{\rm MF}m)/\beta_{\rm MF}m}$.

1. Beyond band edge

In this part, we aim to provide a brief discussion on the case beyond the band edge. We assume $||\mu|-m| \ll m$ such that $\chi_{2,\rm qm} = \chi_{2,\rm qm}(\kappa=0)$, corresponding to $\mu=m$ with $\chi_{2,qm}(\kappa)$ defined in Eq. (60). We should calculate $U-U^2\chi_0$ in the vicinity where superconductivity vanishes, namely when $|\beta_{\rm MF}(||\mu|-m|)| \gg 0$:

$$
\begin{aligned}
&\frac{U^2\mathcal{A}}{4\pi v^2}\frac{\beta_{\rm MF}-\beta}{\beta_{\rm MF}}\left[(\mu+\epsilon_D)\tanh\frac{\beta_{\rm MF}\epsilon_D}{2}+m\tanh\frac{\beta_{\rm MF}(\mu-m)}{2}-\frac{2}{\beta_{MF}}\left(\ln\cosh\frac{\beta_{\rm MF}\epsilon_D}{2}+\ln\cosh\frac{\beta_{\rm MF}(\mu-m)}{2}\right)\right]\\
\approx&\frac{U^2\mathcal{A}}{4\pi v^2}\frac{\beta_{\rm MF}-\beta}{\beta_{\rm MF}}\left[\mu+\epsilon_D-m-\frac{2}{\beta_{MF}}\left(\frac{\beta_{\rm MF}\epsilon_D}{2}+\frac{\beta_{\rm MF}(\mu-m)}{2}\right)\right]+\frac{4}{\beta_{\rm MF}}\ln 2\\
=&\frac{U^2\mathcal{A}}{4\pi v^2}\frac{\beta_{\rm MF}-\beta}{\beta_{\rm MF}}4T_{\rm MF}\ln 2. \qquad (77)
\end{aligned}
$$

which vanishes as the mean-field transition temperature goes to zero due to vanishing density of state. As such the quantum metric coherence length diverges at boundary of phase transition. Note that the conventional contribution remains finite until $T_{\rm MF}\to 0$ due to both $\chi_{2,\rm con}\propto\beta_{\rm MF}e^{\kappa\beta_{\rm MF}\epsilon_D}$ and $U-U^2\chi_0$ vanishing as $T_{\rm MF}\to 0$. It is important to note that the superconductivity exists due to a finite temperature effect, and is expected to vanish as temperature goes to 0.

B. Case 2: $T_{\rm MF}\gg m$

In this scenario, most of the calculation is identical to the previous case, with the exception of $\chi_{\rm qm}$ when $\mu\approx m$:

$$
\begin{aligned}
\frac{1}{4}k^2\mathcal{A}\int_0^{\epsilon_D}\frac{d\varepsilon}{2\pi}\frac{1}{2}\frac{\tanh\left(\frac{\beta}{2}\varepsilon\right)}{2\varepsilon\left(\varepsilon+\mu\right)}&=\frac{1}{32\pi}k^2\mathcal{A}\int_0^{\epsilon_D}d\varepsilon\;\frac{1}{\mu}\left(\frac{1}{\varepsilon}-\frac{1}{\varepsilon+\mu}\right)\tanh\left(\frac{\beta}{2}\varepsilon\right)\\
&=\frac{1}{32\pi}k^2\mathcal{A}\int_0^{\epsilon_D}d\varepsilon\;\frac{1}{\mu}\frac{1}{\varepsilon}\tanh\left(\frac{\beta}{2}\varepsilon\right)-\frac{1}{16\pi}k^2\mathcal{A}\int_0^{\epsilon_D}d\varepsilon\;\frac{1}{\mu}\frac{1}{\varepsilon+\mu}\tanh\left(\frac{\beta}{2}\varepsilon\right)\\
&\approx\frac{1}{32\pi}k^2\mathcal{A}\frac{1}{m}\left[\int_0^{\epsilon_D}d\varepsilon\;\frac{1}{\varepsilon}\tanh\left(\frac{\beta}{2}\varepsilon\right)-\int_m^{\epsilon_D}d\varepsilon\;\frac{1}{\varepsilon}\left(1-\frac{m}{\varepsilon}\right)\tanh\left(\frac{\beta}{2}\varepsilon\right)\right] \qquad (78)\\
&=\frac{1}{32\pi}k^2\mathcal{A}\int_m^{\epsilon_D}d\varepsilon\;\frac{1}{\varepsilon^2}\tanh\left(\frac{\beta}{2}\varepsilon\right)\\
&\approx\frac{k^2\mathcal{A}}{32\pi}\left[\int_m^{2/\beta}d\varepsilon\;\frac{\beta}{2\varepsilon}+\int_{2/\beta}^{\epsilon_D}d\varepsilon\;\frac{1}{\varepsilon^2}\right]\\
&\approx\frac{k^2\mathcal{A}}{32\pi}\left[\frac{\beta}{2}\ln\frac{2}{\beta m}-\frac{1}{\epsilon_D}+\frac{1}{2T}\right]\\
&\approx\frac{k^2\mathcal{A}}{64\pi T}\left(1-\ln\frac{\beta m}{2}\right). \qquad (79)
\end{aligned}
$$

As such, the coherence length at $\kappa=0$ should change accordingly:

$$
\begin{aligned}
\xi_{\rm qm}&=\sqrt{-\frac{U^2\chi_{2,\rm qm}}{U-U^2\chi_0}}\\
&\approx\sqrt{-\frac{\frac{U^2\mathcal{A}}{64\pi T_{\rm MF}}\left(1-\ln\frac{\beta_{\rm MF}m}{2}\right)}{m\frac{U^2\mathcal{A}}{4\pi v^2}\frac{\beta_{\rm MF}-\beta}{\beta_{\rm MF}}}}\\
&=\frac{1}{4}\frac{v}{\sqrt{mT_{\rm MF}}}\left(\frac{T_{\rm MF}-T}{T_{\rm MF}}\right)^{-1/2}\sqrt{1-\ln\frac{\beta_{\rm MF}m}{2}}, \qquad (80)\\
\xi_{\rm con}&=\sqrt{-\frac{U^2\chi_{2,\rm con}}{U-U^2\chi_0}}\\
&\approx\sqrt{-\frac{\frac{U^2\mathcal{A}}{64\pi T_{\rm MF}}}{m\frac{U^2\mathcal{A}}{4\pi v^2}\frac{\beta_{\rm MF}-\beta}{\beta_{\rm MF}}}}\\
&=\frac{1}{4}\frac{v}{\sqrt{mT_{\rm MF}}}\left(\frac{T_{\rm MF}-T}{T_{\rm MF}}\right)^{-1/2}. \qquad (81)
\end{aligned}
$$

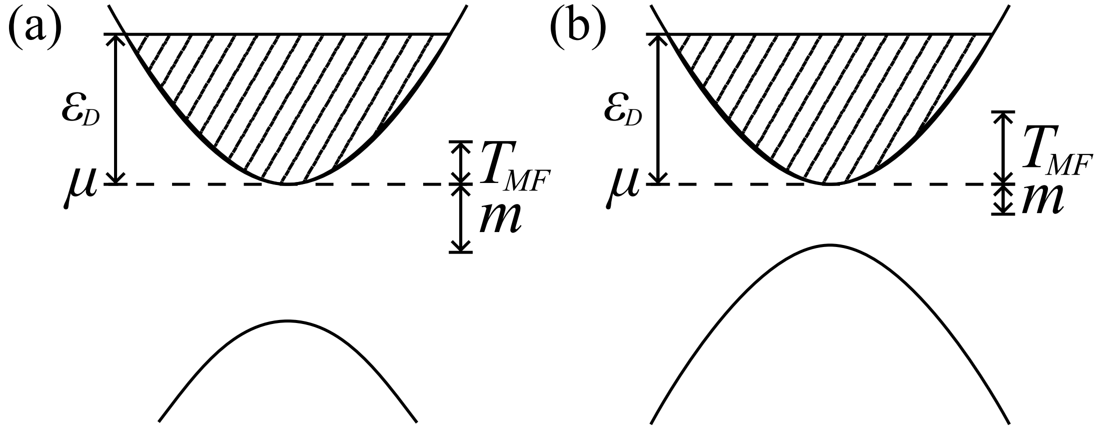

FIG. 1. Illustration of the energy scale and band structure for the toy model. The dashed area denote the energy range (ϵ_D) where the interaction is effectively attractive. In the figure we have assumed the chemical potential $\mu = m$, and $\epsilon_D \gg m$, T_{MF}. Figure (a) correspond to the case where $m \gg T_{\mathrm{MF}}$, while figure (b) correspond to the case where $m \ll T_{\mathrm{MF}}$

Note the two contributions share a similar form, with the quantum metric contribution having an extra factor of $\sqrt{1 - \ln(\beta_{\mathrm{MF}} m/2)} > 1$. The quantum metric, thus plays a dominating role in this regime, when superconductivity occurs near the band edge. The results for the toy model is summarized in Table II, alongside an illustration of the energy scale and band structure in Fig. 1.

V. DISCUSSION ON PAIRING AND TIME-REVERSAL SYMMETRY IN IVC

In this section, we aim to discuss the pairing of the IVC state, in order to induce superconductivity. To begin with, we can write down our IVC state in the valley basis $\begin{pmatrix} K \\ K' \end{pmatrix}$:

$$\psi_{-,\mathbf{q}} = \frac{1}{\sqrt{2}} \begin{pmatrix} e^{-i\phi_{\mathbf{q}}} \sqrt{1 - \frac{\Delta_z(\mathbf{q})}{|\mathbf{\Delta}(\mathbf{q})|}} \\ \sqrt{1 + \frac{\Delta_z(\mathbf{q})}{|\mathbf{\Delta}(\mathbf{q})|}} \end{pmatrix}, \tag{82}$$

$$\begin{aligned} \psi_{-,-\mathbf{q}} &= \frac{1}{\sqrt{2}} \begin{pmatrix} e^{-i\phi_{-\mathbf{q}}} \sqrt{1 - \frac{\Delta_z(-\mathbf{q})}{|\mathbf{\Delta}(-\mathbf{q})|}} \\ \sqrt{1 + \frac{\Delta_z(-\mathbf{q})}{|\mathbf{\Delta}(-\mathbf{q})|}} \end{pmatrix} \\ &= \frac{1}{\sqrt{2}} \begin{pmatrix} e^{-i\phi_{\mathbf{q}}} \sqrt{1 + \frac{\Delta_z(\mathbf{q})}{|\mathbf{\Delta}(\mathbf{q})|}} \\ \sqrt{1 - \frac{\Delta_z(\mathbf{q})}{|\mathbf{\Delta}(\mathbf{q})|}} \end{pmatrix}. \end{aligned} \tag{83}$$

Recall the IVC order parameter's phase can be matched with the phase of the inter-valley form factor $\langle u_{K,\mathbf{k}} | u_{K',\mathbf{k}} \rangle$[4], inducing a six-fold symmetry in the phase, resulting in $\phi_{\mathbf{q}} = \phi_{-\mathbf{q}}$. As such the form factor is given by:

$$\begin{aligned} \Gamma(\mathbf{q}, 0) &= \psi_{-,-\mathbf{q}}(K)\psi_{-,\mathbf{q}}(K') + \psi_{-,-\mathbf{q}}(K')\psi_{-,\mathbf{q}}(K) \\ &= \psi^T_{-,-\mathbf{q}} \sigma_x \psi_{-,\mathbf{q}} \\ &= \frac{1}{2} \left[\left(1 + \frac{\Delta_z}{|\mathbf{\Delta}|}\right) e^{-i\phi_{\mathbf{q}}} + \left(1 - \frac{\Delta_z}{|\mathbf{\Delta}|}\right) e^{-i\phi_{\mathbf{q}}} \right] \\ &= e^{-i\phi_{\mathbf{q}}} \end{aligned} \tag{84}$$

$$|\Gamma(\mathbf{q}, 0)|^2 = 1. \tag{85}$$

As such we have recovered time reversal invariant for the IVC state. With the invariant being intact after introducing the IVC order parameter, our previous calculation on the conventional coherence length remains valid. As for the

TABLE II. Summary on the toy model, where $\frac{1}{U'} = \frac{1}{U} - \frac{\epsilon_D \mathcal{A}}{4\pi v^2}$

<table>
<tr><th></th><th>$|\mu|$</th><th>$T_{\rm MF}$</th><th>$\xi_{\rm con}$</th><th>$\xi_{\rm qm}$</th></tr>
<tr><td>$m \gg T_{\rm MF}$</td><td rowspan="2">$\gg m$</td><td rowspan="2">$\propto \sqrt{\rho\epsilon_D}\exp\left(-\frac{1}{\rho U'}\right)$</td><td rowspan="2">$\propto \frac{v}{T_{\bf MF}}$</td><td rowspan="3">$\propto \frac{v}{\mu}\sqrt{\ln\left(\beta_{\rm MF}\mu\right)}$</td></tr>
<tr><td>$m \ll T_{\rm MF}$</td></tr>
<tr><td>$m \gg T_{\rm MF}$</td><td rowspan="2">$\to m$</td><td rowspan="2">$\propto \epsilon_D \exp\left(-\frac{2}{\rho U'}\right)$</td><td rowspan="2">$\propto \frac{v}{\sqrt{mT_{\rm MF}}}$</td></tr>
<tr><td>$m \ll T_{\rm MF}$</td><td>$\propto \frac{v}{\sqrt{mT_{\rm MF}}}\sqrt{1-\ln\frac{\beta_{\rm MF}m}{2}}$</td></tr>
</table>

projector matrices, we note the symmetry:

$$
\begin{aligned}
P(\mathbf{q}) &= \psi_{-,\mathbf{q}}\psi^\dagger_{-,\mathbf{q}} \\
&= \frac{1}{2}\begin{pmatrix} e^{-i\phi_{\mathbf{q}}}\sqrt{1-\frac{\Delta_z}{|\mathbf{\Delta}|}} \\ \sqrt{1+\frac{\Delta_z}{|\mathbf{\Delta}|}} \end{pmatrix}\begin{pmatrix} e^{i\phi_{\mathbf{q}}}\sqrt{1-\frac{\Delta_z}{|\mathbf{\Delta}|}} & \sqrt{1+\frac{\Delta_z}{|\mathbf{\Delta}|}} \end{pmatrix} \\
&= \frac{1}{2}\begin{pmatrix} 1-\frac{\Delta_z}{|\mathbf{\Delta}|} & e^{-i\phi_{\mathbf{q}}}\sqrt{1-\frac{\Delta_z^2}{|\mathbf{\Delta}|^2}} \\ e^{i\phi_{\mathbf{q}}}\sqrt{1-\frac{\Delta_z^2}{|\mathbf{\Delta}|^2}} & 1+\frac{\Delta_z}{|\mathbf{\Delta}|} \end{pmatrix},
\end{aligned}
\tag{86}
$$

$$
\begin{aligned}
\tilde{P}(-\mathbf{q}) &= \psi^*_{-,-\mathbf{q}}\psi^T_{-,-\mathbf{q}} \\
&= \frac{1}{2}\begin{pmatrix} e^{i\phi_{-\mathbf{q}}}\sqrt{1+\frac{\Delta_z}{|\mathbf{\Delta}|}} \\ \sqrt{1-\frac{\Delta_z}{|\mathbf{\Delta}|}} \end{pmatrix}\begin{pmatrix} e^{-i\phi_{-\mathbf{q}}}\sqrt{1+\frac{\Delta_z}{|\mathbf{\Delta}|}} & \sqrt{1-\frac{\Delta_z}{|\mathbf{\Delta}|}} \end{pmatrix} \\
&= \frac{1}{2}\begin{pmatrix} 1+\frac{\Delta_z}{|\mathbf{\Delta}|} & e^{i\phi_{\mathbf{q}}}\sqrt{1-\frac{\Delta_z^2}{|\mathbf{\Delta}|^2}} \\ e^{-i\phi_{\mathbf{q}}}\sqrt{1-\frac{\Delta_z^2}{|\mathbf{\Delta}|^2}} & 1-\frac{\Delta_z}{|\mathbf{\Delta}|} \end{pmatrix},
\end{aligned}
\tag{87}
$$

$$
\begin{aligned}
\sigma_x\tilde{P}(-\mathbf{q})\sigma_x &= \frac{1}{2}\begin{pmatrix} e^{-i\phi_{\mathbf{q}}}\sqrt{1-\frac{\Delta_z^2}{|\mathbf{\Delta}|^2}} & 1-\frac{\Delta_z}{|\mathbf{\Delta}|} \\ 1+\frac{\Delta_z}{|\mathbf{\Delta}|} & e^{i\phi_{\mathbf{q}}}\sqrt{1-\frac{\Delta_z^2}{|\mathbf{\Delta}|^2}} \end{pmatrix}\sigma_x \\
&= \frac{1}{2}\begin{pmatrix} 1-\frac{\Delta_z}{|\mathbf{\Delta}|} & e^{-i\phi_{\mathbf{q}}}\sqrt{1-\frac{\Delta_z^2}{|\mathbf{\Delta}|^2}} \\ e^{i\phi_{\mathbf{q}}}\sqrt{1-\frac{\Delta_z^2}{|\mathbf{\Delta}|^2}} & 1+\frac{\Delta_z}{|\mathbf{\Delta}|} \end{pmatrix} \\
&= P(\mathbf{q}).
\end{aligned}
\tag{88}
$$

Note that the expansion of form factor around $\mathbf{k}=0$ retrieves the quantum metric:

$$
\begin{aligned}
\left|\psi^T_{-,-\mathbf{q}}\sigma_x\psi_{-,\mathbf{q}+\mathbf{k}}\right|^2 &= \mathrm{Tr}\left[\sigma_x\tilde{P}(-\mathbf{q})\sigma_x P(\mathbf{q}+\mathbf{k})\right] \\
&= \mathrm{Tr}[P(\mathbf{q})P(\mathbf{q}+\mathbf{k})] \\
&= 1 - \frac{1}{2}\sum_{i,j} k_i k_j \mathrm{Tr}[\partial_i P(\mathbf{q})\partial_j P(\mathbf{q})] \\
&= 1 - \sum_{i,j} k_i k_j g_{ij}(\mathbf{q}),
\end{aligned}
\tag{89}
$$

where the intermediate step is similar to (42) thus are not shown explicitly. As such, our results obtained for quantum metric coherence length from bare electrons, is also applicable for superconductivity originating from the IVC state, besides a change in the Bloch state, thus the quantum metric. This justifies the application of the Ginzburg-Landau theory developed in the toy model section, to study the superconductivity originating from the IVC state in RTG.

VI. DETAILS OF FITTING PROCEDURE FOR U AND Δ_{IVC}

In this section, we aim to provide details of the fitting procedures for the effective attractive interaction strength U and the IVC order parameter Δ_{IVC} for RTG.

In section "Discrepancies in superconductivity of RTG", the effective attractive interaction strength U is obtained from the linearized gap equation (32) assuming effective attractive interaction between quasiparticles (15). To be precise, we projected onto the valance band at the Fermi energy of the flavor-symmetric 6-band Hamiltonian of RTG [2], which is justified due to the band gap separating the conduction and valence bands. The effective attractive interaction strength $U = 4$meV is fitted such that (32) reproduced the maximal value of transition temperature observed experimentally $T_c \sim 120$mK at the charge carrier density $n = -1.85 \times 10^{12}\mathrm{cm}^{-2}$.

In Section "Numerical calculations on the microscopic model", the IVC order parameter Δ_{IVC} is fitted using the IVC quasiparticle band structure. From the quasiparticle band structure, we identify the charge carrier density at the band edge, which corresponds to the phase boundary between the IVC-SC and IVC state. We then shift the phase boundary by tuning the IVC order parameter, until we can match the charge carrier density of $n \approx -1.8 \times 10^{12}\mathrm{cm}^{-2}$, where a sharp disappearance of superconductivity is observed in the experiment. The fitting procedure for effective attractive interaction strength U is similar to section "Discrepancies in superconductivity of RTG". However since the effective attractive interaction is between IVC quasiparticles, we have to instead project onto the lower energy IVC quasiparticle band. Following the procedure previously discussed we obtain effective attractive interaction strength of $U = 1.2$meV instead.

SUPPLEMENTARY REFERENCES

[1] F. Zhang, B. Sahu, H. Min, and A. H. MacDonald, Band structure of *abc*-stacked graphene trilayers, Phys. Rev. B **82**, 035409 (2010).

[2] H. Zhou, T. Xie, A. Ghazaryan, T. Holder, J. R. Ehrets, E. M. Spanton, T. Taniguchi, K. Watanabe, E. Berg, M. Serbyn, and A. F. Young, Half- and quarter-metals in rhombohedral trilayer graphene, Nature (London) **598**, 429 (2021), arXiv:2104.00653 [cond-mat.mes-hall].

[3] Y. Zhumagulov, D. Kochan, and J. Fabian, Emergent correlated phases in rhombohedral trilayer graphene induced by proximity spin-orbit and exchange coupling, arXiv e-prints , arXiv:2305.14277 (2023), arXiv:2305.14277 [cond-mat.str-el].

[4] S. Chatterjee, T. Wang, E. Berg, and M. P. Zaletel, Inter-valley coherent order and isospin fluctuation mediated superconductivity in rhombohedral trilayer graphene, Nature Communications **13**, 6013 (2022), arXiv:2109.00002 [cond-mat.supr-con].
