# Supplementary material for: Superconductivity from quasiparticle pairing of intervalley coherent state in rhombohedral trilayer graphene
Source: Nat Commun. 2026 May 28;17:6939. doi: 10.1038/s41467-026-72135-y (PMC13389250; doi:10.1038/s41467-026-72135-y)
Supplement: Supplementary file 2 — Transparent Peer Review file [file 41467_2026_72135_MOESM2_ESM.pdf]

nature portfolio

Peer Review File

# Superconductivity in Rhombohedral Trilayer Graphene: Quasiparticle Pairing within the Intervalley Coherent Phase

Corresponding Author: Dr Shuai Chen

**This file contains all reviewer reports in order by version, followed by all author rebuttals in order by version.**

Version 0:

Reviewer comments:

Reviewer #1

(Remarks to the Author)
In the paper with title "Superconductivity in Rhombohedral Trilayer Graphene: Quasiparticle Pairing within the Intervalley coherent Phase", Chau, Chen, and Law study the parameter dependence of the critical temperature, coherence length, and critical magnetic field in rhombohedral trilayer graphene. They compare the prediction of directly applying the mean-field approximation to the flavor-unpolarized normal state with experiment and find disagreements. Their proposal is that this can be resolved by taking a normal state with IVC order. This is studied in a toy model, which allows to derive analytic expressions, and numerically in a microscopic model.

I think that the underlying question is very important and timely. I also think that the structure of the manuscript is natural. However, before I can decide whether the paper should be published in an elite journal like Nature Communications, I would like the authors to address the following points:

1. While I agree that the red line in Fig. 2a does not capture the experimental Tc well, I am wondering whether it can also just be a consequence of the fact that the interaction entering the mean-field theory exhibits a strong filling-dependent renormalization (as e.g., would be captured within functional RG). I am not proposing to do such a calculation but I am wondering whether there is an intuitive reason why such a renormalization would not be consistent with the observed trend.

2. Could it be that the stiffness limits the experimental Tc rather than the mean-field Tc, at least in some parameter regime, and thus changing the theoretical line in Fig. 2a?

3. Just a minor aspect but what is the meaning of the scattered points in Fig. 2a, particularly prominent for low n? Would be good to comment on it to be self-contained. Relatedly a reader might be wondering what the experimental Tc curve looks like in Fig. 2a according to the authors.

4. I find the introduction of the toy model a bit confusing, upon first reading it. A reader might wonder when seeing Eq. (1): where is the IVC since the model seems entirely valley-diagonal. Would be good to improve the presentation a bit here.

5. What physical pairing mechanism do you have in mind? Phonons? Relatedly, would the results change significantly if the interaction in Eq. (2) would be not entirely local? I assume that this would change how the quantum geometry enters. Maybe at least a brief comment would be helpful.

6. Do I understand correctly that, apart from using an IVC normal state, which of course has different band structures etc., the calculation of all the superconducting properties just follows the usual mean-field/Ginzburg-Landau approach? If so, that's completely fine. I would just make that statement very clear, e.g., in the introduction and/or results section as it will help the theorist readers to understand the work faster. Or is the physics more like that of BEC vs. BCS? When you are at low filling, do you consider the fact that the superconducting order parameter changes the chemical potential?

Reviewer #2

(Remarks to the Author)

# Superconductivity in RTG

The manuscript proposes that the phenomenology of the superconductivity in RTG can be explained by a parent IVC states. It discusses the failure of the standard BCS theory applied to the flavor symmetric state, and claim that a parent IVC state is capable of explaining the experimental phenomenology better. While the technical derivations in the manuscript appears correct, I am confused by the presentation of the results, and the validity of the various approximations made. Therefore, I find that the manuscript as is it not suitable for publication at Nature Communications. A cleaner presentation that resolves the issues might make it suitable for Nat. Comm.

Below, I detail my understanding of the claims in the paper, together with my confusion about various steps in the paper.

1. The paper first summarizes the experimental phenomenology of the RTG, which hosts a variety of symmetry-broken states as well as superconducting states. The paper focuses mainly on SC1, which appears between the flavor symmetric phase and the PIP state, which the authors identify as the IVC state.

2. The authors then discuss whether the SC1 can be understood from a parent state with full flavor symmetry. To test this scenario, the authors perform some self-consistent calculation to extract U = 4meV. The detail of this process seems be rather scarce, and I don't quite understand which microscopic model is used for this particular calculation. Is it from linearized self-consistent equation, with v_f extracted from numerically computed bandstructure?

3. The authors also compute the coherence length from BCS relation for the single particle state, with hv_f for the graphene heterostructure. Again, how is this value computed? Is this the typical velocity at the outer and/or inner annulus?

4. The authors then try to build a toy model for the IVC phase as gapped Dirac cones. It is not clear to me why this is a good toy model. As the authors show themselves, the dispersion of the K-valley of rhombohedral graphene does not resemble that of a massive Dirac cone, because of the effects of trigonal warping etc. Why is it then relevant to take a massive Dirac cone, with its particular choice of spinors? Why should it be model as a massive Dirac cone, instead of an ordinary quadratic band dispersion, and does that affect the conclusion? I think these points need to be clarified much better.

5. Relatedly, I am unclear on the assumptions made about the behavior of the IVC state. For example, the authors claim that "The IVC phase (see Sec. Method) is characterized by two quasiparticle bands and transitions into the flavor symmetric state when the chemical potential starts to intersect at the top of the lower energy band." ([pdf](zotero://open-pdf/library/items/B2XALGT5?page=3)) What is the justification of this statement? It's not obvious to me that this is true, as the experimentally observed phase is partially-isospin polarized.

6. The authors consider mean-field theory of massive Dirac cones. The authors show that the transition temperature has a different dependence on \rho_QP then the standard dependence, with the following justification: "Note that there is an extra factor of two in the exponential term in comparison with the BCS limit due to the integral range e"ectively halved." ([pdf](zotero://open-pdf/library/items/B2XALGT5?page=3)) I do not understand this justification. Can you perhaps point to the part of the manuscript that makes this fact clearer? Also, \epsilon_D should be define earlier, as the authors write \epsilon_D > \mu before defining \epsilon_D.

7. Relatedly, the authors consider the regime where \mu < \epsilon_D and also \beta_MF |\mu - m| <<1. The former of course corresponds to the antiadabatic limit. However, doesn't the latter make is a strongly-coupled state?

8. The authors also show that the coherence length is scaling differently then BCS. Does this have anything to do with the Dirac nature of the electrons, or simply a result of being at low density compared to \epsilon_D (i.e. antiadiabatic limit).

9. The authors now apply this model to SC in RTG. The mapping between the parameters in the massive Dirac cone to that in the actual RTG is not made very clear. The value of massgape = 34.5 meV is quoted, which seems to be consistent with the band structure in 2(d), but I am not sure about my interpretation. I believe this paragraph needs to be rewritten much more clearly.

10. The authors then perform numerical calculations on the microscopic model. The computation for \Delta_IVC is a bit opaque to me. Are you setting \Delta_IVC based on whether the normal state dopes into the blue band or not? The authors then say Eq. (17) is used to compute the transition temperature, but Eq. (17) is derived in the context of the the toy model in the Methods section. Is the claim that the same formula is applicable for the microscopic calculation?

11. The authors claim that H_C2 is significantly modified by the quantum metric effect. I am not sure if the experimental phenomenology is rich enough to detect this effect. At B=0, as we tune n, we can see a phase transition from SC to a metal. Within GL picture, this point corresponds to when the coefficient of the quadratic term is zero, thus signaling divergence coherence length. Therefore, the phase boundary seems to give divergent coherence length even in the absence of quantum metric contribution. Unless there is an extended regime in which coherence length is infinite, I find it difficult to distinguish the above scenario from quantum metric driven scenario.

Here are some miscellaneous comments

1. In method, the density of states of the quasi particles are said to "peak at the bottom of the conduction band", but isn't it the

valence band?

Overall, I believe the manuscript contains a physically novel idea, but it suffers from somewhat cluttered presentation mixed with assumptions whose justifications are not made very clear. I encourage the authors to address the comments above in order to make a stronger case for a publication at Nature Communications.

Version 1:

Reviewer comments:

Reviewer #1

(Remarks to the Author)
The authors have addressed all of my comments and revised the manuscript accordingly. The current version can be published in my opinion.

Reviewer #2

(Remarks to the Author)
Review of “Superconductivity in Rhombohedral Trilayer Graphene: Quasiparticle Pairing within the Intervalley Coherent Phase”

I thank the authors for a thorough revision. However, while the paper has significantly improved in readability, I am still not sure if this paper is suitable for a publication at Nature Communications.

The fundamental issue I have with the paper is that different levels of approximations are relatively freely mixed together, making it hard to understand the strength of each computation.

For example, in an early section, an estimate of coherence length is given by using the “average” fermi velocity of graphene materials. However, tunneling between layers is known to modify band dispersion drastically. It is unclear to me why the approximating the fermi velocity by the value obtained from graphene linear dispersion is appropriate.

It is also not clear to me why a massive Dirac cone is an appropriate toy model for the phenomenology of IVC-based mechanism. Is the fact it’s a Dirac cone important at all? It looks as though a lot of physics can be recovered by having normal quadratic bands whose band edge appears at a slightly offset chemical potential. There is an extensive discussion of quantum metric contribution to coherence length, but it’s relevance to experimental data is unclear at best, given the minimal effect it has on the phase boundary in fig.3(b). Additionally, even if quantum metric were important, the paper does not really justify why the quantum metric of a Dirac cone is a better approximation for the band edge than a simple quadratic model.

Overall, while the proposed mechanism is interesting, the theoretical analysis is not carried out at the level of clarity and rigor required for a publication at Nature communications.

Here are point by point comments/

1. “Instead, we propose that when in close vicinity with the phase transition from IVC to a flavor symmetric state, where the chemical potential cuts the band edge of the lower energy IVC quasiparticle band, would increase in density of state thus favoring direct pairing between IVC quasiparticles, where the IVC order parameter remains intact.” this seems to have subject-object mismatch.

2. “Focusing on SC1, at n ↔ ↓1.85 ↗ 1012cm↓2, corresponding to chemical potential of μ ↔ ↓43.4meV, the temperature is maximum, at TMF ↔ 120mK.” you probably meant to say something like “the transition temperature takes the maximum value”. Also, T_MF is probably supposed to be T_c, since it’s not a mean-field value.

3. “Since only quasiparticles from the edge of lower energy IVC band majorly contribute to the formation of Cooper pairs, the lack of states within the band gap is well captured by the proposed toy model.” The use of the word “band gap” is confusing in this context, since there is still finite density of states arising from the other band. I think a better terminology is necessary here.

4. “Using the fact that at charge career density …… we can deduce \Delta_IVC = 7 meV”. This was not clear to me. Is it obtained from some fitting procedure?

5. “The mean-field temperature calculated from the IVC state, in general, agrees with the experimental result, both reaching a maximum of at” Remove second to last “of”

Version 2:

Reviewer comments:

Reviewer #1

(Remarks to the Author)
In my opinion all comments raised have now been successfully addressed in the response and taken into account in the final version of the manuscript. Although the question of whether the degree of relevance/significance of a work is sufficient for publication in a high-impact journal like Nature Communications is always subjective, I think that publication is justified in the current case.

Reviewer #2

(Remarks to the Author)
The authors have provided important clarifications in response to my questions, and I thank them for the work they put in to improve the manuscript. I can recommend its publication to Nature Communication.

**Open Access** This Peer Review File is licensed under a Creative Commons Attribution 4.0 International License, which permits use, sharing, adaptation, distribution and reproduction in any medium or format, as long as you give appropriate credit to the original author(s) and the source, provide a link to the Creative Commons license, and indicate if changes were made.
In cases where reviewers are anonymous, credit should be given to 'Anonymous Referee' and the source.
The images or other third party material in this Peer Review File are included in the article's Creative Commons license, unless indicated otherwise in a credit line to the material. If material is not included in the article's Creative Commons license and your intended use is not permitted by statutory regulation or exceeds the permitted use, you will need to obtain permission directly from the copyright holder.
To view a copy of this license, visit https://creativecommons.org/licenses/by/4.0/

**Main revisions**

*(i) we addressed why experimental $T_c$ cannot be captured by superconductivity originating from flavor symmetric phase, even if additional factors such as stiffness is considered, following the suggestion of Reviewer 1.*

*(ii) we revised Fig. 2a, 3a and 3b to remove noise due to data extraction, and added experimental Tc curves to Fig. 2a according to suggestion of Reviewer 1.*

*(iii) we revised the presentation of the toy model following suggestions of Reviewers 1 and 2, i.e. justify the toy model is suitable for describing superconductivity via quasiparticle pairing in in a IVC phase*

*(iv) we extended the discussion of superconductivity occurring beyond band edge, hinting at possible BEC/strongly-correlated regime, following comments of Reviewers 1 and 2.*

*(v) we provided details on computation of physical quantities, for example mean-field temperature, from microscopic model, upon request of Reviewer 2.*

*(vi) we clarified properties of the IVC phase, for example physics behind phase transition to flavor symmetric phase, following comments of Reviewers 2.*

*(vii) we revised argument for the unique mean-field temperature dependency of band edge superconductivity, upon request of Reviewer 2.*

*(viii) we justified Eqs. (17-22) remains valid beyond proposed toy model, and is applicable to superconductivity observed in RTG, upon request of Reviewer 2.*

*(ix) we changed the term "band bottom" to "band edge" for clarity.*

## Reviewer 1

*In the paper with title "Superconductivity in Rhombohedral Trilayer Graphene: Quasiparticle Pairing within the Intervalley coherent Phase", Chau, Chen, and Law study the parameter dependence of the critical temperature, coherence length, and critical magnetic field in rhombohedral trilayer graphene. They compare the prediction of directly applying the mean-field approximation to the flavor-unpolarized normal state with experiment and find disagreements. Their proposal is that this can be resolved by taking a normal state with IVC order. This is studied in a toy model, which allows to derive analytic expressions, and numerically in a microscopic model.*

We thank the Referee for their precious time dedicated to reviewing our manuscript and for their constructive feedback.

*I think that the underlying question is very important and timely. I also think that the structure of the manuscript is natural. However, before I can decide whether the paper should be published in an elite journal like Nature Communications, I would like the authors to address the following points:*

We thank the Referee for positive feedback and recognizing the importance of the question we have studied. We have revised our manuscript according to the constructive comments and suggestions of the first Referee.

*1. While I agree that the red line in Fig. 2a does not capture the experimental $T_c$ well, I am wondering whether it can also just be a consequence of the fact that the interaction entering the mean-field theory exhibits a strong filling-dependent renormalization (as e.g., would be captured within functional RG). I am not proposing to do such a calculation, but I am wondering whether there is an intuitive reason why such a renormalization would not be consistent with the observed trend.*

**Our response:** We thank the Referee for this point. We agree with the referee that a filling-dependent renormalization can be a possible explanation on the superconducting dome. Regarding the region of the SC1, we can argue that such a renormalization may not be relevant. Above all, strong filling-dependent renormalization of electronic interactions fundamentally relies on significant changes in the density of states (DoS), such as those introduced by van Hove singularities or Fermi surface topology transitions. These features amplify electronic susceptibilities and interaction effects. However, as shown in Fig. 1, within the charge carrier density where SC is observed, if only flavor symmetric phase is considered the DoS varies smoothly (at doping $n \approx -1.93 \times 10^{12}\text{cm}^{-2}$ to $-1.8 \times 10^{12}\text{cm}^{-2}$, see Fig. 1) which is

away from the van Hove singularity (at doping $n \approx -0.5 \times 10^{12}\mathrm{cm}^{-2}$). Furthermore, there are no surface topology transitions within the regime where SC1 is observed. Therefore, we may not expect a strong filling-dependent renormalization.

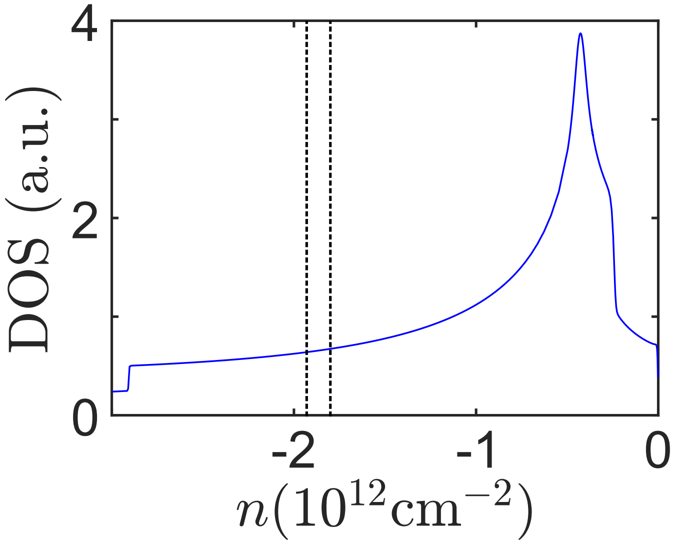


**Figure 1.** Density of state (DOS) as function of charge carrier density, with the dashed line indicating the regime where superconductivity is observed in experiment. This regime lies well away from the van Hove singularity, and thus the DOS varies smoothly.

We do comment that using functional RG [PRL. 130, 146001 (2022)], it is possible to qualitatively retrieve a superconducting dome observed in experiment, under the condition of an inter-Fermi-surface nesting.

*2. Could it be that the stiffness limits the experimental $T_c$ rather than the mean-field $T_c$, at least in some parameter regime, and thus changing the theoretical line in Fig. 2a?*

**Our response:** We thank the Referee for this point. For the setup of our manuscript, we can approximate the superfluid weight from the band dispersion as $D_{ij} \sim -\sum_{\mu<\varepsilon(\mathbf{k})} \partial_i \partial_j \varepsilon(\mathbf{k})$, where $\varepsilon(\mathbf{k})$ is the band dispersion of the normal phase as the quantum metric contribution can be ignored. In Fig. 2, we plot $\sqrt{\det(D_{ij})} = \sqrt{D_{xx}D_{yy} - D_{xy}^2}$ for the flavor symmetric phase with the charge carrier density region where SC is observed where the RTG band dispersion $\varepsilon(\mathbf{k})$ is obtained from a six-band model (see Phys. Rev. B 82, 035409 (2010) or Sec. I in the Supplementaru Material in our manuscript). The BKT transition temperature can be determined as $k_B T_{\mathrm{BKT}} = \frac{\pi}{8}\sqrt{\det(D_{ij}(T_{\mathrm{BKT}}))}$. Thus, approximately, a higher value $\sqrt{\det(D_{ij})}$ for $\Delta_0 = 0$ corresponds to a higher $T_{\mathrm{BKT}}$, implying that the trend of the BKT transition temperature follows that of the mean-field critical temperature in the flavor-symmetric phase. As a result, this stiffness-limited transition fails to reproduce the superconducting dome observed experimentally.

*3. Just a minor aspect but what is the meaning of the scattered points in Fig. 2a, particularly prominent*

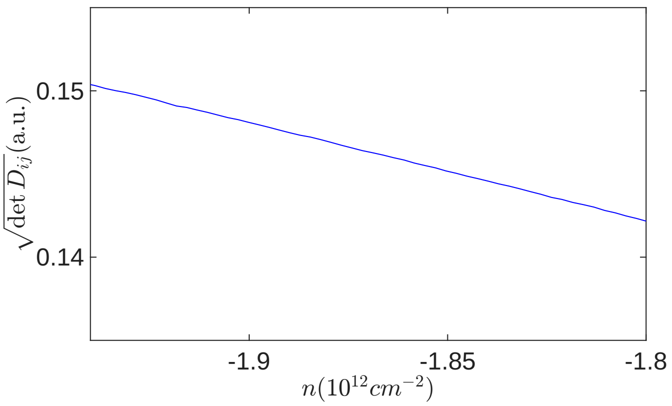


**Figure 2.** Superfluid stiffness as function of charge carrier density when $\Delta_0 = 0$ by using $D_{ij} \sim -\sum_{\mu<\varepsilon(\mathbf{k})} \partial_i \partial_j \varepsilon(\mathbf{k})$ where the band dispersion $\varepsilon(\mathbf{k})$ of the normal phase is calculated from the 6-band model.

*for low n? Would be good to comment on it to be self-contained. Relatedly a reader might be wondering what the experimental Tc curve looks like in Fig. 2a according to the authors.*

**Our response:** We thank the Referee for their point. The blue region in Fig. 2a in the manuscript corresponds to experimental data from [Nature (London) 598, 434–438 (2021)], where the resistance is smaller than a threshold value $\sim 10\Omega$. The scattered pointed in Fig. 2a in the manuscript is due to the noise from the data extraction. In the revised version, we have chosen the threshold value such that the superconducting dome is clearly shown, meanwhile removing scattered points outside the superconducting dome. We also added a green dash line to Fig. 2a to highlight the experimental $T_c$ curve from our viewpoint. Fig. 3a and 3b in the manuscript are also edited accordingly to remove noise from data extraction for consistency.

*4. I find the introduction of the toy model a bit confusing, upon first reading it. A reader might wonder when seeing Eq. (1): where is the IVC since the model seems entirely valley-diagonal. Would be good to improve the presentation a bit here.*

**Our response:** We thank Referee for the suggestion on improvement of our manuscript. In the revised version, we have added more details of the toy model.

In our toy model,we avoid the complicated band structure of RTG by considering two Dirac cones with Fermi velocity $v$, centered at the valleys $K$ and $K'$, respectively. The Dirac cones are gapped with a mass gap $m$ due to the external potential, for example, a displacement field. The IVC order we consider is the time-reversal symmetric density wave. Effectively we can use a continuous model $H = H_0 + V_{\text{IVC}}$. $H_0$

captures the band structure without IVC order:

$$H_0 = \sum_{s\tau}\sum_{\mathbf{q}} \psi^\dagger_{\tau s}(\mathbf{q}) h_\tau(\mathbf{q}) \psi_{\tau s}(\mathbf{q}), \tag{1}$$

with the spinor $\psi_{\tau s} = [a_{\tau s A}, a_{\tau s B}]^T$ at two sublattices $\lambda = A, B$ given the spin index $s = \uparrow, \downarrow$ and the valley index $\tau = \pm$. The matrix $h_\tau(\mathbf{q})$ describes a massive Dirac cone centered at valley $\tau$:

$$h_\tau(\mathbf{q}) = v\mathbf{q} \cdot (\tau\sigma_x \hat{\mathbf{e}}_x + \sigma_y \hat{\mathbf{e}}_y) + m\sigma_z, \tag{2}$$

where $\sigma_{x,y,z}$ correspond to the Pauli matrix defined in the sublattice basis. After diagonalizing $H_0$, we have two bands with $H_0 = \sqrt{v^2q^2+m^2}(a^\dagger_{1\tau s}(\mathbf{q})a_{1\tau s}(\mathbf{q}) - a^\dagger_{2\tau s}(\mathbf{q})a_{2\tau s}(\mathbf{q}))$. In particular, we introduce an IVC order through $V_{\text{IVC}}$,

$$V_{\text{IVC}} = \sum_s \psi^\dagger_{+s}(\mathbf{q}) \hat{\Delta}_{\text{IVC}}(\mathbf{q}) \psi_{-s}(\mathbf{q}) + h.c., \tag{3}$$

where $\hat{\Delta}_{\text{IVC}}(\mathbf{q})$ denotes real and uniform order parameter $\Delta_{\text{IVC}}$ when formulating in the band basis: $V_{\text{IVC}} = \sum_s \sum_{\mathbf{q}} \Delta_{\text{IVC}}[a^\dagger_{1+s}(\mathbf{q})a_{1-s}(\mathbf{q}) + a^\dagger_{2+s}(\mathbf{q})a_{2-s}(\mathbf{q})] + h.c.$. There are four quasiparticle bands in the toy model given the spin index. We focus on the quasiparticle band of the dispersion spectrum $E(\mathbf{q}) = -\sqrt{v^2q^2+m^2} - \Delta_{\text{IVC}}$, and consider only s-wave on-site attractive interaction:

$$H_{\text{int}} = -U \sum_{s\lambda\lambda'} \int d^2\mathbf{r}\, a^\dagger_{+s\lambda}(\mathbf{r}) a^\dagger_{-\bar{s}\lambda}(\mathbf{r}) a_{-\bar{s}\lambda'}(\mathbf{r}) a_{+s\lambda'}(\mathbf{r}), \tag{4}$$

where $U$ is the attractive interaction strength and $\lambda = A, B$ denote the sublattices. We have used $\bar{s}$ to indicate the opposite spin. In our setup, we only consider an s-wave pairing. We do not exclude the possibility of an unconventional superconducting order. A key aspect of our analysis is that the presence of IVC order can lift the valley degeneracy, thereby shifting the chemical potential toward the band edge—this shift forms the foundation of our theoretical framework.

In the revised version, we have rewritten the corresponding section. We thank the referee once again for the constructive comments.

*5. What physical pairing mechanism do you have in mind? Phonons? Relatedly, would the results change significantly if the interaction in Eq. (2) would be not entirely local? I assume that this would change how the quantum geometry enters. Maybe at least a brief comment would be helpful.*

**Our response:** The Coulomb mediated interaction is a likely candidate behind the physical pairing mechanism, since the strength of the phonon-mediated interaction is relatively small. As in Ref. [Phys. Rev. B 106, 155115 (2022)], an effective attraction between quasiparticles can emerge from the renormalization

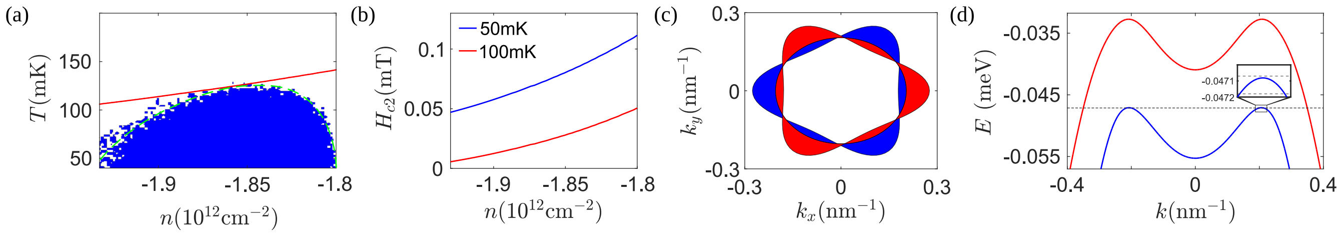


**Figure 3. Updated Fig. 2** in the main text: predictions from the theory by assuming SC1 from flavor-symmetric phase. (a) The mean-field transition temperature (red curve) by assuming superconductivity from a flavor-symmetric phase. The blue region is extracted for SC1 from the experiment in Ref. 1, and green dashed line indicates the corresponding transition temperature. The predictions deviate from the experimental measurements in particular at the phase boundary $n = -1.8 \times 10^{12}\mathrm{cm}^{-2}$.

of the Coulomb interaction.

On the other hand, a long-range interaction will not change the physical picture of the quasiparticle pairing. In Ref. [Nature Communications 13, 6013 (2022)], a non-local interaction such as screened coulomb interaction could introduce higher-order pairing, such as d-wave or f-wave superconductivity. Of course, some quantitative change can occur on the physical quantities. We take the coherence length as an example. In Ref. [Phys. Rev. Lett. 132, 026002 (2024)] (co-authored by two of us), a general formula is demonstrated for the superconducting coherence length in a dispersion band of finite quantum geometry: $\xi_{\mathrm{sc}} = \sqrt{\xi_{\mathrm{BCS}}^2 + \xi_{\mathrm{qg}}^2}$ with $\xi_{\mathrm{BCS}}$ ($\xi_{\mathrm{qg}}$) contributed from the BCS relation (quantum geometry). For an s-wave pairing, a long-range interaction can lead to a spatially-extended Cooper pair, giving rise to an enlarged $\xi_{\mathrm{BCS}}$. The quantum metric contribution $\xi_{\mathrm{qg}} = \sqrt[4]{\det \bar{g}(\mathbf{k})}$ with $\bar{g}_{ab}(\mathbf{k}) = (\sum_{\mathbf{k}} \frac{g_{ab}(\mathbf{k})}{E(\mathbf{k})})/(\sum_{\mathbf{k}} \frac{1}{E(\mathbf{k})})$ depends on the SC quasiparticle dispersion $E_k$ and normal-state quantum geometry $g_{ab}(\mathbf{k})$. As long as there is no significant change on quasiparticle dispersion $E(\mathbf{k})$ from the long-range interaction, one may not expect significance change of the $\xi_{\mathrm{qg}}(\mathbf{k})$.

We briefly comment on the possibility of higher-order pairing given non-local interaction after Eq. (5) in the revised version (previously Eq. (2)).

*6. Do I understand correctly that, apart from using an IVC normal state, which of course has different band structures etc., the calculation of all the superconducting properties just follows the usual mean-field/Ginzburg-Landau approach? If so, that's completely fine. I would just make that statement very clear, e.g., in the introduction and/or results section as it will help the theorist readers to understand the work faster. Or is the physics more like that of BEC vs. BCS? When you are at low filling, do you consider the*

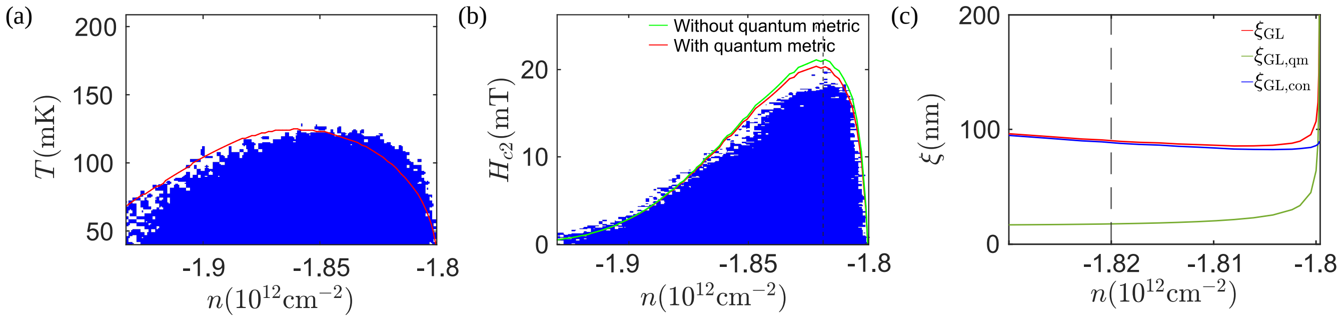


**Figure 4. Updated Fig. 3** in the main text: predictions from the theory by assuming SC1 originates from pairing of quasiparticles in IVC phase: (a) mean-field transition temperature, (b) upper critical field $H_{c2}$ at $T = 50$mK, and (c) the Ginzburg-Landau coherence lengths at $n \approx -1.82 \times 10^{12}\text{cm}^{-2}$.

*fact that the superconducting order parameter changes the chemical potential?*

**Our response:** We thank the Referee for this point. The Referee is correct. Our calculation follows from the mean-field/Ginzburg-Landau approach for studying superconductivity, with two additional considerations: 1). Effect of quantum geometry, in particular in coherence length, first discussed in [Phys. Rev. Lett. 132, 026002 (2024)]. 2). Chemical potential near band edge. Superconductivity can be observed even when the charge carrier density is beyond the band edge at finite temperature, hinting at possible BEC physics in this specific regime. To simplify the discussion, we emphasize the physics in most regime can be understood sufficiently with BCS. To understand the transition temperature measured in experiments, we use the pairing breaking temperature, that is, the mean-field transition temperature. In contrast, from a BEC picture, the transition temperature is controlled by the BKT temperature and there would be a pseudogap phase which may also influence the measured transition temperature. As we argued in the point 2, in our setup, the BKT transition shares the same tendency as the mean-field transition temperature.

In our calculations, we define the chemical potential from the number equation, that is given by $n = \int d^2\mathbf{r} \sum_{\tau s\lambda} \langle a^\dagger_{\tau s\lambda}(\mathbf{r}) a_{\tau s\lambda}(\mathbf{r})\rangle$ with $n$ being the charge carrier density. We derive the Ginzburg-Landau theory when the temperature is close to the critical temperature $T_c$, the chemical potential $\mu$ now does not depend on the mean-field value of the order parameter which is assumed to vanish around the critical temperature. At general temperature, the chemical potential is indeed affected by the superconducting order parameter, which we have already taken into account when solving the mean-field gap equation.

## Reviewer 2

*The manuscript proposes that the phenomenology of the superconductivity in RTG can be explained by a parent IVC states. It discusses the failure of the standard BCS theory applied to the flavor symmetric state, and claim that a parent IVC state is capable of explaining the experimental phenomenology better. While the technical derivations in the manuscript appears correct, I am confused by the presentation of the results, and the validity of the various approximations made. Therefore, I find that the manuscript as is it not suitable for publication at Nature Communications. A cleaner presentation that resolves the issues might make it suitable for Nat. Comm.*

**Our response:** We thank the Referee for their precious time dedicated to reviewing our manuscript and for their constructive feedback.

*Below, I detail my understanding of the claims in the paper, together with my confusion about various steps in the paper.*

**Our response:** We thank the Referee for their detailed comments. Below we address Referee's questions and comments in a point-by-point manner.

*1. The paper first summarizes the experimental phenomenology of the RTG, which hosts a variety of symmetry-broken states as well as superconducting states. The paper focuses mainly on SC1, which appears between the flavor symmetric phase and the PIP state, which the authors identify as the IVC state.*

**Our response:** We thank Referee for the summary. Indeed, we first summarized experimental observations of SC in the RTG in [Nature (London) 598, 434–438 (2021)], which aims at providing readers that are not familiar with the RTG with background information to understand our paper. The PIP state, which is observed adjacent to the SC1, have previously been speculated as the IVC state in [Phys. Rev. B 105, 134524 (2022)] and [Nature Communications 13, 6013 (2022)].

*2. The authors then discuss whether the SC1 can be understood from a parent state with full flavor symmetry. To test this scenario, the authors perform some self-consistent calculation to extract* $U = 4meV$*. The detail of this process seems be rather scarce, and I don't quite understand which microscopic model is used for this particular calculation. Is it from linearized self-consistent equation, with* $v_f$ *extracted from numerically computed band structure?*

**Our response:** We apologize for any potential confusion caused in the current manuscript and thank the

Referee for the opportunity to clarify our description. When calculating the mean-field temperature, we use the linearized gap equation as the form in Eq. (20) (previously Eq. (17)) in the manuscript. More explicitly, we use the 6-band Hamiltonian to mimic the band structure of RTG [Phys. Rev. B 82, 035409 (2010)] which is detailed in Sec. I in Supplementary Material. We also consider the intervalley pairing with the attractive interaction

$$H_{\text{int}} = -U \sum_{s} \sum_{\lambda\lambda'} \int d^2\mathbf{r}\, a^{\dagger}_{+s\lambda}(\mathbf{r}) a^{\dagger}_{-\bar{s}\lambda'}(\mathbf{r}) a_{-\bar{s}\lambda'}(\mathbf{r}) a_{+s\lambda}(\mathbf{r}), \tag{5}$$

where $\pm$ are valley indices, $s = \uparrow, \downarrow$ are the spin indices, and $\lambda, \lambda' = A, B$ are two sublattices. Also, we used $\bar{s}$ to denote the opposite spin. By assuming an s-wave pairing $\Delta = 1/N \sum_{\mathbf{q}} \sum_{s} \sum_{\lambda} \langle a_{+s\lambda}(\mathbf{q}) a_{-\bar{s}\lambda}(-\mathbf{q}) \rangle$, the linearize gap equation takes the form as

$$1 = U \int \frac{d^2\mathbf{q}}{(2\pi)^2} \frac{1}{2\varepsilon(\mathbf{q})} \tanh \frac{\beta_{\text{MF}} \varepsilon(\mathbf{q})}{2}, \tag{6}$$

where we only choose the dispersion $\varepsilon(\mathbf{q})$ of the valence band at Fermi energy as there is a large band gap separating the conduction and valence bands. Thus there is no need to use $v_f$ extracted from the band structure. The attractive interaction strength $U = 4$meV in Eq. (5) is set such that the fit matches the maximal $T_c \sim 120$mK at the charge carrier density $n = -1.85 \times 10^{12}\text{cm}^{-2}$. With the fitted $U = 4$meV, we find that the superconducting dome cannot be reproduced between the fermions with full flavor symmetry without considering an IVC order. In the revised version, we have rewritten the description correspondingly:

> By fitting the maximum transition temperature at the specific charge carrier density, we estimate an attractive interaction strength of approximately $U \approx 4$eV using the linearized gap equation Eq. (20), as discussed in detail in Sec. Method. In specific, the dispersion spectrum is extracted from the 6-band Hamiltonian for RTG [Phys. Rev. B 82, 035409 (2010)] detailed in Sec. I in Supplementary Material.

*3. The authors also compute the coherence length from BCS relation for the single particle state, with $hv_f$ for the graphene heterostructure. Again, how is this value computed? Is this the typical velocity at the outer and/or inner annulus?*

**Our response:** We thank the Referee for this point. $\hbar v_F$ refers to the typical velocity of graphene heterostructure, namely the fermi velocity of massless graphene monolayer, not the fermi velocity (group velocity) $\nabla_{\mathbf{k}}\varepsilon(\mathbf{k})$ at the fermi surface. For clarity, we change the symbol to $v$ instead of $v_F$, to indicate it is not the fermi velocity at the given charge carrier density. The value is extracted from tight-binding parameters for RTG [Nature (London) 598, 434–438 (2021)], which is also listed in Table 1

of Supplementary Material. To be precise, it is determined by $\hbar v = \sqrt{3}a\gamma_0/2$, where $a = 0.246$nm, and $\gamma_0 = 3.1$eV, which is the nearest-neighbor hopping strength within individual layer of graphene.
In the revised manuscript, we write there correspondingly:

> At charge carrier density $n \approx -1.85 \times 10^{12}\text{cm}^{-2}$, where the transition temperature is maximum, under BCS relation [37], the coherence length can be approximated by $\xi_0 \sim 0.18\hbar v/k_B T_c$, with $\hbar v \approx 6.6 \times 10^{-1}$eVnm being the typical velocity of graphene heterostructure, defined by the fermi velocity of massless graphene monolayer.

*4. The authors then try to build a toy model for the IVC phase as gapped Dirac cones. It is not clear to me why this is a good toy model. As the authors show themselves, the dispersion of the K-valley of rhombohedral graphene does not resemble that of a massive Dirac cone, because of the effects of trigonal warping etc. Why is it then relevant to take a massive Dirac cone, with its particular choice of spinors? Why should it be model as a massive Dirac cone, instead of an ordinary quadratic band dispersion, and does that affect the conclusion? I think these points need to be clarified much better.*

**Our response:** We thank the Referee for this point. We choose to use massive Dirac cones for two main reasons. First, they serve as a minimal model that captures the dispersion and the Bloch wave structure of the quasiparticles near the band edge in IVC phase. Second, this choice is physically motivated by the graphene-based heterostructures under consideration, where an IVC order parameter is essential for the band edge superconductor. Without an IVC order, the chemical potential within the superconducting regime is indeed no longer close to the band edge. Hence, as shown in Fig. 2(a), the fitted critical temperature fails to cover the experimental measurement by assuming pairing of a flavor-symmetric phase. We also note the particular choice of spinors in massive Dirac cone, naturally gives rise to quantum geometry, manifested as the quantum metric, which peaks at the band edge. This is similar to how quantum geometry varies as a function of charge carrier density in RTG. Thus, the toy model of gapped Dirac cones, qualitatively have similar tendency in quasiparticle energy and quantum geometry with IVC states, can give predictions that can capture the key features of a numerical calculation on a microscopic model.

While we agree that near the band edge the dispersion can be approximated by a quadratic form, it becomes linear when the chemical potential moves away from the band gap—an effect that a simple quadratic model cannot capture. For the charge carrier density range relevant to the experiment, the physical properties are not significantly affected by assuming a conventional quadratic band. As shown in Fig. 3(b) in the manuscript, the quantum metric contribution to the upper critical field $H_{c2}$ is negligible. However, close to the phase boundary between the IVC-SC and IVC phases, the quantum metric contribution can become

significant. In this regime, quadratic bands fail to offer a reliable description of the system as there is no quantum geometry in a single ordinary quadratic band.
We also comment for the IVC state, the dispersion spectrum is given by Eq. (12) (previously Eq. (11)) in the manuscript:

$$\varepsilon_{\pm}(\mathbf{q}) = \varepsilon_s(\mathbf{q}) \pm |\Delta(\mathbf{q})|, \tag{7}$$

where $\varepsilon_s = -\mu + \frac{1}{2}[\varepsilon_K(\mathbf{q}) + \varepsilon_{K'}(\mathbf{q})]$ is the symmetric part of the flavor-symmetric state. We note the effect of trigonal warping is insignificant as only the symmetric part contributes, and the quasiparticle dispersion can be treated as centrosymmetric, which is illustrated in Fig. 2(d) in the manuscript.

*5. Relatedly, I am unclear on the assumptions made about the behavior of the IVC state. For example, the authors claim that "The IVC phase (see Sec. Method) is characterized by two quasiparticle bands and transitions into the flavor symmetric state when the chemical potential starts to intersect at the top of the lower energy band." ([pdf](zotero://open-pdf/library/items/B2XALGT5?page=3)) What is the justification of this statement? It's not obvious to me that this is true, as the experimentally observed phase is partially-isospin polarize.*

**Our response:** We thank the Referee for this point. Experimentally [Nature (London) 598, 434–438 (2021)], and later in [Nature Physics 20, 1413–1420 (2024)] the identity of the PIP state near SC1 remains unclear, but is compatible with IVC given the existing symmetry constraints from measurements.

From a theoretical standpoint, as discussed in [Phys. Rev. B 105, 134524 (2022)], the Fermi surfaces at van Hove singularities are well nested between $K$ and $K'$ valleys, which provides a possible mechanism for a strong instability towards IVC under the repulsive interaction.
The same argument can be extended to the phase transition from IVC state into the flavor symmetric state. As the chemical potential crosses the top of the lower energy quasiparticle band (colored blue in Fig. 2d in the manuscript), it results in the increasing a discrete jump in the DoS. The IVC state is as such no longer energetically favorable, resulting in transition to flavor symmetric phase. To clarify, we simplify the statement in the revised manuscript:

> The IVC-SC phase is observed in the vicinity of the phase boundary in between the IVC and the flavor-symmetric state, at which the chemical potential cuts the band top of the lower energy IVC band (colored blue in Fig. 2d). This results in a discrete jump in DoS for the IVC quasiparticle, and leads to strong instability towards flavor symmetric state.

*6. The authors consider mean-field theory of massive Dirac cones. The authors show that the transition temperature has a different dependence on $\rho_{QP}$ than the standard dependence, with the following justification: "Note that there is an extra factor of two in the exponential term in comparison with the BCS limit*

*due to the integral range effectively halved." ([pdf](zotero://open-pdf/library/items/B2XALGT5?page=3)) I do not understand this justification. Can you perhaps point to the part of the manuscript that makes this fact clearer? Also, $\varepsilon_D$ should be define earlier, as the authors write $\varepsilon_D > \mu$ before defining $\varepsilon_D$.*

**Our response:** we thank the Referee for this point. In the BCS limit, the self-consistent equation for any centrosymmetric model is given by:

$$\begin{aligned}\frac{1}{U} &= \int \frac{d^2\mathbf{q}}{(2\pi)^2}\frac{1}{2\varepsilon(\mathbf{q})}\tanh\frac{\beta_{\text{MF}}\varepsilon(\mathbf{q})}{2} \\ &= \int_{-\varepsilon_D}^{\varepsilon_D}\frac{d\varepsilon}{2\pi}\rho(\varepsilon)\frac{1}{2\varepsilon}\tanh\frac{\beta_{\text{MF}}\varepsilon}{2}. \end{aligned} \tag{8}$$

But at the band edge, since no states exists below the band edge (within the gap), the self-considtent equation is modified as:

$$\frac{1}{U} = \int_{0}^{\varepsilon_D}\frac{d\varepsilon}{2\pi}\rho(\varepsilon)\frac{1}{2\varepsilon}\tanh\frac{\beta_{\text{MF}}\varepsilon}{2}, \tag{9}$$

where the integral range is now reduced to half of the BCS limit. Halving the integral range for the toy model lead to extra factor of two in the exponential term, detailed in Supplementary Note Sec. IV.
In the revise manuscript, we have justified as follows:

> Note that there is an extra factor of two in the exponential term in comparison with the BCS limit, as no states exist within the band gap, effectively halving the integral range over energy when calculating mean-field temperature self-consistently.

We have also defined $\varepsilon_D$ earlier, when we write $\mu < \varepsilon_D$ for clarity:

> We focus on the case where chemical potential $\mu < \varepsilon_D$, which is the relevant regime to experiments, with $\varepsilon_D$ represents the Debye energy for the attractive interaction.

*7. Relatedly, the authors consider the regime where $\mu < \varepsilon_D$ and also $\beta_{MF}|\mu - m| \ll 1$. The former of course corresponds to the antiadiabatic limit. However, doesn't the latter make is a strongly-coupled state?*

**Our response:** We thank the Referee for this point. The latter case $\beta_{MF}|\mu - m| \ll 1$ corresponds to the chemical potential lying near the band edge, and we agree that this corresponds to a strongly-coupled state, for example a BEC state. What we aim at in the current manuscript is to find the relation between IVC and SC1. Thus, we make the simple assumption of the BCS superconducting state while our calculations are in agreement with the experiment given the SC originates from the IVC state.

*8. The authors also show that the coherence length is scaling differently than BCS. Does this have anything to do with the Dirac nature of the electrons, or simply a result of being at low density compared to $\varepsilon_D$ (i.e.*

*antiadiabatic limit).*

**Our response:** we thank the Referee for this point. The total coherence length has two contributions from both band dispersion ($\xi_{\rm con}$) and the quantum metric ($\xi_{\rm qp}$). The coherence length contribution $\xi_{\rm con}$ depends solely on the band dispersion. In particular, as the IVC-SC occurs in the vicinity of the band edge, we have the scaling behavior $\xi_{\rm conv} \sim m_{\rm eff}/\sqrt{T_{\rm MF}}$, where $m_{\rm eff}$ is the effective mass at the band edge. However, the quantum metric contribution $\xi_{\rm qp}$, cannot be naturally introduced, solely by the dispersion, as discussed previously regarding the role of spinors within the toy model. We also comment that the scaling behavior $\xi_{\rm conv} \sim m_{\rm eff}/\sqrt{T_{\rm MF}}$ is not general for the antiadiabatic limit. For example, if the dispersion is of higher order, for example quartic $\varepsilon(\mathbf{k}) \propto k^4$, which can result in divergent DoS at the band edge, we expect the coherence length scaling to be different from both BCS and our result.

*9. The authors now apply this model to SC in RTG. The mapping between the parameters in the massive Dirac cone to that in the actual RTG is not made very clear. The value of mass gap $e = 34.5meV$ is quoted, which seems to be consistent with the band structure in 2(d), but I am not sure about my interpretation. I believe this paragraph needs to be rewritten much more clearly.*

**Our response:** We thank the Referee for this point. The purpose of this paragraph is to qualitatively infer prediction on the IVC-SC state in RTG, using an exactly solvable model, namely the massive Dirac cone, to see if one can resolve discrepancy with experiment when only considering SC originating from flavor symmetric phase. We stress the exact mapping between the massive Dirac cone and the actual RTG is unnecessary, as the majority of the physics is captured by lack of state within the band gap. As such one expect Eqs. (4-5) in the manuscript to be good approximation for band edge IVC-SC in RTG as well, despite difference in band structure.

To justify the toy model, we reformulate the paragraph:

> Since only quasiparticles from the edge of lower energy IVC band majorly contribute to the formation of Cooper pairs, the lack of states within the band gap is well captured by the proposed toy model. As such, physical properties of IVC-SC, for example coherence length, can be approximated using result from band edge superconductivity of massive Dirac fermions. Given the effective mass gap of $m \approx 34.5$meV , with mean-field temperature $T_{\rm MF} = 120$mK and graphene typical velocity $\hbar v = 6.6 \times 10^{-1}$eVnm, the coherence length at base temperature $\approx 50$mK can be approximated using Eq. (5) to be 126nm, which is very close to the value (150-250nm) reported in the experiment [Nature (London) 598, 434–438 (2021)].

*10. The authors then perform numerical calculations on the microscopic model. The computation for $\Delta_{IVC}$ is a bit opaque to me. Are you setting $\Delta_{IVC}$ based on whether the normal state dopes into the blue band or not? The authors then say Eq. (17) is used to compute the transition temperature, but Eq. (17) is derived in the context of the the toy model in the Methods section. Is the claim that the same formula is applicable for the microscopic calculation?*

**Our response:** We thank the Referee for this point. We set the value of $\Delta_{\mathrm{IVC}}$, such that at charge carrier density $n \approx -1.8 \times 10^{12}\mathrm{cm}^{-2}$ there is a sharp disappearance of superconductivity, characterized by vanishing mean-field temperature. We also note Eq. (18-25) in the manuscript is applicable for any microscopic models, that is time-reversal symmetry $\varepsilon_{+s}(\mathbf{q}) = \varepsilon_{-\bar{s}}(-\mathbf{q})$, where the interaction is of form in Eq. (18) (previously Eq. (17)) in the manuscript:

$$H_{\mathrm{int}} = -U \sum_{s\lambda\lambda'} \int d^2\mathbf{r}\, a^{\dagger}_{+s\lambda}(\mathbf{r}) a^{\dagger}_{-\bar{s}\lambda'}(\mathbf{r}) a_{-\bar{s}\lambda'}(\mathbf{r}) a_{+s\lambda}(\mathbf{r}), \tag{10}$$

where $a_{\tau s\lambda}(\mathbf{r})$ is the quasiparticle annihilation operator of the valley $\tau$, spin $s$ and sublattice index $\lambda$, which is showcased in [Phys. Rev. Lett. 132, 026002 (2024)]. We agree that it should be clarified with sec. Method, the criteria of microscopic models such that Eq. (19-26) (previously 16(-23) in the manuscript are satisfied, beyond the toy model. In the revised manuscript, we modified as follows:

> We comment Eqs. (19-26) are applicable to any microscopic model, for example IVC state in RTG, that hosts two valleys with dispersion $\varepsilon_{+}(\mathbf{q}) = \varepsilon_{-}(-\mathbf{q})$, and has local attractive interaction between time-reversal copies of the same quasiparticle, which can be realized with onsite intervalley interaction given by Eq. (18).

*11. The authors claim that $H_{c2}$ is significantly modified by the quantum metric effect. I am not sure if the experimental phenomenology is rich enough to detect this effect. At $B = 0$, as we tune n, we can see a phase transition from SC to a metal. Within GL picture, this point corresponds to when the coefficient of the quadratic term is zero, thus signaling divergence coherence length. Therefore, the phase boundary seems to give divergent coherence length even in the absence of quantum metric contribution. Unless there is an extended regime in which coherence length is infinite, I find it difficult to distinguish the above scenario from quantum metric driven scenario.*

**Our response:** We thank the Referee for this point. For a band edge superconductor, at finite temperature, we note an interesting regime which is beyond the GL picture of a standard superconductor where the chemical potential $\mu$ lies within the middle of a band. At temperature $T = 0$, one may not expect SC when the chemical potential is in proximity to the edge of the band due to the almost vanishing density of states. However, SC can happen due to the band widening effect at finite temperature which effectively

enhances the charge carrier densities at the Fermi energy. In this case, the conventional GL coherence length, instead of scaling as $\sim 1/T_{\rm MF}$, which diverges at the phase boundary defined by $T_{\rm MF}=0$ when we tune the charge carrier $n$, scales as $\sim e^{-\alpha/T_{\rm MF}}/T_{\rm MF}$, as shown in Eq. (7) in the manuscript with $\alpha$ independent of $T_{\rm MF}$. As exponential decays faster than linear, the conventional GL coherence length thus remains finite as $T_{\rm MF}\rightarrow 0$ (i.e. approaching phase boundary).

We note the quantum metric contribution in Eq. (8) in the manuscript indeed diverges. Thus for a band edge superconductor, divergence at the phase boundary is only true for the quantum metric contribution, in contrast with expectation from standard GL theory. We comment that the divergence remains limited to the phase boundary. By extracting the scaling of the zero-temperature coherence length (from $H_{c2}$ experimentally) with the mean-field temperature, distinguishing between the standard GL scenario and quantum metric driven scenario remains possible.

Here are some miscellaneous comments

1. In method, the density of states of the quasi particles are said to "peak at the bottom of the conduction band", but isn't it the valence band?

**Our response:** We thank the Referee for this point. We have changed our wording to avoid confusion:

> As shown in Fig. 2(d), the density of states of the quasiparticles peak at the top of the valence band (the blue line) ...

Overall, I believe the manuscript contains a physically novel idea, but it suffers from somewhat cluttered presentation mixed with assumptions whose justifications are not made very clear. I encourage the authors to address the comments above in order to make a stronger case for a publication at Nature Communications.

**Our response:** We thank the Referee for their encouragement and many helpful questions. We have edited the manuscript according to the Referee's constructive comments.

List of changes (marked in red in the revised version):

(i) We clarified the role of the toy model in the theoretical calculation, and its connection to the numerical calculation.

(ii) We justified why the typical velocity of graphene heterostructure can be approximated by the fermi velocity of massless graphene monolayer.

(iii) We clarified our results on the quantum metric does not have significant implication in the current experimental setup, but instead serves as a prediction for future experiment performed at a lower temperature.

(iv) We included a new section "Details of fitting procedure for $U$ and $\Delta_{\mathrm{IVC}}$" in the revised supplementary material to address concern of Reviewer 2.

(v) We have corrected other typos.

## Reviewer 1

*The authors have addressed all of my comments and revised the manuscript accordingly. The current version can be published in my opinion.*

We thank the Reviewer for their precious time dedicated to reviewing our manuscript and their recommendation for publication.

## Reviewer 2

*I thank the authors for a thorough revision. However, while the paper has significantly improved in readability, I am still not sure if this paper is suitable for a publication at Nature Communications.*

We thank the Reviewer for their precious time dedicated to reviewing our manuscript and for their constructive feedback.

*The fundamental issue I have with the paper is that different levels of approximations are relatively freely mixed together, making it hard to understand the strength of each computation.*

We thank the Reviewer for the comment. However we have to we respectfully disagree with this statement. In our humble opinion, there is a misunderstanding of the connection between the toy model and our numerical results on the microscopic model encapsulating experimental observations. We emphasize the purpose of the toy model of massive Dirac cone is to obtain an analytical closed form that invoke qualitative insight on the phenomenology of superconductivity observed in Rhombohedral Trilayer Graphene. In the revised manuscript, we have clearly indicated the role of the toy model in our theoretical calculation. Without invoking the approximations made for the toy model, in section "*Numerical calculations on the microscopic model*", the numerical results which encapsulate experimental observations are obtained directly from Eqs. (19-25), using a realistic tight-binding model of Rhombohedral Trilayer Graphene. Qualitative agreement of our realistic model calculations with predictions from the toy model further confirms the validity of using the massive Dirac cone as a toy model.

In the new manuscript, we have highlighted the corresponding changes to avoid confusion.

*For example, in an early section, an estimate of coherence length is given by using the "average" fermi velocity of graphene materials. However, tunneling between layers is known to modify band dispersion drastically. It is unclear to me why approximating the fermi velocity by the value obtained from graphene linear dispersion is appropriate.*

We thank the Reviewer for the comment. We agree this is true near the $K(K')$ points, but it is not relevant to the experimental regime. The chemical potential is energetically far away from the $K(K')$ points, such that the effect due to tunneling between layers is insignificant. This validates our approximation of ignoring interlayer tunneling in our toy model as a good phenomenological theory. Our toy model is the simplest model, to the best of our knowledge, to showcase the discrepancy between the BCS theory

and the experiment. Last but not least, this concern has already been addressed in the revised manuscript following the first round. In Fig. 2(b), we directly use the tight-binding model of Rhombohedral Trilayer Graphene without the IVC order to showcase the discrepancy, which is consistent with the diagnosis using the BCS theory with the estimated fermi velocity. In the revised version, we have rewritten accordingly:

> where $v$ is the typical velocity of graphene heterostructure. We note the chemical potential corresponding to the experimental regime is energetically far away from the $K(K')$ points, thus the interlayer tunneling effect is negligible. Therefore, the typical velocity can be approximated by the fermi velocity of massless graphene monolayer $\hbar v \approx 6.6 \times 10^{-1}$eVnm.

*It is also not clear to me why a massive Dirac cone is an appropriate toy model for the phenomenology of IVC-based mechanism. Is the fact it's a Dirac cone important at all? It looks as though a lot of physics can be recovered by having normal quadratic bands whose band edge appears at a slightly offset chemical potential.*

We thank the Reviewer for the comment. We agree with the comment for the energetic contribution due to the similarity between the dispersion spectrum of massive Dirac cone and the quadratic bands near the band edge. Band edge superconductivity is, however, to our understanding, not previously studied in any literature at the time of writing, and the results illustrate unconventional superconductivity that is beyond the BCS limit, thus worth reporting. The recognition of band-edge superconductivity is key to explaining experimental measurements for Rhombohedral Trilayer Graphene phenomenologically. As we will showcase later, the key difference between the massive Dirac cone and the quadratic band lies in the quantum metric. We also predict that the quantum metric of the massive Dirac cone has a significant effect on the zero-temperature Ginzburg-Landau coherence length, which can be experimentally accessible upon further lowering the temperature. So this prediction can attract broader interest for both experiment and theory.

*There is an extensive discussion of quantum metric contribution to coherence length, but it's relevance to experimental data is unclear at best, given the minimal effect it has on the phase boundary in fig. 3(b).*

We thank the Reviewer for the comment. We agree that the quantum metric contribution does not have a significant effect on the coherence length near the phase boundary in the current experiment. However, as illustrated in Fig. 3(c), as we further decrease the temperature, we predict that the quantum metric contribution diverges while the conventional contribution remains finite for the zero-temperature Ginzburg-Landau coherence length at the phase boundary. This serves as smoking-gun evidence for future experiments to be performed at a lower temperature, allowing distinction between conventional and quantum metric contributions for $H_{c2}$. In the revised manuscript, this is emphasized in the conclusion:

We want to comment on the role of quantum metric, where the quantum metric only has a correction effect in the current experimental setup, on the superconducting coherence length observed in RTG. However, near the phase transition from SC-IVC to IVC state, the quantum metric contribution increases rapidly and diverges at the phase boundary, while the conventional contribution remains finite. This serves as smoking-gun evidence for future experiments to be performed at a lower temperature, allowing distinction between conventional and quantum metric effect for $H_{c2}$.

*Additionally, even if quantum metric were important, the paper does not really justify why the quantum metric of a Dirac cone is a better approximation for the band edge than a simple quadratic model.*

We thank the Reviewer for the comment. We want to stress that in the standard quadratic model, the Bloch state is trivial, and thus the quantum metric is always vanishing. Via a band projection, it is possible to obtain an effective quadratic model with quantum metric. However, this in general requires diagonalization of a complicated multiband system. Instead, we should employ a minimalistic model that contains the essence of the realistic material. This justifies the usage of massive Dirac cone to introduce non-trivial quantum metric, due to the resemblance with graphene heterostructure. The usage of the massive Dirac cone, thus allows qualitative study on difference between conventional and quantum metric contributions, in particular, near phase boundary as discussed before. The conclusion is later confirmed with the realistic model calculation, justifying massive Dirac cone as a simple yet good toy model.

*Overall, while the proposed mechanism is interesting, the theoretical analysis is not carried out at the level of clarity and rigor required for a publication at Nature communications.*

We thank the Reviewer for expressing interest in our proposed mechanism. We sincerely hope our comments above has further clarified and consolidated our theoretical calculations, justifying publication at Nature Communications.

*Here are point by point comments.*

We thank the Reviewer for the point-by-point comments, which we address below:

*1. "Instead, we propose that when in close vicinity with the phase transition from IVC to a flavor symmetric state, where the chemical potential cuts the band edge of the lower energy IVC quasiparticle band, would increase in density of state thus favoring direct pairing between IVC quasiparticles, where the IVC order*

*parameter remains intact." this seems to have subject-object mismatch.*

We thank the Reviewer for the suggestion on the writing. In the revised version, we have rewritten accordingly:

> Instead, when in close vicinity with the phase transition from IVC to a flavor symmetric state, where the chemical potential cuts the band edge of the lower energy IVC quasiparticle band, there is an increase in density of state for the IVC quasiparticles. This favors direct pairing between IVC quasiparticles, leading to the IVC-SC phase, where the IVC order parameter remains intact.

*2. "Focusing on SC1, at $n \approx -1.85 \times 10^{12}\text{cm}^{-2}$, corresponding to chemical potential of $\mu \approx -43.4\text{meV}$, the temperature is maximum, at $T_{MF} \approx$ 120mK." You probably meant to say something like "the transition temperature takes the maximum value". Also, $T_{MF}$ is probably supposed to be $T_c$, since it's not a mean-field value.*

We thank the Reviewer for the suggestion. In the revised version, we have rewritten accordingly:

> Focusing on SC1, at $n \approx -1.85 \times 10^{12}\text{cm}^{-2}$, corresponding to chemical potential of $\mu \approx -43.4\text{meV}$, the transition temperature takes the maximal value of $T_c \approx 120\text{mK}$.

*3. "Since only quasiparticles from the edge of lower energy IVC band majorly contribute to the formation of Cooper pairs, the lack of states within the band gap is well captured by the proposed toy model." The use of the word "band gap" is confusing in this context, since there is still finite density of states arising from the other band. I think a better terminology is necessary here.*

We thank the Reviewer for this point. We agree that a change of the terminology of "band gap" can avoid confusion, and we should use "direct band gap" instead. Indeed the chemical potential where superconductivity is observed crosses two quasiparticle bands colored as blue and red in Fig. 2(d). However, the density of states of the red band is much lower than that of the blue band, thus the contribution of the red band to the superconductivity is negligible. This motivates projection onto the blue band.

*4. "Using the fact that at charge career density ...... we can deduce $\Delta_{\text{IVC}} = 7$ meV". This was not clear to me. Is it obtained from some fitting procedure?*

We thank the Reviewer for this point. The IVC order parameter determines the quasiparticle band structure. From the quasiparticle band structure, we identify the charge carrier density at the band edge, which

corresponds to the superconducting phase boundary. Then by tuning the order parameter, we can match the charge carrier density with the experimentally observed value of $n \approx -1.8 \times 10^{12}\text{cm}^{-2}$. We have added a section titled "Details of fitting procedure for $U$ and $\Delta_{\text{IVC}}$", at the end of the appendix, to provide details in the fitting procedures of $\Delta_{\text{IVC}}$, and of $U$ previously requested by the Reviewer.

*5. "The mean-field temperature calculated from the IVC state, in general, agrees with the experimental result, both reaching a maximum of at" Remove second to last "of"*

We thank the Reviewer for this point. We have corrected the mistake accordingly.
